# Development and validation of a metabolite score for red meat intake: an observational cohort study and randomized controlled dietary intervention

**DOI:** 10.1093/ajcn/nqac094

**Published:** 2022-06-27

**Authors:** Chunxiao Li, Fumiaki Imamura, Roland Wedekind, Isobel D Stewart, Maik Pietzner, Eleanor Wheeler, Nita G Forouhi, Claudia Langenberg, Augustin Scalbert, Nicholas J Wareham

**Affiliations:** Medical Research Council Epidemiology Unit, Institute of Metabolic Science, University of Cambridge School of Clinical Medicine, Cambridge, United Kingdom; Medical Research Council Epidemiology Unit, Institute of Metabolic Science, University of Cambridge School of Clinical Medicine, Cambridge, United Kingdom; Nutrition and Metabolism Branch, International Agency for Research on Cancer, World Health Organization, Lyon, France; Medical Research Council Epidemiology Unit, Institute of Metabolic Science, University of Cambridge School of Clinical Medicine, Cambridge, United Kingdom; Medical Research Council Epidemiology Unit, Institute of Metabolic Science, University of Cambridge School of Clinical Medicine, Cambridge, United Kingdom; Computational Medicine, Berlin Institute of Health at Charité–Universitätsmedizin Berlin, Berlin, Germany; Medical Research Council Epidemiology Unit, Institute of Metabolic Science, University of Cambridge School of Clinical Medicine, Cambridge, United Kingdom; Medical Research Council Epidemiology Unit, Institute of Metabolic Science, University of Cambridge School of Clinical Medicine, Cambridge, United Kingdom; Medical Research Council Epidemiology Unit, Institute of Metabolic Science, University of Cambridge School of Clinical Medicine, Cambridge, United Kingdom; Computational Medicine, Berlin Institute of Health at Charité–Universitätsmedizin Berlin, Berlin, Germany; Nutrition and Metabolism Branch, International Agency for Research on Cancer, World Health Organization, Lyon, France; Medical Research Council Epidemiology Unit, Institute of Metabolic Science, University of Cambridge School of Clinical Medicine, Cambridge, United Kingdom

**Keywords:** metabolomics, meat, prediction, biomarker, diabetes

## Abstract

**Background:**

Self-reported meat consumption is associated with disease risk but objective assessment of different dimensions of this heterogeneous dietary exposure in observational and interventional studies remains challenging.

**Objectives:**

We aimed to derive and validate scores based on plasma metabolites for types of meat consumption. For the most predictive score, we aimed to test whether the included metabolites varied with change in meat consumption, and whether the score was associated with incidence of type 2 diabetes (T2D) and other noncommunicable diseases.

**Methods:**

We derived scores based on 781 plasma metabolites for red meat, processed meat, and poultry consumption assessed with 7-d food records among 11,432 participants in the EPIC-Norfolk (European Prospective Investigation into Cancer and Nutrition-Norfolk) cohort. The scores were then tested for internal validity in an independent subset (*n* = 853) of the same cohort. In focused analysis on the red meat metabolite score, we examined whether the metabolites constituting the score were also associated with meat intake in a randomized crossover dietary intervention trial of meat (*n* = 12, Lyon, France). In the EPIC-Norfolk study, we assessed the association of the red meat metabolite score with T2D incidence (*n* = 1478) and other health endpoints.

**Results:**

The best-performing score was for red meat, comprising 139 metabolites which accounted for 17% of the explained variance of red meat consumption in the validation set. In the intervention, 11 top-ranked metabolites in the red meat metabolite score increased significantly after red meat consumption. In the EPIC-Norfolk study, the red meat metabolite score was associated with T2D incidence (adjusted HR per SD: 1.17; 95% CI: 1.10, 1.24).

**Conclusions:**

The red meat metabolite score derived and validated in this study contains metabolites directly derived from meat consumption and is associated with T2D risk. These findings suggest the potential for objective assessment of dietary components and their application for understanding diet–disease associations.

The trial in Lyon, France, was registered at clinicaltrials.gov as NCT03354130.

See corresponding editorial on page 295.

## Introduction

Meat is an important component of the human diet and high consumption is a risk factor for many noncommunicable diseases, including type 2 diabetes (T2D) (1–5). However, meat consumption is a heterogeneous exposure and assessing total meat intake and specific subtypes such as red meat, processed meat, and poultry in epidemiologic studies that evaluate its influence on health outcomes remains challenging.

Metabolite profiling is a promising approach for quantifying habitual meat intake and can be a complementary approach to self-reported dietary assessment methods (e.g., FFQs or dietary records) (6, 7). Diet is an important determinant of the plasma metabolome and a previous study estimated that it accounts for 50% of the explainable variance, compared with 2% of the variance explained by lifestyle factors, including smoking status, exercise time, etc. (7). Measurement of metabolites as a complement to self-reported assessment methods has other theoretical advantages, including diminishing social desirability bias and recall bias, and greater comparability across populations (8, 9).

Several individual metabolites have previously been reported to be significantly associated with different types of meat consumption (10–13). However, few studies have examined how combinations of metabolites can predict meat consumption. Cuparencu et al. (12) reported that a combination of 6 metabolite biomarkers were able to assign people to a binary classification of red meat consumption in a 2-d feeding trial. However, the study was small and the result may be liable to overfitting.

In the current study, we aimed to develop and test metabolite scores for different types of meat consumption by combining 781 blood metabolites in the EPIC-Norfolk (European Prospective Investigation into Cancer and Nutrition-Norfolk) cohort and then to take forward the red meat metabolite score to potential replication in a short-term randomized controlled trial (RCT) that measured metabolites after a red meat and a nonmeat diet. Finally, we tested whether the meat metabolite score was associated with the risk of incident T2D and other noncommunicable diseases to explore the potential utility of the score in understanding disease risk.

## Methods

### Data source and study design

**FIGURE 1:**
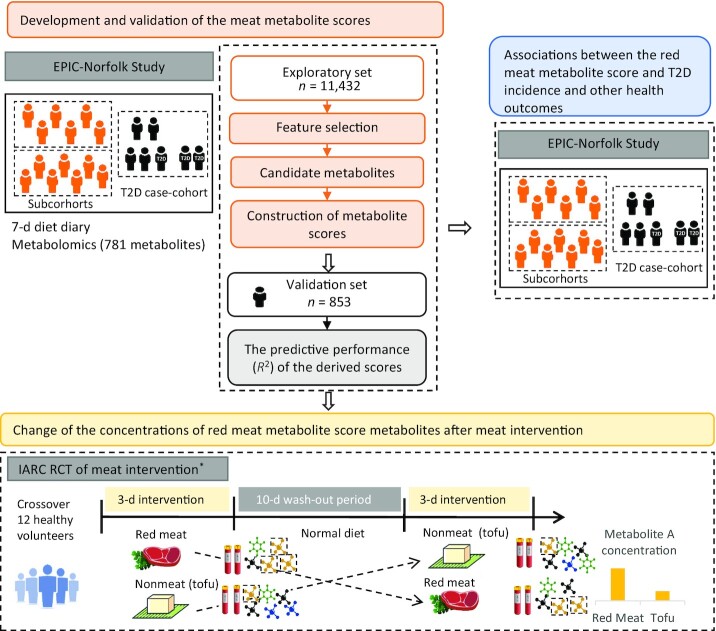
Flowchart for the overall analytic approach for development and validation of the meat metabolomics score. *The visualization simplifies the design of the RCT because only 2 out of 5 arms are shown. EPIC-Norfolk, European Prospective Investigation into Cancer and Nutrition-Norfolk; IARC, International Agency for Research on Cancer; RCT, randomized controlled trial; T2D, type 2 diabetes.

The overall design of the project included a derivation and validation phase in an observational study, a test of change in an RCT, and a test of association with incident health outcomes in a prospective study, as shown in **Figure 1**.

### Observational data for the derivation and validation of the metabolite scores: the EPIC-Norfolk study

We developed and validated the metabolite scores for 3 types of meat consumption (red meat, processed meat, and poultry) using baseline data from the EPIC-Norfolk study, which originally recruited 25,639 males and females aged 40–79 y between 1993 and 1998 in the United Kingdom. Details of the recruitment procedures and data collection have been described previously (14). Briefly, baseline characteristics for all participants were collected, including sociodemographic factors (age, sex, and education level), health behaviors (smoking status, alcohol drinking, and physical activity), and dietary measures. Blood samples were collected at baseline and stored in liquid nitrogen at −175°C. The EPIC-Norfolk study was approved by the Norwich Local Ethics Committee (REC Ref: 98CN01); all participants gave their informed written consent before entering the study.

We developed metabolite scores for different types of meat consumption in an exploratory set which included 11,432 participants who had both untargeted metabolomics and dietary data. We excluded from this exploratory data set individuals who were part of a nested case-cohort study for incident T2D; those with extreme energy intake measures (<500 and >3500 kcal/d for females, <800 and >4200 kcal/d for males); or those with prevalent diabetes at baseline.

Participants from the subcohort of an independent nested T2D case-cohort study (15) were used as a validation set, which included 853 participants after exclusions.

### Metabolomics measurement and data processing in the EPIC-Norfolk study

We measured untargeted metabolomics using ultra-performance LC–tandem MS on the Metabolon DiscoveryHD4® platform from plasma samples collected at baseline. The measurement of metabolites was performed in 3 subsets in March 2015, January 2016, and March 2017 successively. The data quality control and processing methods have been described previously (16) and are summarized in the **Supplemental Methods**. After data quality control and data management, the 3 subsets included 1503, 5992, and 5980 individuals, in which 944, 1168, and 1219 metabolites were measured, respectively, and 781 metabolites were identical across all subsets.

Before analysis, we undertook the following steps for each metabolite within each subset: log-transformation, replacement of outliers with 5 SDs from the mean (Winsorization), and standardization to a mean of 0 and SD of 1. For metabolite concentrations below the limit of detection, we imputed them with the lowest measured value of that metabolite (17). The different subsets in the exploratory data set were measured at different time points, and we adjusted for measurement time period in the regression analysis.

### Assessment of meat consumption in the EPIC-Norfolk study

Meat consumption and other dietary exposures were assessed with a 7-d diet diary (7dDD) as documented previously (18). Briefly, on the day of a baseline assessment that included blood sampling, participants were asked to record everything they ate (food types, amounts, brands, recipes, and cooking methods) prospectively for the following 7 consecutive days. The dietary information was then processed into food and nutrient data by programs and databases (DINER and DINERMO) using standard protocols (18, 19). The meat-related categories were all disaggregated from composite dishes including red meat (unprocessed beef, lamb, pork, veal, rabbit, venison, etc.), processed meat (bacon, ham, sausages, etc., smoked, cured, salted, or chemically preserved), and poultry (chicken, turkey, goose, duck, guinea fowl, pheasant, etc.) in the unit of grams per day. Participants were also asked whether they followed a special diet (vegetarian, other diet, or no special diet).

### Development and validation of metabolite scores of self-reported red meat, processed meat, and poultry consumption

In the EPIC-Norfolk study, 781 metabolites were evaluated simultaneously for the prediction of red meat consumption. In the exploratory set, we applied elastic net regression (20) with a bootstrap approach (21, 22) to select a combination of metabolites for prediction of red meat consumption; and ridge regression (23) to estimate penalized weights of these candidate metabolites (Supplemental Methods). We applied the weights of all candidate metabolites and constructed a metabolite score for each individual in both of the derivation and validation data sets. The score was standardized to a mean of 0 and SD of 1 for further analysis. The metabolite scores for processed meat and poultry were derived and tested using the same process.

### RCT of meat consumption

Given the availability of trial-based data for meat consumption, we further investigated associations of metabolites in the score from the observational EPIC-Norfolk study with red meat consumption in an RCT previously conducted in Lyon, France in 2018 (NCT03354130). The details of this RCT have been reported previously (24). In brief, 12 healthy adults consumed in random order 5 different foods (fried pork, hot dogs, bacon, salami, and tofu) as part of a controlled diet. For this analysis, we examined the differences in metabolite concentrations between the fried pork (unprocessed red meat) and tofu control arms. In this trial, fasting plasma samples were collected during the morning after the last meal of each test period. Participants provided informed consent and procedures were carried out according to the Declaration of Helsinki. The study was approved by the International Agency for Research on Cancer (IARC) Ethics Committee (IEC Project 17-12).

### Test of candidate metabolites of red meat intake in the RCT

We assessed whether metabolites that were part of the metabolite score for red meat intake were increased after intake of fried pork (red meat) compared with tofu in the RCT. **Supplemental Figure 1** shows the process of identification of metabolites that make up the red meat metabolite score in the RCT. First, we focused on metabolites that had been annotated successfully in the IARC laboratory and had a positive coefficient in the metabolite score. Corresponding signal intensities were extracted with Agilent Profinder 10.0 (Agilent Technologies) using the find-by-formula method [(M + H)^+^ and (M − H)^−^ ions only; exact mass: ± 8 ppm; retention time: ± 0.05 min). Metabolites were carried forward for statistical analysis if they were detected in >75% of the samples collected after pork intake. Then we used paired Welch's *t* tests to assess whether metabolites were significantly (*P* < 0.05) elevated in plasma samples collected after pork intake compared with tofu intake. Second, for metabolites not previously identified in the IARC laboratory, we extracted only those with a coefficient > 1.0 in the meat intake score from the raw data by formula only to test for their increase in plasma samples after pork intake. Compounds were confirmed by comparison of MS/MS spectra to those in the literature (annotation confidence level 2 or 3) (25).

### Prospective cohort analysis of the association of the red meat metabolite score with incident disease outcomes in the EPIC-Norfolk study

We examined the association of the red meat metabolite score and the relevant self-reported consumption parameter with the risk of incident T2D in a case-cohort study nested in the EPIC-Norfolk cohort (15). This comprised a total of 659 incident cases of T2D and a comparison subcohort of 846 participants, which by design had an overlap of 27 individuals with the case set, after we excluded participants who had extreme energy intake measures or missing covariate data.

### Ascertainment of T2D cases in the EPIC-Norfolk study

Incident cases of T2D were ascertained by reviewing evidence from multiple sources, including self-report, linkage to primary and secondary care registers, medication use from drug registers, hospital admissions, and mortality data. All self-reported cases were verified with independent evidence. Person time of follow-up was determined from the date of baseline assessment to the date of diagnosis, date of death, or 31 December, 2006, whichever came first.

### Assessment of covariates in the EPIC-Norfolk study

Information about health behaviors and clinical risk factors was collected by trained nurses during a health check at baseline. Information obtained included age, sex, education level (primary school or no qualifications, middle school or equivalent, high school or equivalent, college degree and above), smoking status (never, former, and current smokers), alcohol drinking (g/d), physical activity (inactive, moderately inactive, moderately active, active), height (m), weight (kg), and other food group consumption (g/d; fruits, vegetables, fatty fish, white fish, dairy, legumes, nuts, eggs, and sugar-sweetened beverages). BMI was calculated as weight divided by the square of height (kg/m^2^). Total energy intake was calculated from the 7dDD.

### Statistical methods for assessment of the association with incident T2D

We analyzed the association of a standardized metabolite score for red meat consumption with incident T2D in the case-cohort study using Prentice-weighted Cox regression (26) to estimate the HR for T2D and its 95% CI per SD of the exposure.

We considered the effect of potential confounders in a model adjusting for age and sex, and then further adjusted for education, smoking status, alcohol drinking, BMI, and dietary factors (consumption of fruits, vegetables, fatty fish and white fish, sugar-sweetened beverages, dairy, legumes, nuts, eggs, and total energy intake). For alcohol drinking and BMI, we included their linear and squared terms to account for their potential nonlinear associations with each outcome.

### Ascertainment of other noncommunicable disease outcomes in the EPIC-Norfolk study

We ascertained the incident outcomes of 6 health conditions including cardiovascular diseases (including ischemic heart disease, hemorrhagic stroke, cerebral stroke, heart failure, and atrial fibrillation), gastrointestinal cancers (including colon cancer, rectal cancer, stomach cancer), liver disease, renal disease, fractures, and deaths due to any causes (16). Outcome data were obtained by linkage to Hospital Episode Statistics, the cancer registry, and the Office of National Statistics. Follow-up ended on 31 March, 2016. Prevalent and incident cases for each disease were identified with the International Classification of Diseases 10th revision as listed in **Supplemental Table 1**.

### Statistical methods for assessment of the association with multiple disease outcomes

In an exploratory analysis we tested the association of the red meat metabolite score with incident health outcomes using standard Cox regression after excluding the prevalent cases for each clinical outcome (see Supplemental Table 1). We adjusted for the same sets of potential confounders as considered in the association with T2D.

## Results

### Baseline characteristics and meat consumption of study participants in the EPIC-Norfolk study

**Table 1** shows the baseline characteristics of the participants in the exploratory and validation sets within the EPIC-Norfolk study. Among the 11,432 participants in the exploratory set, 46% were male and the mean ± SD age at baseline was 59.6 ± 9.0 y. The mean ± SD meat consumption in g/d was 34.4 ± 29.3 for red meat, 22.5 ± 21.0 for processed meat, and 24.8 ± 27.5 for poultry. The characteristics in the validation set (*n* = 853) were broadly similar to those in the exploratory set.

**TABLE 1:** Baseline characteristics of the study participants used for development and validation of meat metabolite scores in the European Prospective Investigation into Cancer and Nutrition-Norfolk study^1^

	Exploratory set (*n* = 11,432)	Validation set (*n* = 853)
Age, y	59.6 ± 8.96	59.0 ± 9.40
Female	6204 (54)	494 (58)
Red meat intake, g/d	34.4 ± 29.3	33.6 ± 29.1
Processed meat intake, g/d	22.5 ± 21.0	21.7 ± 19.7
Poultry intake, g/d	24.8 ± 27.5	26.0 ± 25.5
Education		
No	4345 (38)	326 (38)
O-level	1155 (10)	79 (9)
A-level	4541 (40)	330 (39)
Degree	1385 (12)	117 (14)
Missing	6 (0.1)	1 (0.1)
Smoking		
Current	1290 (11)	112 (13)
Former	4826 (42)	329 (39)
Never	5224 (46)	407 (48)
Missing	92 (0.8)	5 (0.6)
Alcohol intake, g/d	11.9 ± 17.8	11.6 ± 16.6
PA		
Inactive	3325 (29)	238 (28)
Moderately inactive	3243 (28)	246 (29)
Moderately active	2658 (23)	206 (24)
Active	2206 (19)	163 (19)
BMI, kg/m^2^		
Mean ± SD	26.1 ± 3.67	26.1 ± 3.71
Missing	16 (0.1)	2 (0.2)
Total energy, kcal/d	1950 ± 526	1940 ± 517
Fruit intake, g/d	166 ± 126	168 ± 125
Vegetable intake, g/d	152 ± 76.9	150 ± 68.6
Fatty fish intake, g/d	12.3 ± 20.4	13.3 ± 22.3
White fish intake, g/d	15.5 ± 18.5	15.9 ± 17.6
Legumes intake, g/d	28.6 ± 30.2	26.7 ± 26.9
Nuts intake, g/d	2.31 ± 6.51	2.18 ± 5.64
Dairy intake, g/d	222 ± 146	217 ± 142
Egg intake, g/d	14.3 ± 17.4	14.0 ± 17.0
Sugar-sweetened beverage intake, g/d	32.9 ± 78.6	30.8 ± 65.5

1 Values are mean ± SD for continuous variables and *n* (%) for categoric variables. PA, physical activity.

### Development and validation of metabolite scores for meat consumption

**FIGURE 2:**
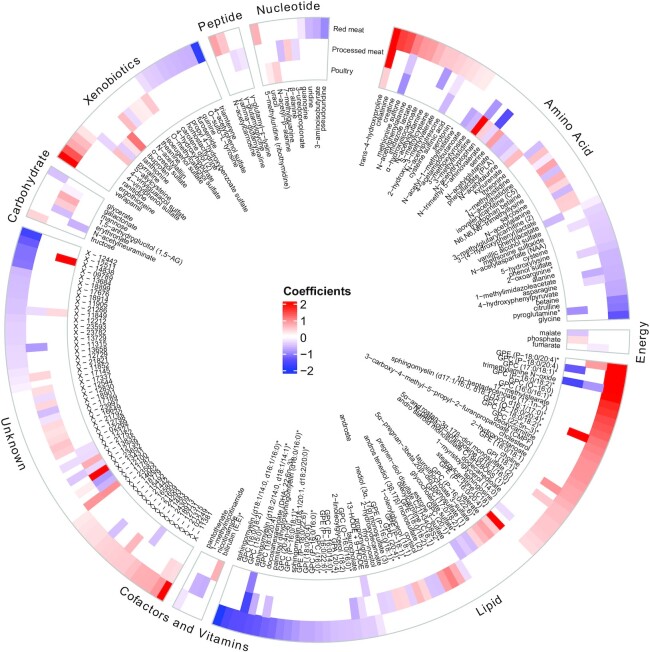
Coefficients of metabolites with self-reported red and processed meat and poultry intake: the European Prospective Investigation into Cancer and Nutrition-Norfolk study (*n* = 11,432). Metabolites were classifed by metabolic pathway (16). The colors represent the coefficients (weights) of each metabolite in each metabolite score; red means positive association and blue means negative association. *The metabolite was annotated based on in silico predictions, which indicates the compound has not been confirmed based on a standard but its identity is confident. GPA, glycerol-3-phosphate; GPC, glycerophosphocholine; GPE, glycerophosphoethanolamine; GPI, glycosylphosphatidylinositol; HODE, hydroxyoctadecadienoic acid.

**FIGURE 3:**
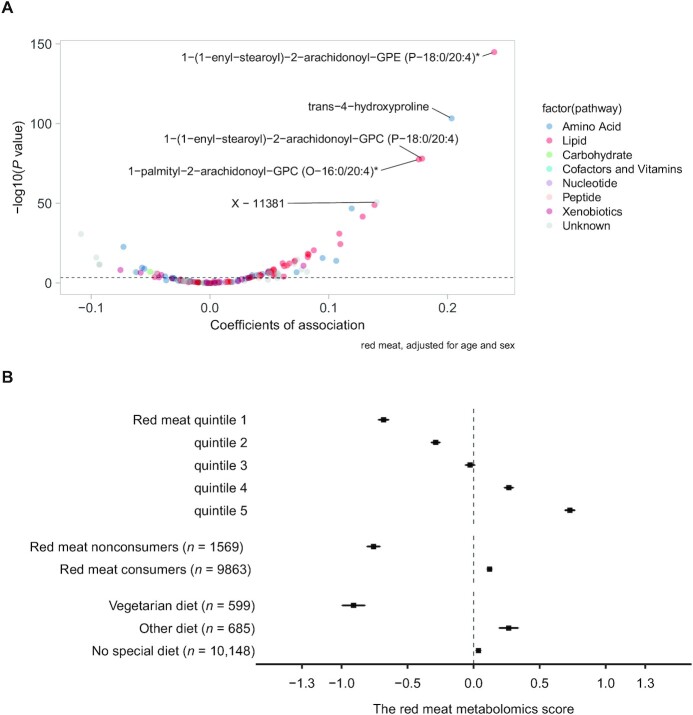
Volcano plot of candidate metabolites for red meat intake (*n* = 139) against self-reported red meat intake and comparison of the red meat metabolite score across different categories of meat consumer groups: the European Prospective Investigation into Cancer and Nutrition-Norfolk study (*n* = 11,432). (A) The top 5 metabolites with the strongest association with self-reported red meat intake after adjustment for age and sex are annotated in the volcano plot. *The metabolite was annotated based on in silico predictions, which indicates the compound has not been confirmed based on a standard but its identity is confident. (B) A red meat nonconsumer was defined as a participant with red meat consumption equal to 0 (*n* = 1569) and a red meat consumer was a participant with red meat consumption > 0 (*n* = 9863). Participants who reported consuming a vegetarian diet, other diet, or no special diet were identified from self-reported questionnaires. GPC, glycerophosphocholine; GPE, glycerophosphoethanolamine.

In the exploratory set in the EPIC-Norfolk study, 139 metabolites were identified to be associated with red meat consumption, and they were assembled into a composite red meat metabolite score. This score was made up of 49 (19.3%) lipids and 30 (22.2%) amino acids, other metabolite classes such as xenobiotics (*n* = 14, 12.5%), and 36 (18.4%) unknown metabolites (**Figure 2**, **Supplemental Table 2**). The top 5 metabolites with positive coefficients were 1-(1-enyl-stearoyl)-2-arachidonoyl-glycerophosphoethanolamine (GPE) (P-18:0/20:4), 1-(1-enyl-stearoyl)-2-arachidonoyl-glycerophosphocholine (GPC) (P-18:0/20:4), 1-margaroyl-2-oleoyl-GPC (17:0/18:1), trans-4-hydroxyproline, and Verapamil. The derived metabolite score for red meat consumption achieved an explained variance of 24% and 17% in the exploratory and validation sets, respectively. The metabolite score for red meat intake was associated with quintiles of self-reported meat intake (**Figure 3**). It was also significantly higher in the subgroups of self-reported red meat consumers and nonvegetarians than in nonconsumers of red meat and vegetarians, respectively.

The metabolite scores for processed meat consumption and poultry consumption consisted of 82 and 49 predictive metabolites, respectively, and were made up predominantly of lipids and amino acids (Figure 2, **Supplemental Tables 3** and **4**). Figure 2 shows the overlapping and distinct sets of metabolites that were associated with red meat, processed meat, and poultry consumption. Six metabolites were included in all 3 metabolite scores: trans-4-hydroxyproline, trimethylamine N-oxide (TMAO), methionine sulfone, sphingomyelin (d18:2/14:0, d18:1/14:1), N-acetylputrescine, and X-11849. Overall, the 7dDD meat intake variance explained by the corresponding metabolite scores in the validation set was 15% for processed meat and 13% for poultry (**Supplemental Figure 2**). **Supplemental Tables 5–7** show the details of parameter optimization and metabolite selection in the bootstrapping process. In additional analyses, we calculated estimated glomerular filtration rate (eGFR) as an indicator of renal function and assessed the associations between red meat intake and each of the 781 common metabolites after statistical adjustment for eGFR, showing that the coefficients were unchanged from the analysis that was not adjusted for eGFR.

### Associations of metabolites in the red meat metabolite score with meat intake in an RCT

For the metabolites that were part of the metabolite score for red meat intake, we used untargeted plasma metabolomics data from a meat RCT to investigate the differences of metabolite concentrations after a 3-d red meat intervention compared with a nonmeat diet. Of the 50 known metabolites positively associated with self-reported red meat consumption in the observational EPIC-Norfolk study, 11 were identified in the RCT and significantly increased after fried pork (red meat) intake compared with tofu: several glycerophospholipids, 4-hydroxyproline, TMAO, creatine, deoxycarnitine, and stearoylcarnitine (**Table 2**, **Supplemental Figures 3** and **4**). **Supplemental Figure 5** shows the correlations between these top-ranked metabolites and types of meat consumption in the EPIC-Norfolk study. **Supplemental Table 8** reports the correlations between these top-ranked metabolites. Of the top 8 metabolites that had the highest coefficients in the red meat metabolite score in the EPIC-Norfolk study, 6 were validated in the RCT.

**TABLE 2:** Metabolites from the red meat metabolomics score that were positively associated with red meat consumption in both the EPIC-Norfolk and the randomized crossover trial^1^

Name	Formula	Fold-change^2^	*P* value	Chromatographic method^3^	Retention time, min	Confidence level of identification^4^	MS fragments for identification	Rank^5^
1-(1-Enyl-stearoyl)-2-arachidonoyl-GPE (P-18:0/20:4)	C_43_H_78_NO_7_P	2.52	1.36 × 10^−6^	RP	9.04, 9.43	Level 2	361.2741	1
							611.5296	
							392.2934	
1-(1-Enyl-stearoyl)-2-arachidonoyl-GPC (P-18:0/20:4)	C_46_H_84_NO_7_P	2.00	6.69 × 10^−6^	RP	9.1	Level 3	184.0733	2
4-Hydroxyproline	C_5_H_9_NO_3_	6.27	1.06 × 10^−4^	HILIC	5.74	Level 1	68.0498	4
							86.0601	
TMAO	C_3_H_9_NO	1.56	6.30 × 10^−3^	HILIC	3.62	Level 1	42.0329	7
1-(1-Enyl-palmitoyl)-2-linoleoyl-GPC (P-16:0/18:2)	C_42_H_80_NO_7_P	1.32	1.94 × 10^−4^	RP	8.97	Level 3	184.0733	8
1-Palmityl-GPC (O-16:0)	C_24_H_52_NO_6_P	2.01	3.64 × 10^−6^	RP	7.18	Level 2	104.1072	9
							184.0770	
							341.3025	
Creatine	C_4_H_9_N_3_O_2_	1.50	4.88 × 10^−2^	RP	0.7	Level 1	44.0482	13
							90.0538	
1-Palmityl-2-arachidonoyl-GPC (O-16:0/20:4)	C_44_H_82_NO_7_P	2.44	4.30 × 10^−6^	RP	9.04	Level 3	184.0733	17
1-(1-Enyl-stearoyl)-2-linoleoyl-GPC (P-18:0/18:2)	C_44_H_84_NO_7_P	1.96	1.00 × 10^−3^	RP	9.19	Level 3	184.0733	18
Deoxycarnitine	C_7_H_15_NO_2_	1.23	6.12 × 10^−3^	HILIC	5.18	Level 2	43.0179	21
							60.0811	
							87.0445	
Stearoylcarnitine	C_25_H_49_NO_4_	1.52	7.36 × 10^−3^	RP	6.47	Level 1	85.0277	57
							60.0813	

1 EPIC-Norfolk, European Prospective Investigation into Cancer and Nutrition-Norfolk; GPC, glycerophosphocholine; GPE, glycerophosphoethanolamine; HILIC, hydrophilic interaction liquid chromatography; RP, reverse-phase chromatography.

2 Fold-change in signal intensity in the randomized controlled trial after fried pork intake compared with the tofu diet. Paired Welch's *t* tests were used to evaluate whether metabolites were significantly increased after pork intake compared with tofu intake. Supplemental Figure 3 shows the variation of metabolite intensity after consumption of pork compared with tofu.

3 Supplemental Figure 4 shows the chromatographic tracing of selected metabolites in the blood after consumption of pork compared with tofu.

4 Level of confidence in metabolite identification according to Sumner et al. (25): level 1, matching of mass, retention time, and mass fragmentation pattern with authentic chemical standard; level 2, matching of accurate mass and mass fragmentation pattern with the corresponding compound in a database; level 3, matching of mass and fragmentation pattern with the corresponding compound in a database—due to the nonspecific fragment, only the functional group, but not the length of each carbon chains can be determined.

5 Coefficient rank out of 139 metabolites in the red meat metabolite score in the EPIC-Norfolk study.

### Association of the red meat metabolite score with T2D

**Table 3** presents the baseline characteristics of the participants in the T2D case-cohort. In the subcohort, participants with higher metabolite scores of red meat consumption were more likely to be male; current smokers; have higher BMI; higher consumption of alcohol, legumes, sugar-sweetened beverages, and total energy; and have lower amounts of fruit and fish consumption than participants with lower metabolite scores.

**TABLE 3:** Baseline characteristics of the study participants of the T2D case-cohort in the European Prospective Investigation into Cancer and Nutrition-Norfolk cohort^1^

	Subcohort						T2D cases (*n* = 659)
	Total (*n* = 846)	Q1 (*n* = 169)	Q2 (*n* = 169)	Q3 (*n* = 169)	Q4 (*n* = 169)	Q5 (*n* = 170)	
Red meat intake, g/d	33.6 ± 29.1	20.7 ± 20.7	25.8 ± 22.2	29.4 ± 22.7	40.9 ± 28.0	51.3 ± 37.8	39.3 ± 30.6
Age, y	59.0 ± 9.4	59.3 ± 9.5	58.5 ± 9.4	58.9 ± 9.5	59.4 ± 9.3	58.7 ± 9.3	61.8 ± 8.3
Female	489 (58)	133 (79)	115 (68)	89 (53)	90 (53)	62 (36)	275 (42)
Education							
No	321 (38)	69 (41)	62 (37)	59 (35)	72 (43)	59 (35)	309 (47)
O-level	79 (9)	20 (12)	14 (8)	17 (10)	11 (7)	17 (10)	54 (8)
A-level	329 (39)	60 (36)	64 (38)	72 (43)	66 (39)	67 (39)	229 (35)
Degree	117 (14)	20 (12)	29 (17)	21 (12)	20 (12)	27 (16)	67 (10)
Smoking							
Current	112 (13)	15 (9)	17 (10)	19 (11)	27 (16)	34 (20)	79 (12)
Former	328 (39)	53 (31)	59 (35)	63 (37)	69 (41)	84 (49)	320 (49)
Never	406 (48)	101 (60)	93 (55)	87 (51)	73 (43)	52 (31)	260 (39)
Alcohol intake, g/d	11.7 ± 16.7	6.33 ± 8.71	11.0 ± 16.3	12.8 ± 17.0	10.6 ± 15.7	17.8 ± 21.2	11.4 ± 19.0
PA							
Inactive	234 (28)	54 (32)	37 (22)	51 (30)	42 (25)	50 (29)	290 (44)
Moderately inactive	244 (29)	46 (27)	65 (38)	39 (23)	46 (27)	48 (28)	157 (24)
Moderately active	206 (24)	39 (23)	37 (22)	45 (27)	44 (26)	41 (24)	122 (19)
Active	162 (19)	30 (18)	30 (18)	34 (20)	37 (22)	31 (18)	90 (14)
BMI, kg/m^2^	26.0 ± 3.71	25.3 ± 3.37	26.1 ± 3.85	26.6 ± 3.79	26.0 ± 3.72	26.2 ± 3.71	29.6 ± 4.51
Total energy, kcal/d	1940 ± 516	1790 ± 434	1850 ± 444	1980 ± 543	2030 ± 560	2060 ± 537	1940 ± 538
Processed meat intake, g/d	21.7 ± 19.7	16.3 ± 19.2	19.1 ± 17.2	19.5 ± 17.2	25.7 ± 21.5	28.0 ± 20.9	25.1 ± 21.1
Poultry intake, g/d	25.8 ± 25.3	19.6 ± 21.7	27.0 ± 25.6	26.2 ± 25.1	28.0 ± 24.5	28.2 ± 28.3	24.0 ± 26.5
Fruit intake, g/d	167 ± 124	205 ± 138	177 ± 117	171 ± 119	158 ± 128	124 ± 99.6	151 ± 137
Vegetable intake, g/d	150 ± 68.6	152 ± 63.5	149 ± 69.8	152 ± 72.1	148 ± 67.3	147 ± 70.5	146 ± 80.9
Fatty fish intake, g/d	13.3 ± 22.3	15.9 ± 22.5	15.1 ± 28.9	12.5 ± 17.6	12.2 ± 22.7	10.7 ± 17.7	13.9 ± 27.6
White fish intake, g/d	15.9 ± 17.6	15.1 ± 17.0	13.5 ± 15.0	16.7 ± 18.5	18.4 ± 21.0	15.7 ± 15.8	16.3 ± 22.3
Legumes intake, g/d	26.7 ± 26.9	26.7 ± 27.7	22.8 ± 23.4	26.4 ± 26.3	29.9 ± 31.4	27.6 ± 25.0	28.7 ± 29.8
Nuts intake, g/d	2.20 ± 5.66	2.36 ± 6.15	2.15 ± 4.84	2.22 ± 5.61	1.63 ± 4.51	2.62 ± 6.87	2.04 ± 7.25
Dairy intake, g/d	218 ± 142	220 ± 140	210 ± 146	215 ± 133	246 ± 135	197 ± 152	216 ± 159
Egg intake, g/d	14.0 ± 17.0	11.9 ± 17.6	12.2 ± 13.3	14.3 ± 17.4	13.9 ± 15.3	17.8 ± 20.3	15.3 ± 17.3
Sugar-sweetened beverage intake, g/d	30.9 ± 65.7	19.9 ± 51.6	31.2 ± 62.1	37.9 ± 74.6	29.3 ± 56.1	36.4 ± 78.8	45.1 ± 127

1 Values are mean ± SD for continuous variables and *n* (%) for categoric variables. PA, physical activity; Q, the red meat metabolite score in quintiles; T2D, type 2 diabetes.

**FIGURE 4:**
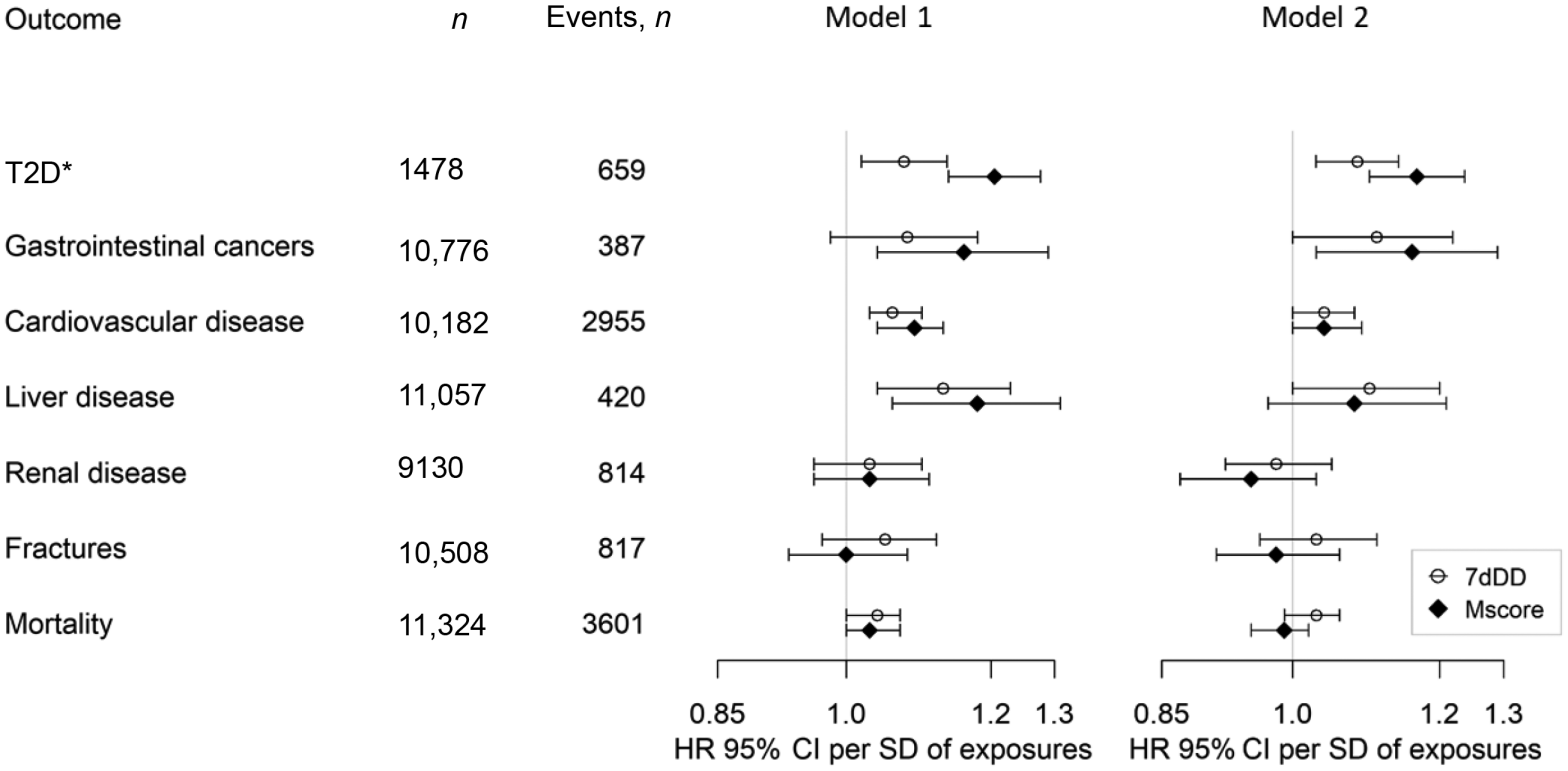
The associations of the red meat metabolite score and self-reported red meat intake with incident T2D in a nested case-cohort study and exploratory analyses of multiple other health outcomes in the EPIC-Norfolk study. Regression model 1 adjusted for age and sex; regression model 2 adjusted for the following potential confounders: age, sex, education, smoking status, alcohol drinking, alcohol drinking squared, BMI, BMI squared, and dietary factors (consumption of fruits, vegetables, fatty fish and white fish, sugar-sweetened beverages, dairy, legumes, nuts, eggs, and total energy intake). Supplemental Table 1 reports the definition of incident cases and exclusion of prevalent cases. *The association with incident T2D was conducted in a nested case-cohort study in the EPIC-Norfolk study; associations with other exploratory health outcomes were conducted in the EPIC-Norfolk study after exclusion of participants involved in the case-cohort study. EPIC-Norfolk, European Prospective Investigation into Cancer and Nutrition-Norfolk; Mscore, red meat metabolite score; T2D, type 2 diabetes; 7dDD, 7-d diet diary.

In a prospective analysis with a median follow-up of 10 y, the metabolite score for red meat consumption was positively associated with a higher risk of incident T2D (HR per SD: 1.17; 95% CI: 1.10, 1.24) after adjusting for potential confounding factors (**Figure 4**). There was a significant association between self-reported red meat consumption and incident T2D (HR per SD: 1.08; 95% CI: 1.03, 1.14).

### Association of the red meat metabolite score with other health outcomes

In an exploratory analysis, we examined the association of the red meat metabolite score with 6 health outcomes. In an adjusted analysis, a higher red meat metabolite score was significantly associated with higher risk of incident cardiovascular disease (HR per SD: 1.04; 95% CI: 1.00, 1.09) and gastrointestinal cancers (HR per SD: 1.16; 95% CI: 1.03, 1.29). The estimates of associations for meat intake using 7dDD measurements were similar to those using the derived scores but the *P* values were generally smaller (Figure 4).

## Discussion

In this article we report the development and validation of metabolite scores for 3 different types of meat consumption—red meat, processed meat, and poultry—based on untargeted plasma metabolomics data and 7dDD data in a large British cohort with comprehensive phenotypes. In focused analysis on the red meat metabolite score, we found that 11 top-ranked metabolites in the score were associated with red meat intake in an RCT, suggesting a causal link between red meat intake and change of these metabolites. Finally, we found that the red meat metabolite score was associated with T2D incidence and potentially also associated with other cardiometabolic diseases.

### Metabolite scores of meat consumption

Previous evidence on combining biomarkers into scores to measure meat intake is limited. A trial in Denmark indicated that combinations of several metabolic biomarkers of red meat intake were more efficient than a single biomarker in classifying red meat consumers compared with other participants (12). However, previous studies had not evaluated a dose–response association between meat intake and a combination of biomarkers. In this large population-based study, we estimated the absolute amounts of meat intake with 7dDDs, which provides more accurate estimates than a FFQ in ranking consumption amounts (27). Our results indicate the utility of untargeted metabolomics to generate an overall score to predict the amount of meat intake rather than only being able to discriminate between consumers and nonconsumers.

The metabolite scores of meat consumption were characterized by a wide range of metabolites, including lipids, amino acids, and xenobiotics. Several metabolites that constitute the derived scores have been identified by previous studies, such as TMAO, trans-4-hydroxyproline, creatine, and stearoylcarnitine (10, 11, 28). Specifically, an RCT in the United States (*n* = 113) reported that TMAO in plasma significantly increased after red meat consumption compared with consumption of poultry or nonmeat products. Positive associations of plasma TMAO concentrations with risk of cardiovascular disease, diabetes, and all-cause mortality have been reported in several meta-analyses of clinical studies (29–31). These results suggest that TMAO might be involved as part of underlying mechanisms between red meat intake and the development of chronic disease. In addition to metabolites in the score of red meat intake, several metabolites specific to processed meat (e.g., o-cresol sulphate) (32, 33) or poultry consumption (e.g., 3-methylhistidine) (10) in our study were also reported by previous intervention studies.

We also identified several yet-unreported metabolites that were associated with red meat consumption in both the observational study and the RCT, in particular several plasmalogens, such as 1-(1-enyl-stearoyl)-2-arachidonoyl-GPE (P-18:0/20:4), 1-margaroyl-2-oleoyl-GPC (17:0/18:1), and 1-palmityl-GPC (O-16:0). Plasmalogens, a subclass of membrane glycerophospholipids, contain a vinyl-ether bond at the sn-1 position and are enriched in PUFAs at the sn-2 position of the glycerol backbone (34). Mazzilli et al. (35) found several plasmalogens were correlated with self-reported red meat consumption. However, most of the plasmalogens identified in our study were not reported in that previous study, partly due to different platforms being used to measure and annotate metabolites in the different studies. These compounds present a very promising group of potential new biomarkers for meat intake. Their role in meat metabolism and disease development is largely unknown and warrants additional investigation. Some drug metabolites were also identified in the red meat metabolite score, such as Verapamil and Ranitidine. These metabolites were detected in only a small number of participants (**Supplemental Table 9**), so are likely to represent a subgroup of patients who have chronic disease and are taking these drugs (Verapamil for cardiac illness and Ranitidine for gastrointestinal illness). Because these conditions could themselves be linked to red meat consumption, it is likely that the association between these drug metabolites and dietary behavior is confounded by indication.

One group of metabolites which make major contributions to the red meat metabolite score are small meat-derived molecules with short half-lives (e.g., TMAO, trans-4 hydroxyproline, or creatine) (10). These compounds are unlikely to be good long-term markers of meat intake in people who consume meat occasionally because the metabolites would be cleared from the body relatively quickly. By contrast, these biomarkers may reflect regular red meat intake well. The second group of metabolites that rank highest in the score are lipophilic compounds (e.g., plasmalogens). These compounds have half-lives of days or even weeks and are likely to be good markers of long-term dietary habits (36, 37), and useful for identification of foods that are consumed rarely. These 2 groups of metabolites in the meat metabolite score ensure that the score reflects not only recent food intake but also dietary intake over a longer time frame. The focus on longer-term habitual intake as well as short-term intake is a strength of this study in respect of not only the biomarkers, but also the 7dDDs which have previously been shown to capture short-term and habitual dietary intakes (38).

### Associations with T2D risk

The red meat metabolite score, as a proxy for red meat intake, showed a positive association with T2D risk consistent with results from several large cohort studies that have reported associations of T2D risk with self-reported intake as dietary exposures (3, 4, 39, 40). The score-derived association appeared to be comparable in magnitude with that using 7dDD-measured meat intake. Similar results were reported in a study on a metabolomics signature of the Mediterranean diet and its association with cardiovascular disease risk (41). Future evaluations of the additional complementary information that can be obtained by measurement of metabolites over and above traditional dietary assessment methods should include investigation of cost-effectiveness and predictive utility for disease outcomes.

### Strengths and limitations

To our knowledge, this study was the first of this kind to develop and validate a metabolite score for red meat intake in a large population study which has comprehensive dietary measurements and metabolomics data. Metabolite profiling provided a complementary approach to assess types of meat consumption objectively. The application of metabolomics to a meat intervention trial provided additional evidence on biological plausibility and reproducibility of the red meat metabolite score. In addition, in the EPIC-Norfolk study, a long follow-up with detailed information on multiple incident diseases enabled us to examine associations between the meat metabolite score and multiple health outcomes simultaneously.

Several limitations warrant discussion. Firstly, the study was based on a British population so generalizability is limited for other populations and further validation studies should be considered. Secondly, although we have adjusted for a comprehensive set of confounders to examine the association between the red meat metabolite score and risks of noncommunicable diseases, the results may be affected by residual confounding. Thirdly, although we have tested the change of metabolites after meat intervention in a trial, the limited number of red meat products and the limited size of the trial hindered a comprehensive validation analysis. The potential causal links between meat intake and most of the candidate metabolites are largely unknown. Many metabolites in the score are probably not directly influenced by meat intake, but are affected by factors that are correlated with meat intake, such as BMI, or derived from metabolic or physiologic processes. Also, we might be unable to validate metabolites that reflect long-term diets because the feeding study tested short-term exposures. However, the most important metabolites were validated in the RCT and the score correlated well with meat intake in the validation set. Further validation studies with a wider range of confirmed metabolites in other populations are needed.

### Conclusion

This study suggests that a metabolite score derived from untargeted metabolomics profiles in plasma has the potential to reflect red meat consumption and inform the study of the association of red meat consumption, assessed objectively, with clinical outcomes.

## Supplementary Material

nqac094_Supplemental_File

## Data Availability

The data described in the article and analytic code will be made available upon request, pending application and approval. The metadata for the EPIC-Norfolk study including the study dictionary are freely available without restriction at https://www.epic-norfolk.org.uk/.

## Notes

The European Prospective Investigation into Cancer and Nutrition-Norfolk (EPIC-Norfolk) study (https://doi.org/10.22025/2019.10.105.00004) has received funding from Medical Research Council (MRC) grants MR/N003284/1, MC_UU_12015/1, and MC_UU_00006/1 and Cancer Research UK grant C864/A14136. Metabolite measurements in the EPIC-Norfolk study were supported by MRC Cambridge Initiative in Metabolic Science grant MR/L00002/1 and the Innovative Medicines Initiative Joint Undertaking under European Medical Information Framework grant agreement no. 115372. NJW, NGF, FI, IDS, MP, EW, and C Langenberg were supported by MRC Epidemiology Unit grants MC_UU_00006/1 and MC_UU_00006/3. NJW and NGF were supported by National Institute for Health and Care Research Cambridge Biomedical Research Centre: nutrition, diet, and lifestyle research theme grant IS-BRC-1215-20014. C Li was supported by a Jardine-Cambridge Graduate Scholarship. For the purpose of open access, the author has applied a Creative Commons Attribution (CC BY) licence to any Author Accepted Manuscript version arising.

FI is a member of the ASN Statistical Review Board.

Supplemental Methods, Supplemental Figures 1–5, and Supplemental Tables 1–9 are available from the “Supplementary data” link in the online posting of the article and from the same link in the online table of contents at https://academic.oup.com/ajcn/.

Abbreviations used: eGFR, estimated glomerular filtration rate; EPIC-Norfolk, European Prospective Investigation into Cancer and Nutrition-Norfolk; GPC, glycerophosphocholine; GPE, glycerophosphoethanolamine; IARC, International Agency for Research on Cancer; RCT, randomized controlled trial; TMAO, trimethylamine N-oxide; T2D, type 2 diabetes; 7dDD, 7-d diet diary.
