# Supplementary material for: Development and validation of a metabolite score for red meat intake: an observational cohort study and randomized controlled dietary intervention
Source: Am J Clin Nutr. 2022 Jun 27;116(2):511–22. doi: 10.1093/ajcn/nqac094 (PMC9348983; doi:10.1093/ajcn/nqac094)
Supplement: nqac094_Supplemental_File [file nqac094_supplemental_file.pdf]

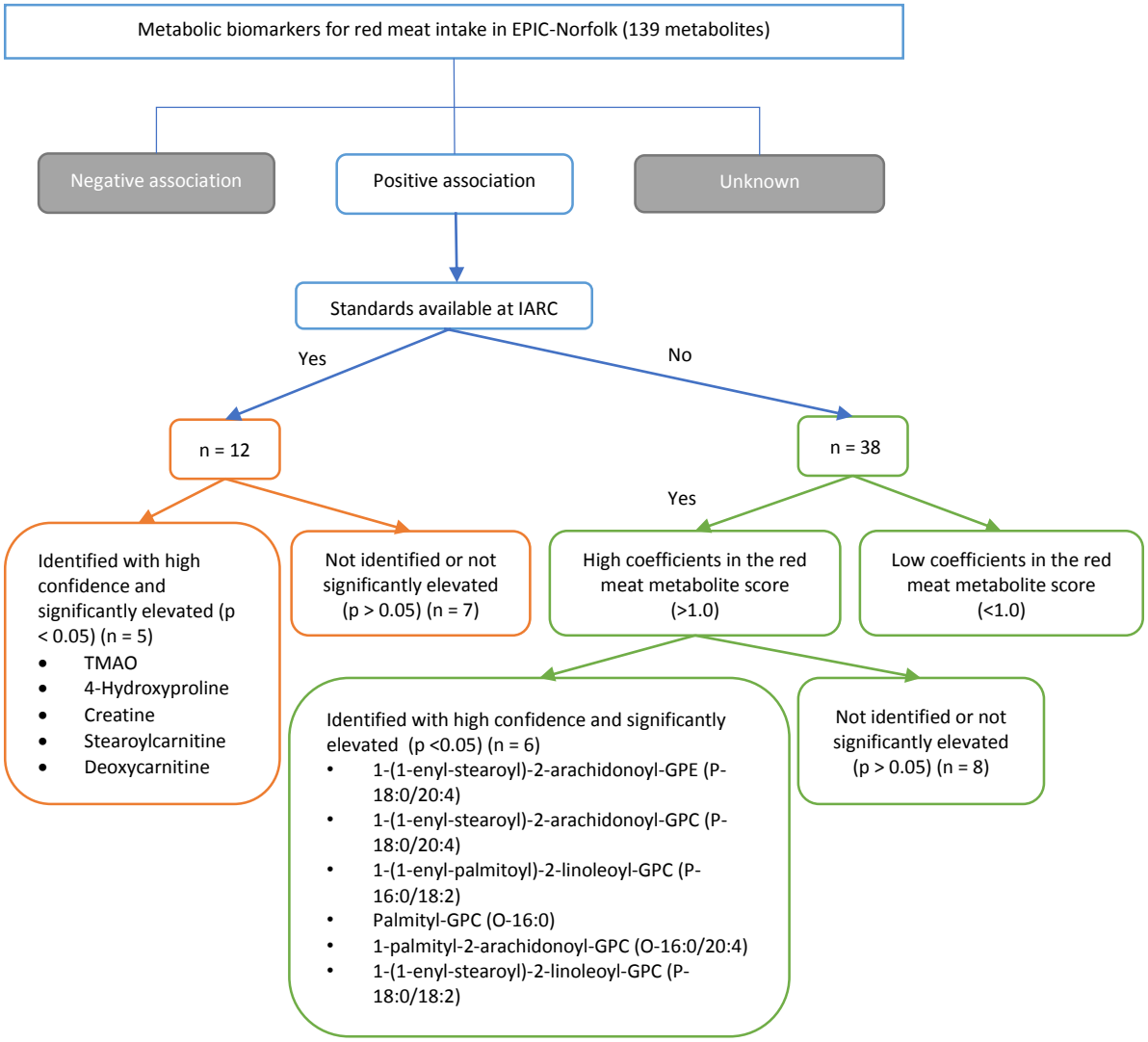


**Supplementary Figure 1.** Flowchart for identification of metabolites that make up the red meat metabolite score in the trial. GPE, glycerophosphoethanolamine; GPC, glycerophosphocholine; IARC, International Agency for Research on Cancer; TMAO, trimethylamine N-oxide.

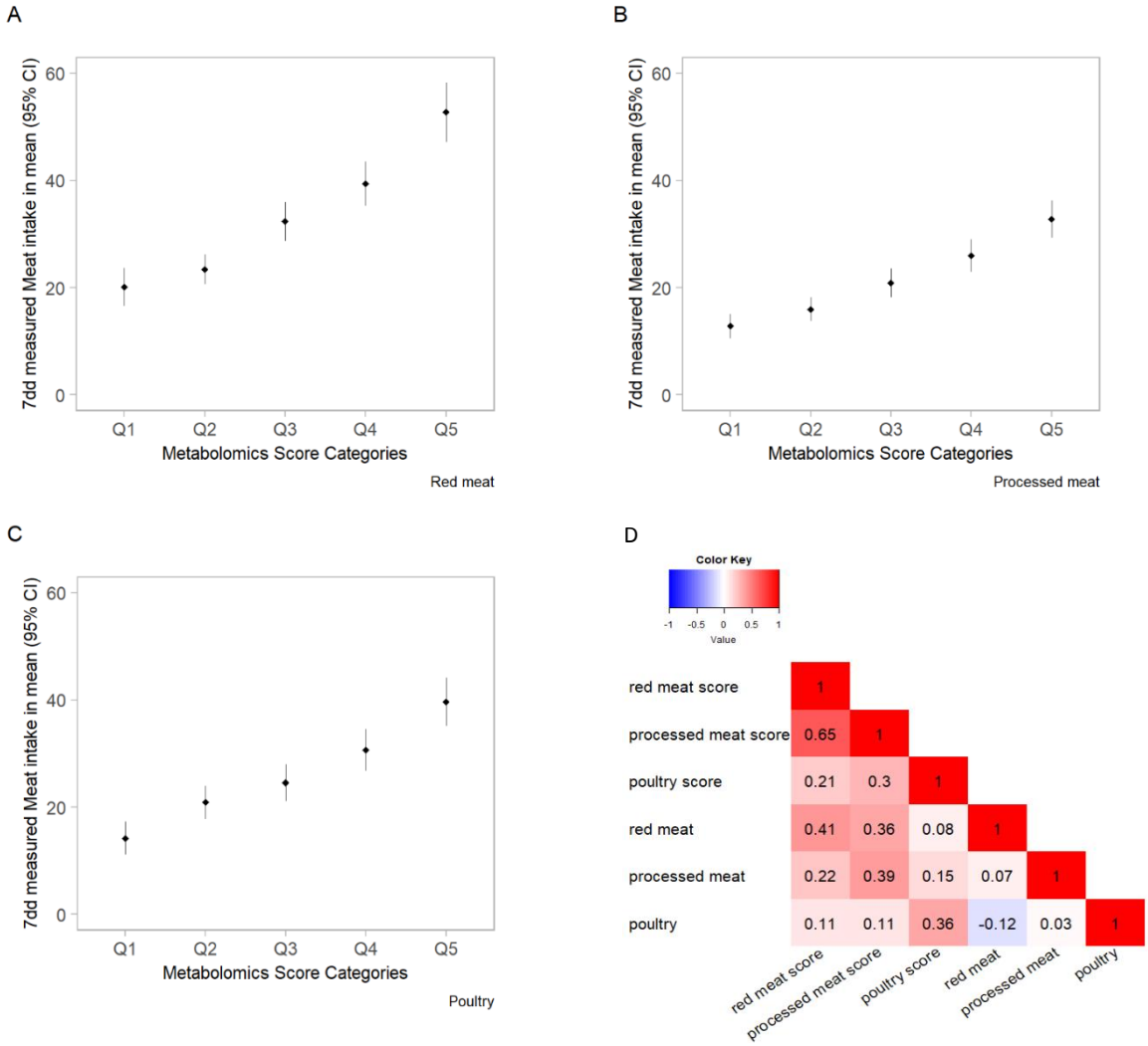


**Supplementary Figure 2.** The correlations between meat scores and 7day diet diary (7dDD) measured meat intake. A, the means and 95% confidence intervals (CI) of red meat consumption measured by (7dDD) in quintiles of the derived red meat metabolite score (139 metabolites) in the exploratory set in the EPIC-Norfolk study (n=11,432); B, the means and 95% CI of processed meat consumption measured by 7dDD in quintiles of the processed meat metabolite score (82 metabolites) in the exploratory set in the EPIC-Norfolk study (n=11,432); C, the means and 95% CI of poultry consumption measured by 7dDD in quintiles of the poultry metabolite score (139 metabolites) in the exploratory set in the EPIC-Norfolk study (n=11,432); D. the correlations matrix for consumption of types of meat (red meat, processed meat and poultry) measured by 7dDD and measured by derived metabolite scores in the validation set in the EPIC-Norfolk study (n=853).

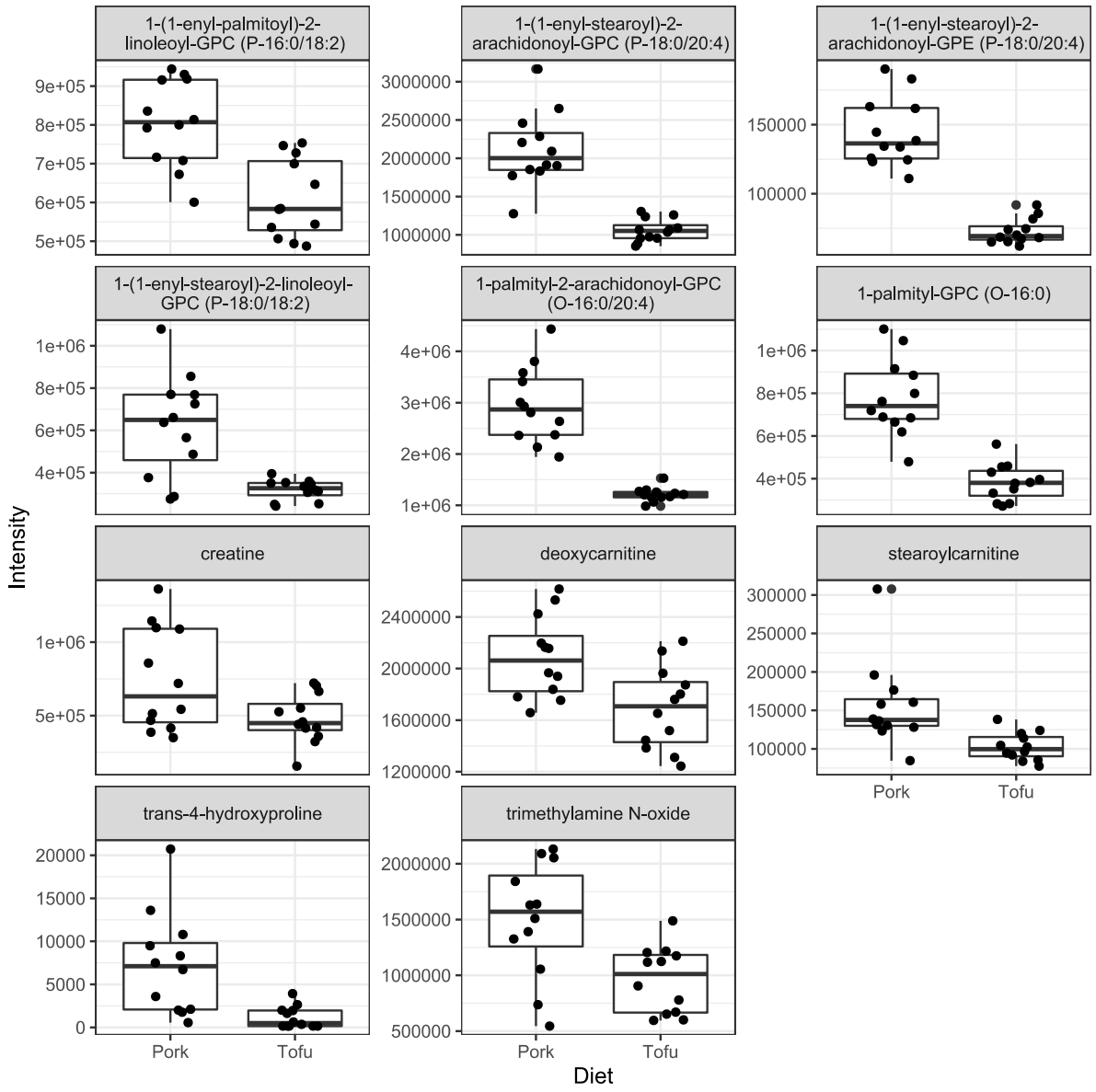


**Supplementary Figure 3.** Plasma levels of selected metabolites after consumption of pork and tofu in the randomized cross-over trial. Metabolites that were positively associated with red meat consumption in both the EPIC-Norfolk and the randomized cross-over trial are shown. Fold-change and p-values are reported in Table 2.

| Compound | Chromatogram in plasma after tofu intake | Chromatogram in plasma after pork intake | Isotope pattern | Chromatograms in several plasma samples after pork intake |
|---|---|---|---|---|
| Deoxycarnitine | 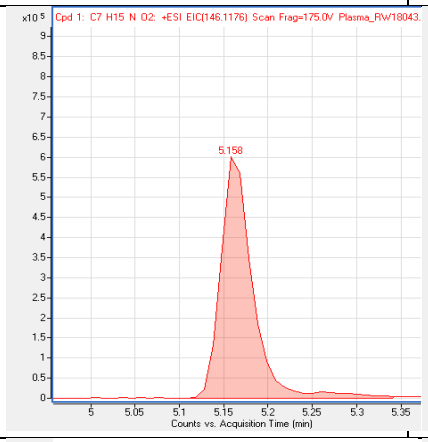 | 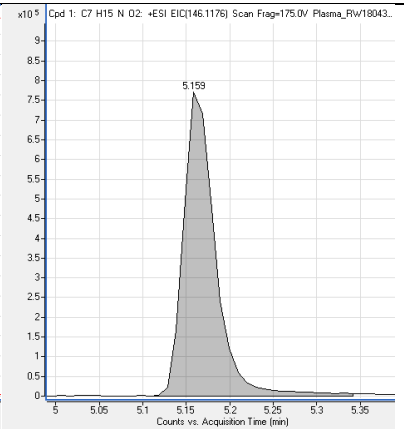 | 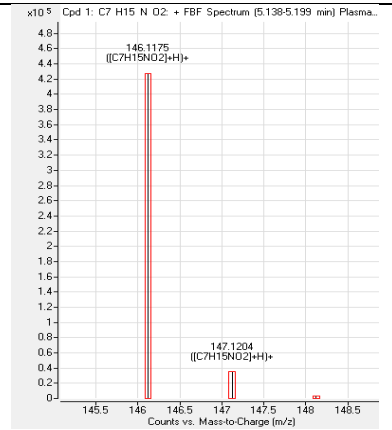 | 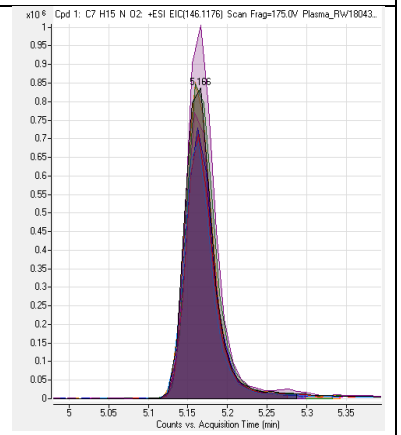 |
| 1-(1-enyl-stearoyl)-2-arachidonoyl-GPE (P-18:0/20:4) | 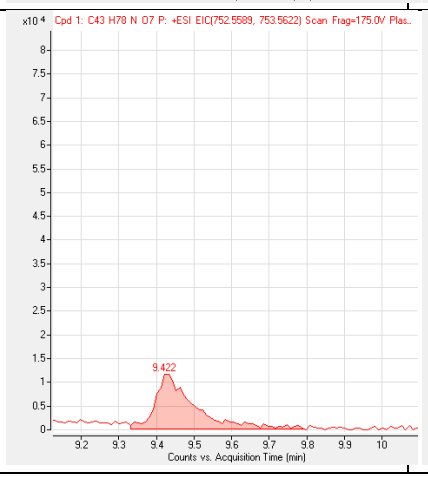 | 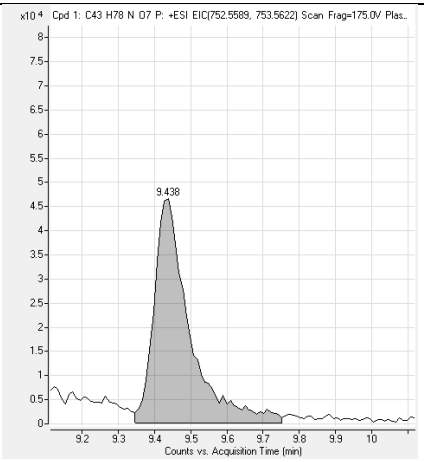 | 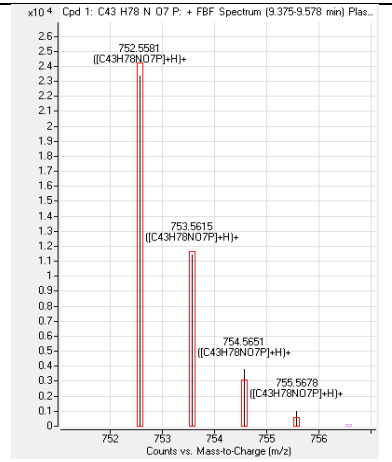 | 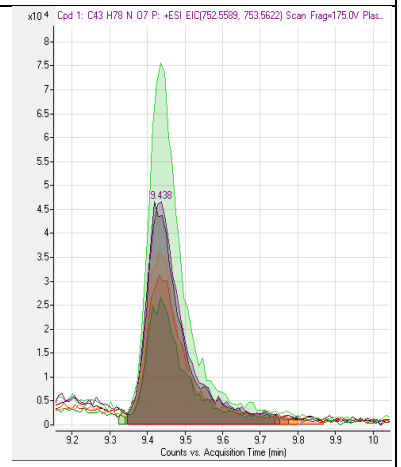 |

| | | | | |
|---|---|---|---|---|
| 1-(1-enyl-stearoyl)-2-linoleoyl-GPC (P-18:0/18:2) | 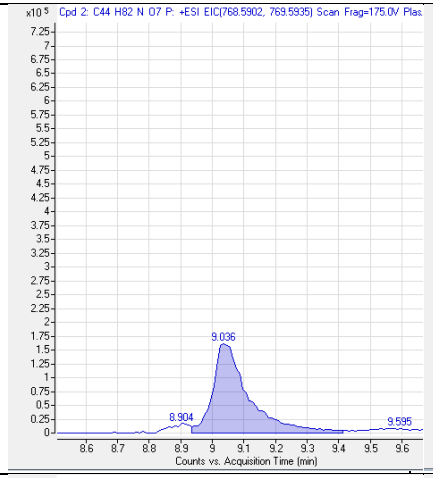 | 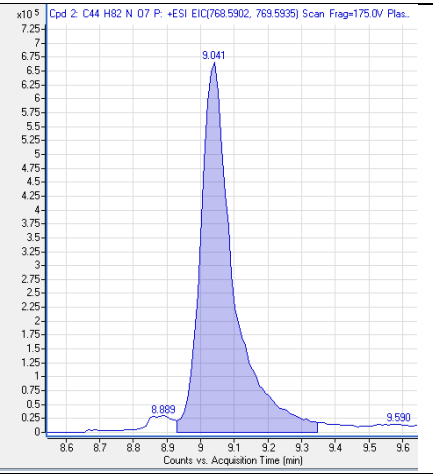 | 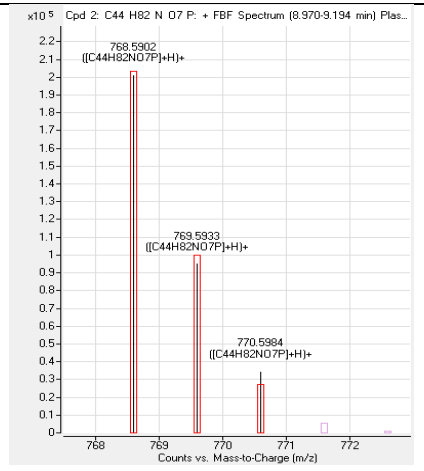 | 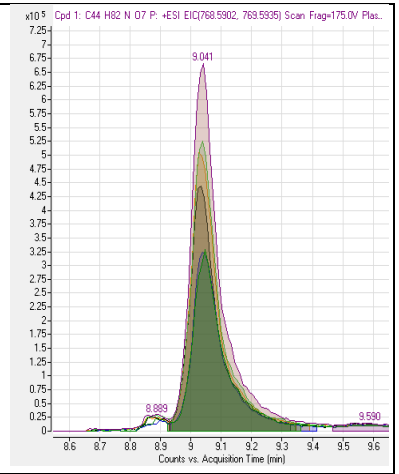 |
| Stearoylcarnitine | 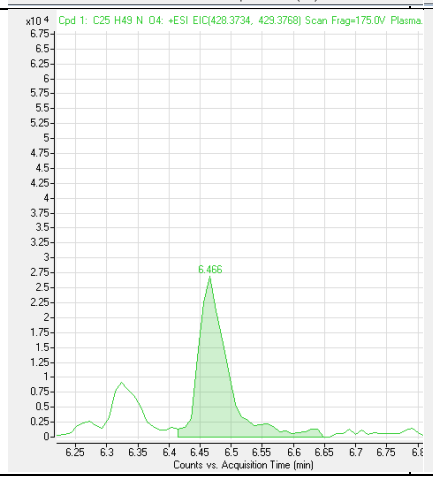 | 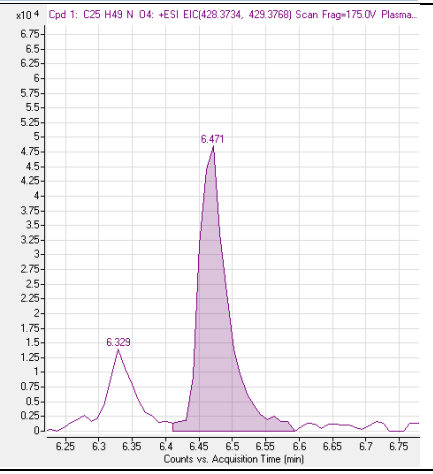 | 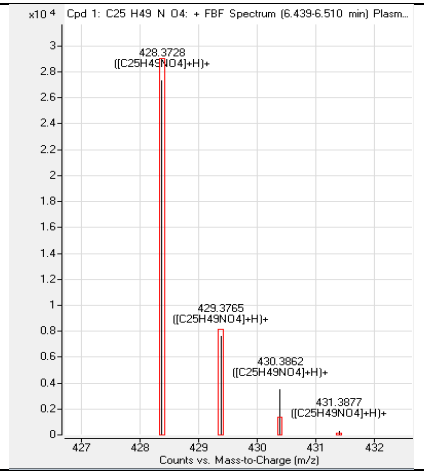 | 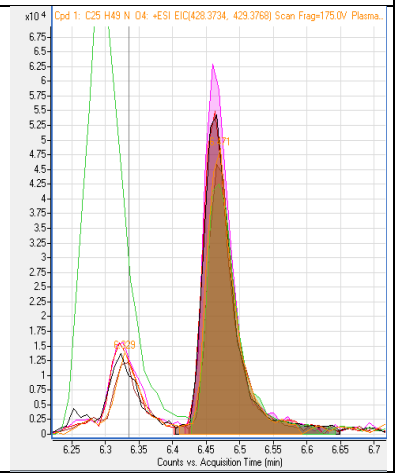 |

**Supplementary Figure 4.** Chromatographic tracing of selected metabolites after consumption of pork vs. tofu in the intervention study. Column 2 and 3 show the chromatogram of a compound after tofu intake and pork intake separately in the same participant. Isotope pattern was used as one indicator of the peak quality. The vertical lines represent the detected intensities of compounds. The boxes show the expected peaks. The plots indicate that high intensity compounds usually match very well with the expected isotope pattern. Column 5 shows the chromatogram of a compound in several samples of plasma after pork intake. It shows the variability of peak shapes and intensities (the variation of intensity of metabolites is reported in the boxplots in Supplementary Figure 3)

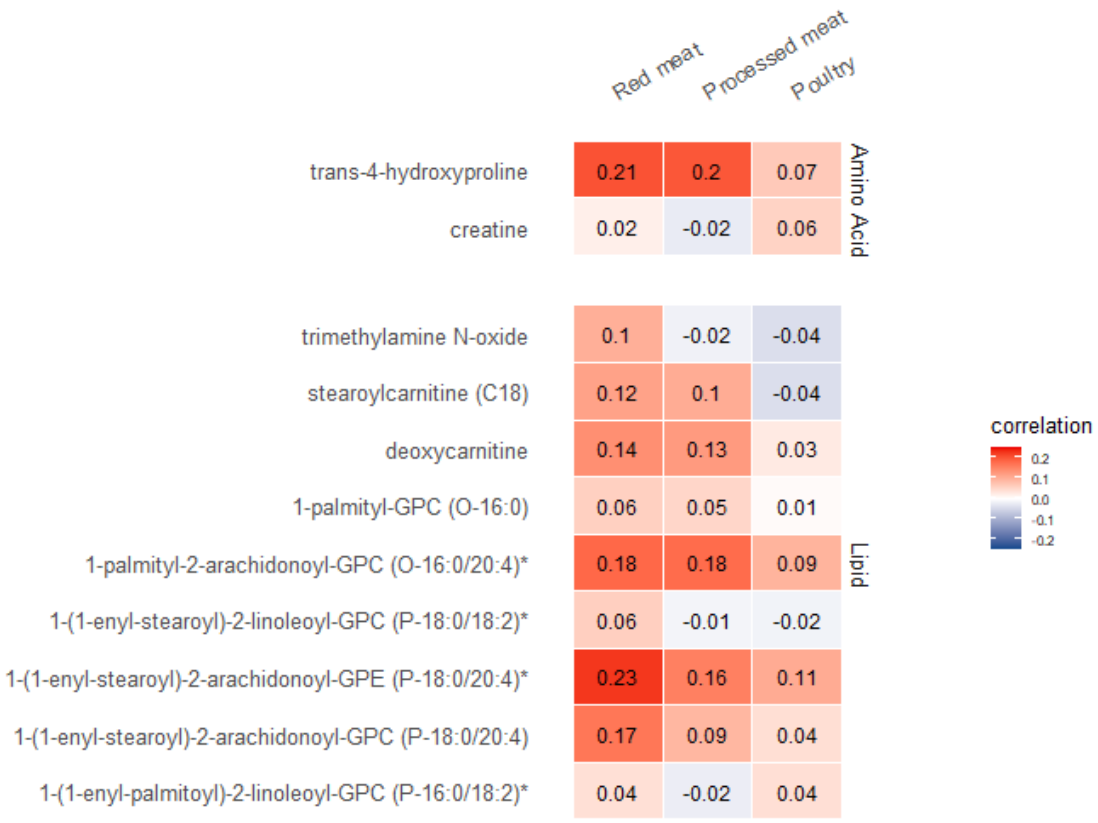


**Supplementary Figure 5.** Heatmap of correlations between types of meat consumption and top-ranked metabolites (n=11) in the red meat metabolite score that validated in the intervention study: EPIC-Norfolk study (n=11,432). The single asterisk in metabolite name represents the metabolite was annotated based on in-silico predictions which indicates the compound has not been confirmed based on a standard but its identity is confident.

Supplemental File for publication

Click here to access/download;Supplemental File for publication;OSM_supplementary tables 1-9_FINAL.xlsx

Development and validation of a metabolite score for red meat intake: an observational cohort study an

**The table of contents for supplementary tables.**

**Supplementary Table 1.** The definition of non-communicable diseases outcomes in the exploratory analy
**Supplementary Table 2.** Overview of metabolites included for the red meat metabolite score. A single as
**Supplementary Table 3**. Overview of metabolites included for the processed meat metabolite score. A si
**Supplementary Table 4.** Overview of metabolites included for the poultry metabolite score.
**Supplementary Table 5.** The parameters of feature (metabolite) selection using bootstrapping enhanced e
**Supplementary Table 6.** The rank of metabolites in the red meat metabolite score ordered by selected tim
**Supplementary Table 7.** The number of metabolites in each metabolite score and its explained variance o
**Supplementary Table 8.** Correlation matrix of top-ranked metabolites in the red meat metabolite score an
**Supplementary Table 9.** The missing rates of metabolites in the red meat metabolite score in the explorat

d randomized controlled dietary intervention. Chunxiao Li Online Supplementary Material

ʏses for the association between red meat metabolite score and health outcomes.
terisk in metabolite name represents the metabolite was annotated based on in-silico predictions which indi
ngle asterisk in metabolite name represents the metabolite was annotated based on in-silico predictions whi

elastic net regression.
ues in the bootstrapping process.
of meat consumption using different thresholds in the exploratory and validation sets.
ıd identified in the RCT (n=11).
ory set (combination of batch 2 and batch 3) and subgroups of red meat consumers and non-consumers.

cates the compound has not been confirmed based on a standard but its identity is confident.
ch indicates the compound has not been confirmed based on a standard but its identity is confident.

**Supplementary Table 1. The definition of non-communicable diseases outcomes in the ex**

| | Name | Definition | notes |
|---|---|---|---|
| Incident cardiovascular disease | Incident coronary heart disease | ICD-9 codes: 410-414; ICD-10 codes: I20-I25 | Incident cases were defined either by hospital admissions data or death certificate. |
| | Incident cerebral stroke | ICD-9 codes: 433-435; ICD-10 codes: I63, I65, I66 | Incident cases were defined either by hospital admissions data or death certificate. |
| | Incident haemorrhagic stroke | ICD-9 codes: 430-432; ICD-10 codes: I60-I62 | Incident cases were defined either by hospital admissions data or death certificate. |
| | Incident atrial fibrillation | ICD-9 codes: 427.3; ICD-10 codes: I48 | Incident cases were defined either by hospital admissions data or death certificate. |
| | Incident heart failure | ICD-9 codes: 428; ICD-10 codes: I50 | Incident cases were defined either by hospital admissions data or death certificate. |
| Incident liver disease | Incident liver disease | ICD-10 codes: B15-19, C22, E83, E88, I85, K70, K72-76, R18, Z94 | Incident cases were defined either by hospital admissions data or death certificate. |
| Incident renal disease | Incident renal disease | ICD-9 codes: 580-589, 593; ICD-10 codes: N00-N19, N25-N29 | Incident cases were defined either by hospital admissions data or death certificate. |
| Incident gastrointestinal cancer | Incident colon cancer | ICD-9 codes: 153.0-153.9; ICD-10 codes: C18 | Incident cases were defined either by hospital admissions data or death certificate. |
| | Incident rectal cancer | ICD-9 codes: 154.0-154.1, 159.0; ICD-10 codes: C19-C20 | Incident cases were defined either by hospital admissions data or death certificate. |

| | | | |
|---|---|---|---|
| | Incident stomach cancer | ICD-9 codes: 151; ICD-10 codes: C16 | Incident cases were defined either by hospital admissions data or death certificate. |
| Incident fra | Incident fractures | ICD-10 codes: S02, S12, S22, S32, S42, S52, S62, S72, S82, S92, S120-S122, S127-S129, S220-S225, S228, S229, S320-SS325, S327, S328, S520-S529, S620-S627, S720-S729, S820-S829, S920-S929, T02, T08, T10 | Incident cases were defined based on hospital admission data. |
| All-cause mortality | All-cause mortality | | Mortality from all causes was defined from death certificates. |

| ploratory analyses for the associatio |
| --- |
| **prevalent cases for exclusion** |
| Prevalent coronary heart disease was defined by a self-reported history of either angina or myocardial infarction. |
| Prevalent stroke was defined based on a self-reported history of stroke (any kind) by a doctor. |
| Prevalent stroke was defined based on a self-reported history of stroke (any kind) by a doctor. |
| We defined prevalent atrial fibrillation (AF) by self-reported intake of drugs that were used for treatment of AF in clinical practice at the time of the baseline survey (digitalis or vitamin K antagonists; PMID 25059930). |
| We defined prevalent heart failure by self-reported intake of drugs that were recommended for treatment of heart failure, namely loop diuretics in combination with digitalis or angiotensin-converting enzyme inhibitors (PMID 21835284). |
| Prevalent liver disease was defined based on self-reported diagnosis of any liver disease by a doctor. |
| Prevalent kidney disease was defined as an eGFR < 50 ml/min/1.73m$^2$. |
| Prevalent cases were defined based on a self-reported history of any cancer. |
| Prevalent cases were defined based on a self-reported history of any cancer. |

| |
|---|
| Prevalent cases were defined based on a self-reported history of any cancer. |
| Prevalent cases were reported based on any reported fracture at baseline examinations. |
| |

**Supplementary Table 2. Overview of metabolites included for the re**

| BIOCHEMICAL |
|---|
| 1-(1-enyl-stearoyl)-2-arachidonoyl-GPE (P-18:0/20:4)* |
| 1-(1-enyl-stearoyl)-2-arachidonoyl-GPC (P-18:0/20:4) |
| 1-margaroyl-2-oleoyl-GPC (17:0/18:1)* |
| trans-4-hydroxyproline |
| verapamil |
| X - 11381 |
| trimethylamine N-oxide |
| 1-(1-enyl-palmitoyl)-2-linoleoyl-GPC (P-16:0/18:2)* |
| 1-palmityl-GPC (O-16:0) |
| 1-palmitoyl-2-palmitoleoyl-GPC (16:0/16:1)* |
| creatinine |
| 17-methylstearate |
| creatine |
| ergothioneine |
| 10-heptadecenoate (17:1n7) |
| sphingomyelin (d18:1/15:0, d16:1/17:0)* |
| 1-palmityl-2-arachidonoyl-GPC (O-16:0/20:4)* |
| 1-(1-enyl-stearoyl)-2-linoleoyl-GPC (P-18:0/18:2)* |
| methionine sulfone |
| 1-docosapentaenoyl-GPC (22:5n3)* |
| deoxycarnitine |
| 3-carboxy-4-methyl-5-propyl-2-furanpropanoate (CMPF) |
| cholesterol |
| X - 02249 |
| N-acetylphenylalanine |
| imidazole lactate |
| 2-hydroxyoctanoate |
| N-acetylcarnosine |
| 1-stearoyl-2-oleoyl-GPE (18:0/18:1) |
| choline |
| ranitidine |
| X - 21315 |
| X - 12731 |
| X - 18913 |
| 5-methyluridine (ribothymidine) |
| 1-oleoyl-GPI (18:1)* |
| alpha-hydroxyisocaproate |
| 4-vinylphenol sulfate |
| 5alpha-androstan-3alpha,17beta-diol monosulfate (2) |
| N-palmitoyl-sphingosine (d18:1/16:0) |
| X - 24309 |
| gamma-glutamylvaline |
| andro steroid monosulfate (1)* |
| N-acetylglutamine |
| dodecanedioate |
| S-methylcysteine |
| X - 11483 |
| X - 24293 |

1-myristoylglycerol (14:0)
X - 21659
2-hydroxy-3-methylvalerate
X - 16580
X - 21442
fructose
sphingosine
N-acetylputrescine
stearoylcarnitine
cysteine sulfinic acid
X - 15492
X - 18779
X - 14662
4-vinylguaiacol sulfate
X - 23583
serotonin
N-acetyl-1-methylhistidine*
X - 17145
phenylacetate
X - 11852
guanosine
pantothete
propionylglycine
X - 21821
4-hydroxyhippurate
X - 12729
X - 13658
X - 11315
vanillic alcohol sulfate
X - 13729
X - 23782
X - 23593
methionine sulfoxide
carotene diol (2)
X - 12212
13-HODE + 9-HODE
N-acetylaspartate (A)
taurocholate
homostachydrine*
cysteine
5-hydroxylysine
1-palmityl-2-palmitoyl-GPC (O-16:0/16:0)*
X - 11849
X - 21286
2-linoleoylglycerol (18:2)
phenol sulfate
glycerate
1-arachidonoyl-GPA (20:4)
1-(1-enyl-palmitoyl)-2-myristoyl-GPC (P-16:0/14:0)*
X - 11905

propyl 4-hydroxybenzoate sulfate
X - 18914
X - 17676
furosemide
X - 18899
quinine
1-(1-enyl-palmitoyl)-2-docosahexaenoyl-GPE (P-16:0/22:6)*
2-oxoarginine*
uridine
alanine
O-sulfo-L-tyrosine
1-methylimidazoleacetate
asparagine
4-acetylphenol sulfate
2-palmitoyl-GPC (16:0)*
3-aminoisobutyrate
4-hydroxyphenylpyruvate
1-(1-enyl-palmitoyl)-2-palmitoyl-GPC (P-16:0/16:0)*
betaine
1-stearoyl-2-arachidonoyl-GPI (18:0/20:4)
1-(1-enyl-stearoyl)-2-docosahexaenoyl-GPE (P-18:0/22:6)*
sphingomyelin (d18:1/20:1, d18:2/20:0)*
pseudouridine
1-(1-enyl-palmitoyl)-2-oleoyl-GPC (P-16:0/18:1)*
citrulline
pyroglutamine*
X - 13684
X - 09789
X - 14838
1-arachidonoyl-GPC (20:4n6)*
palmitoyl dihydrosphingomyelin (d18:0/16:0)*
X - 12511
docosahexaenoate (DHA; 22:6n3)
1-stearoyl-2-arachidonoyl-GPC (18:0/20:4)
glycine
sphingomyelin (d18:2/14:0, d18:1/14:1)*
1-pentadecanoyl-GPC (15:0)*
1-palmitoyl-2-linoleoyl-GPC (16:0/18:2)
X - 12442
triamterene
sphingomyelin (d18:1/14:0, d16:1/16:0)*

**d meat metabolite score**

| Sub_pathway | Super_pathway |
|---|---|
| Plasmalogen | Lipid |
| Plasmalogen | Lipid |
| Phosphatidylcholine (PC) | Lipid |
| Urea cycle; Arginine and Proline Metabolism | Amino Acid |
| Drug | Xenobiotics |
| Unknown | Unknown |
| Phospholipid Metabolism | Lipid |
| Plasmalogen | Lipid |
| Lysoplasmalogen | Lipid |
| Phosphatidylcholine (PC) | Lipid |
| Creatine Metabolism | Amino Acid |
| Fatty Acid, Branched | Lipid |
| Creatine Metabolism | Amino Acid |
| Food Component/Plant | Xenobiotics |
| Long Chain Fatty Acid | Lipid |
| Sphingolipid Metabolism | Lipid |
| Plasmalogen | Lipid |
| Plasmalogen | Lipid |
| Methionine, Cysteine, SAM and Taurine Metabolism | Amino Acid |
| Lysophospholipid | Lipid |
| Carnitine Metabolism | Lipid |
| Fatty Acid, Dicarboxylate | Lipid |
| Sterol | Lipid |
| Unknown | Unknown |
| Phenylalanine Metabolism | Amino Acid |
| Histidine Metabolism | Amino Acid |
| Fatty Acid, Monohydroxy | Lipid |
| Dipeptide Derivative | Peptide |
| Phosphatidylethanolamine (PE) | Lipid |
| Phospholipid Metabolism | Lipid |
| Drug | Xenobiotics |
| Unknown | Unknown |
| Unknown | Unknown |
| Unknown | Unknown |
| Pyrimidine Metabolism, Uracil containing | Nucleotide |
| Lysophospholipid | Lipid |
| Leucine, Isoleucine and Valine Metabolism | Amino Acid |
| Benzoate Metabolism | Xenobiotics |
| Androgenic Steroids | Lipid |
| Ceramides | Lipid |
| Unknown | Unknown |
| Gamma-glutamyl Amino Acid | Peptide |
| Androgenic Steroids | Lipid |
| Glutamate Metabolism | Amino Acid |
| Fatty Acid, Dicarboxylate | Lipid |
| Methionine, Cysteine, SAM and Taurine Metabolism | Amino Acid |
| Unknown | Unknown |
| Unknown | Unknown |

| | |
|---|---|
| Monoacylglycerol | Lipid |
| Unknown | Unknown |
| Leucine, Isoleucine and Valine Metabolism | Amino Acid |
| Unknown | Unknown |
| Unknown | Unknown |
| Fructose, Mannose and Galactose Metabolism | Carbohydrate |
| Sphingolipid Metabolism | Lipid |
| Polyamine Metabolism | Amino Acid |
| Fatty Acid Metabolism(Acyl Carnitine) | Lipid |
| Methionine, Cysteine, SAM and Taurine Metabolism | Amino Acid |
| Unknown | Unknown |
| Unknown | Unknown |
| Unknown | Unknown |
| Food Component/Plant | Xenobiotics |
| Unknown | Unknown |
| Tryptophan Metabolism | Amino Acid |
| Histidine Metabolism | Amino Acid |
| Unknown | Unknown |
| Phenylalanine Metabolism | Amino Acid |
| Unknown | Unknown |
| Purine Metabolism, Guanine containing | Nucleotide |
| Pantothete and CoA Metabolism | Cofactors and Vitamins |
| Fatty Acid Metabolism (also BCAA Metabolism) | Lipid |
| Unknown | Unknown |
| Benzoate Metabolism | Xenobiotics |
| Unknown | Unknown |
| Unknown | Unknown |
| Unknown | Unknown |
| Tyrosine Metabolism | Amino Acid |
| Unknown | Unknown |
| Unknown | Unknown |
| Unknown | Unknown |
| Methionine, Cysteine, SAM and Taurine Metabolism | Amino Acid |
| Food Component/Plant | Xenobiotics |
| Unknown | Unknown |
| Fatty Acid, Monohydroxy | Lipid |
| Alanine and Aspartate Metabolism | Amino Acid |
| Primary Bile Acid Metabolism | Lipid |
| Food Component/Plant | Xenobiotics |
| Methionine, Cysteine, SAM and Taurine Metabolism | Amino Acid |
| Lysine Metabolism | Amino Acid |
| Plasmalogen | Lipid |
| Unknown | Unknown |
| Unknown | Unknown |
| Monoacylglycerol | Lipid |
| Tyrosine Metabolism | Amino Acid |
| Glycolysis, Gluconeogenesis, and Pyruvate Metabolism | Carbohydrate |
| Lysophospholipid | Lipid |
| Plasmalogen | Lipid |
| Unknown | Unknown |

| | |
|---|---|
| Benzoate Metabolism | Xenobiotics |
| Unknown | Unknown |
| Unknown | Unknown |
| Drug | Xenobiotics |
| Unknown | Unknown |
| Drug | Xenobiotics |
| Plasmalogen | Lipid |
| Urea cycle; Arginine and Proline Metabolism | Amino Acid |
| Pyrimidine Metabolism, Uracil containing | Nucleotide |
| Alanine and Aspartate Metabolism | Amino Acid |
| Chemical | Xenobiotics |
| Histidine Metabolism | Amino Acid |
| Alanine and Aspartate Metabolism | Amino Acid |
| Drug | Xenobiotics |
| Lysophospholipid | Lipid |
| Pyrimidine Metabolism, Thymine containing | Nucleotide |
| Tyrosine Metabolism | Amino Acid |
| Plasmalogen | Lipid |
| Glycine, Serine and Threonine Metabolism | Amino Acid |
| Phosphatidylinositol (PI) | Lipid |
| Plasmalogen | Lipid |
| Sphingolipid Metabolism | Lipid |
| Pyrimidine Metabolism, Uracil containing | Nucleotide |
| Plasmalogen | Lipid |
| Urea cycle; Arginine and Proline Metabolism | Amino Acid |
| Glutamate Metabolism | Amino Acid |
| Unknown | Unknown |
| Unknown | Unknown |
| Unknown | Unknown |
| Lysophospholipid | Lipid |
| Sphingolipid Metabolism | Lipid |
| Unknown | Unknown |
| Polyunsaturated Fatty Acid (n3 and n6) | Lipid |
| Phosphatidylcholine (PC) | Lipid |
| Glycine, Serine and Threonine Metabolism | Amino Acid |
| Sphingolipid Metabolism | Lipid |
| Lysophospholipid | Lipid |
| Phosphatidylcholine (PC) | Lipid |
| Unknown | Unknown |
| Drug | Xenobiotics |
| Sphingolipid Metabolism | Lipid |

| PLATFORM | Retention Index | MASS | PubChem | ChemSpider | HMBD_ID |
|---|---|---|---|---|---|
| LC/MS Pos Late | 2511 | 752.5589 | 9547058 | 7826008 | HMDB05779 |
| LC/MS Pos Late | 2350 | 794.6058 | | 24767528 | |
| LC/MS Pos Late | 2450 | 774.6007 | | 24822423 | |
| LC/MS Pos Early | 1064 | 132.0655 | 5810 | 5605 | HMDB00725 |
| LC/MS Pos Late | 945 | 455.2904 | 2520 | 2425 | HMDB01850 |
| LC/MS Neg | 1118 | 184.0982 | | | |
| LC/MS Pos Early | 2100 | 76.0757 | 1145 | 1113 | HMDB00925 |
| LC/MS Pos Late | 2226 | 742.5745 | | 24767486 | HMDB11211 |
| LC/MS Pos Late | 1573 | 482.3605 | 3983 | 3845 | |
| LC/MS Pos Late | 2160 | 732.5538 | | | HMDB07969 |
| LC/MS Pos Early | 2055 | 114.0662 | 588 | 568 | HMDB00562 |
| LC/MS Neg | 5993 | 297.2799 | 3083779 | 2340933 | HMDB37397 |
| LC/MS Pos Early | 1947 | 132.0768 | 586 | 566 | HMDB00064 |
| LC/MS Pos Early | 850 | 230.0958 | 3032311 | 2297320 | HMDB03045 |
| LC/MS Neg | 5555 | 267.233 | 5312435 | 4471860 | HMDB60038 |
| LC/MS Pos Late | 2082 | 689.5592 | | 28532779 | |
| LC/MS Pos Late | 2183 | 768.5902 | 6443139 | 4947173 | |
| LC/MS Pos Late | 2461 | 770.6058 | | 24767519 | |
| LC/MS Pos Early | 1250 | 182.0482 | 69961 | 63154 | |
| LC/MS Neg | 5958 | 644.3569 | | | |
| LC/MS Pos Early | 2052 | 146.1176 | 134 | 705 | HMDB01161 |
| LC/MS Neg | 2840 | 239.0925 | 123979 | 110498 | HMDB61112 |
| LC/MS Pos Late | 2707 | 369.3516 | 11025495 | 4937803 | HMDB00067 |
| LC/MS Neg | 4012 | 267.1239 | | | |
| LC/MS Neg | 2597 | 206.0823 | 74839 | 67404 | HMDB00512 |
| LC/MS Pos Early | 2040 | 157.0608 | 440129 | 389128 | HMDB02320 |
| LC/MS Neg | 3736.8 | 159.1027 | 94180 | 84994 | HMDB02264 |
| LC/MS Pos Early | 2141 | 269.1244 | 9903482 | 8079136 | HMDB12881 |
| LC/MS Pos Late | 2858 | 746.5694 | | | HMDB08993 |
| LC/MS Pos Early | 1961 | 104.107 | 305 | 149278 | HMDB00097 |
| LC/MS Pos Early | 2910 | 315.1485 | 3001055 | 2272523 | HMDB01930 |
| LC/MS Neg | 3698.1 | 240.9139 | | | |
| LC/MS Neg | 2040 | 238.9694 | | | |
| LC/MS Neg | 4538 | 185.1182 | | | |
| LC/MS Neg | 1778.1 | 257.0779 | 445408 | 393058 | HMDB00884 |
| LC/MS Neg | 5599 | 597.3045 | | | |
| LC/MS Neg | 1840 | 131.0714 | 83697 | 75520 | HMDB00746 |
| LC/MS Neg | 3320 | 199.0071 | 6426766 | 4932200 | HMDB04072 |
| LC/MS Neg | 5080 | 371.1898 | | | |
| LC/MS Pos Late | 2893 | 538.5194 | 5283564 | 4446677 | HMDB04949 |
| LC/MS Neg | 4430 | 321.148 | | | |
| LC/MS Pos Early | 2700 | 247.1289 | 7015683 | | HMDB11172 |
| LC/MS Neg | 3871 | 383.1534 | | | HMDB02759 |
| LC/MS Polar | 2140 | 187.0724 | 182230 | 158492 | HMDB06029 |
| LC/MS Neg | 2990 | 229.1445 | 12736 | 12213 | HMDB00623 |
| LC/MS Neg | 880 | 134.0281 | 24417 | 22826 | HMDB02108 |
| LC/MS Neg | 4449 | 505.2082 | | | |
| LC/MS Pos Early | 1189 | 226.1283 | | | |

| | | | | | |
|---|---|---|---|---|---|
| LC/MS Neg | 6353.3 | 227.2016 | 79050 | 71382 | HMDB11561 |
| LC/MS Neg | 3975 | 462.1768 | | | |
| LC/MS Neg | 1800 | 131.0714 | 164623 | 144317 | HMDB00317 |
| LC/MS Pos Early | 2537 | 222.0791 | | | |
| LC/MS Neg | 3823 | 333.2077 | | | |
| LC/MS Polar | 2022.2 | 225.0616 | 5984 | 5764 | HMDB00660 |
| LC/MS Pos Late | 1393 | 300.2897 | 5353955 | 4510275 | HMDB00252 |
| LC/MS Pos Early | 2230 | 131.1179 | 122356 | 109095 | HMDB02064 |
| LC/MS Pos Late | 1485 | 428.3734 | 6426855 | 21233653 | HMDB00848 |
| LC/MS Pos Early | 597 | 154.0169 | 109 | 107 | HMDB00996 |
| LC/MS Neg | 4678 | 541.2647 | | | |
| LC/MS Neg | 1969 | 209.0454 | | | |
| LC/MS Neg | 4336 | 263.6283 | | | |
| LC/MS Neg | 3354 | 229.0176 | | | |
| LC/MS Pos Early | 1112 | 116.0707 | | | |
| LC/MS Pos Early | 2550 | 177.1022 | 5202 | 5013 | HMDB00259 |
| LC/MS Pos Early | 2100 | 212.1028 | | | |
| LC/MS Neg | 3862 | 257.1761 | | | |
| LC/MS Neg | 2150 | 135.0452 | 999 | 10181341 | HMDB00209 |
| LC/MS Neg | 3277.6 | 232.9949 | | | |
| LC/MS Pos Early | 1728 | 284.099 | 6802 | 6544 | HMDB00133 |
| LC/MS Neg | 1498.7 | 218.1034 | 6613 | 6361 | HMDB00210 |
| LC/MS Neg | 960 | 130.051 | 98681 | 89122 | HMDB00783 |
| LC/MS Neg | 2930 | 243.0783 | | | |
| LC/MS Neg | 1475 | 194.0459 | 151012 | 133104 | HMDB13678 |
| LC/MS Neg | 2021 | 227.9973 | | | |
| LC/MS Neg | 4643 | 253.0832 | | | |
| LC/MS Neg | 1157 | 128.0715 | | | |
| LC/MS Neg | 1808 | 233.0125 | | | |
| LC/MS Neg | 1745 | 241.9768 | | | |
| LC/MS Pos Late | 1627 | 349.2732 | | | |
| LC/MS Pos Early | 1531 | 191.1023 | | | |
| LC/MS Pos Early | 1272 | 166.0533 | 158980 | 139840 | HMDB02005 |
| LC/MS Pos Late | 1916 | 568.428 | | | |
| LC/MS Neg | 3597.7 | 229.0179 | | | |
| LC/MS Neg | 5275 | 295.2283 | 43013 | | |
| LC/MS Polar | 3143 | 174.0408 | 65065 | 58576 | HMDB00812 |
| LC/MS Neg | 5150 | 514.2844 | 6675 | | HMDB00036 |
| LC/MS Pos Early | 1750 | 158.1176 | 441447 | 390180 | HMDB33433 |
| LC/MS Pos Early | 1488 | 122.027 | 5862 | 5653 | HMDB00574 |
| LC/MS Pos Early | 2790 | 163.1077 | 1029 | 1002 | HMDB00450 |
| LC/MS Pos Late | 2400 | 720.5874 | | | |
| LC/MS Neg | 3215 | 266.0179 | | | |
| LC/MS Neg | 1550 | 164.0355 | | | |
| LC/MS Neg | 6250 | 279.2329 | 5365676 | 4517636 | HMDB11538 |
| LC/MS Neg | 2156 | 172.9914 | 74426 | 67018 | HMDB60015 |
| LC/MS Polar | 2070.4 | 105.0193 | 752 | 732 | HMDB00139 |
| LC/MS Neg | 5499 | 457.2361 | | | |
| LC/MS Pos Late | 2220 | 690.5432 | | 24767479 | |
| LC/MS Neg | 4396.5 | 283.1919 | | | |

| | | | | | |
|---|---|---|---|---|---|
| LC/MS Neg | 3971 | 259.0282 | | | |
| LC/MS Neg | 4503 | 266.8889 | | | |
| LC/MS Neg | 1485 | 167.0468 | | | |
| LC/MS Neg | 3515 | 329.0004 | 3440 | 3322 | HMDB01933 |
| LC/MS Neg | 3730 | 337.1427 | | | |
| LC/MS Pos Early | 3378 | 325.1911 | 2728270 | 2010267 | |
| LC/MS Pos Late | 2181 | 748.5276 | 5283497 | 4446616 | |
| LC/MS Pos Early | 1792 | 174.0872 | | | |
| LC/MS Pos Early | 1100 | 245.0768 | 6029 | 5807 | HMDB00296 |
| LC/MS Pos Early | 1700 | 90.055 | 5950 | 5735 | HMDB00161 |
| LC/MS Neg | 990 | 260.0234 | 514186 | 448617 | |
| LC/MS Pos Early | 2064 | 141.0659 | 75810 | 68319 | HMDB02820 |
| LC/MS Pos Early | 1225 | 133.0608 | 6267 | 6031 | HMDB00168 |
| LC/MS Neg | 2375 | 215.002 | 4684006 | 3872009 | |
| LC/MS Neg | 6215 | 570.3413 | 15061532 | 21403165 | HMDB61702 |
| LC/MS Pos Early | 2215 | 104.0706 | 64956 | 58481 | HMDB03911 |
| LC/MS Neg | 1690.1 | 179.035 | 979 | 954 | HMDB00707 |
| LC/MS Pos Late | 2454 | 718.5745 | 11146967 | 9322076 | HMDB11206 |
| LC/MS Pos Early | 1064 | 118.0863 | 247 | 24219951173 | HMDB00043 |
| LC/MS Polar | 870 | 885.5495 | | 21403055 | HMDB09815 |
| LC/MS Pos Late | 2401 | 776.5589 | | 24769278 | |
| LC/MS Pos Late | 2383 | 757.6218 | | | |
| LC/MS Polar | 1929.3 | 243.0623 | 15047 | 21403010 | HMDB00767 |
| LC/MS Pos Late | 2443 | 744.5902 | | | |
| LC/MS Pos Early | 1520 | 176.103 | 9750 | 810 | HMDB00904 |
| LC/MS Pos Early | 1900 | 129.0659 | 134508 | 118562 | |
| LC/MS Pos Early | 1642 | 242.0147 | | | |
| LC/MS Neg | 2580.5 | 153.0197 | | | |
| LC/MS Pos Early | 2000 | 141.0658 | | | |
| LC/MS Pos Late | 1460 | 544.3398 | | 21403155 | HMDB10395 |
| LC/MS Pos Late | 2290 | 705.5905 | 9939965 | 8115586 | |
| LC/MS Neg | 3943 | 200.1295 | | | |
| LC/MS Neg | 5525 | 327.233 | 445580 | 393183 | HMDB02183 |
| LC/MS Pos Late | 2300 | 810.6007 | 16219824 | 17347139 | HMDB08048 |
| LC/MS Pos Early | 1375 | 76.0393 | 750 | 730 | HMDB00123 |
| LC/MS Pos Late | 1860 | 673.5279 | | | |
| LC/MS Pos Late | 1474 | 482.3241 | | | |
| LC/MS Pos Late | 2160 | 758.5694 | 5287971 | 4450224 | HMDB07973 |
| LC/MS Neg | 5315 | 223.1705 | | | |
| LC/MS Pos Early | 3164 | 254.1149 | 5546 | 5345 | HMDB01940 |
| LC/MS Pos Late | 1998 | 675.5436 | 11433862 | 9608732 | HMDB12097 |

| Beta |
| --- |
| 3.042191689 |
| 2.765347928 |
| 2.750397493 |
| 2.671601458 |
| 2.527996234 |
| 2.295964511 |
| 2.175362291 |
| 1.98185332 |
| 1.701393093 |
| 1.611011761 |
| 1.543375225 |
| 1.453908261 |
| 1.42998924 |
| 1.375928552 |
| 1.375382929 |
| 1.277513016 |
| 1.134439109 |
| 1.055089182 |
| 1.047342526 |
| 0.980014078 |
| 0.918175572 |
| 0.910854355 |
| 0.897956826 |
| 0.897083604 |
| 0.87944214 |
| 0.843897259 |
| 0.83812825 |
| 0.796319988 |
| 0.773152806 |
| 0.759766967 |
| 0.734834682 |
| 0.707636712 |
| 0.699256636 |
| 0.691365505 |
| 0.676034864 |
| 0.664682329 |
| 0.657132414 |
| 0.598247936 |
| 0.576639993 |
| 0.570501005 |
| 0.560401864 |
| 0.518377223 |
| 0.514154942 |
| 0.503149611 |
| 0.501864662 |
| 0.490223091 |
| 0.47996596 |
| 0.475794977 |

0.474932129
0.451550492
0.434517026
0.425885164
0.401902625
0.397203417
0.374949412
0.366574277
0.34683909
0.315557527
0.312907334
0.31010242
0.287816194
0.273505093
0.234453566
0.227437022
0.220791091
-0.141274601
-0.181728868
-0.199519076
-0.222979495
-0.236636799
-0.242395598
-0.249415739
-0.307792446
-0.335142904
-0.343294472
-0.348920717
-0.383014809
-0.388523676
-0.404499525
-0.40669467
-0.427221073
-0.435185292
-0.440551158
-0.44670495
-0.446852703
-0.454640013
-0.467175939
-0.470340932
-0.474422221
-0.507290299
-0.510285542
-0.515693771
-0.529204553
-0.535241488
-0.537228668
-0.539034225
-0.553642135
-0.55634498

| |
|---|
| -0.558706559 |
| -0.566295401 |
| -0.578491493 |
| -0.603774044 |
| -0.613365016 |
| -0.625361328 |
| -0.628028712 |
| -0.633081233 |
| -0.634500795 |
| -0.635341432 |
| -0.65191283 |
| -0.653698092 |
| -0.664624022 |
| -0.668383385 |
| -0.683171726 |
| -0.73174674 |
| -0.734782733 |
| -0.756473805 |
| -0.756659927 |
| -0.759202352 |
| -0.879504352 |
| -0.964659423 |
| -1.005850478 |
| -1.046092941 |
| -1.091709742 |
| -1.125186336 |
| -1.130031679 |
| -1.155137021 |
| -1.163263148 |
| -1.191797205 |
| -1.244759629 |
| -1.322954594 |
| -1.345452548 |
| -1.383379048 |
| -1.38689238 |
| -1.459574406 |
| -1.472994874 |
| -1.590887566 |
| -1.701358023 |
| -1.878889307 |
| -2.751450285 |

**Supplementary Table 3. Overview of metabolites included fo**

| BIOCHEMICAL |
| --- |
| trans-4-hydroxyproline |
| 1-palmityl-2-arachidonoyl-GPC (O-16:0/20:4)* |
| oleoylcarnitine (C18:1) |
| choline |
| X - 11787 |
| 1-(1-enyl-stearoyl)-2-arachidonoyl-GPE (P-18:0/20:4)* |
| 1-stearoyl-2-linoleoyl-GPE (18:0/18:2)* |
| isovalerylcarnitine (C5) |
| pregnen-diol disulfate C21H34O8S2* |
| mannose |
| N6,N6,N6-trimethyllysine |
| X - 24293 |
| 1-(1-enyl-oleoyl)-GPE (P-18:1)* |
| o-cresol sulfate |
| 1-methylhistidine |
| X - 12798 |
| 5alpha-androstan-3alpha,17beta-diol monosulfate (2) |
| O-methylcatechol sulfate |
| beta-alanine |
| 1,5-anhydroglucitol (1,5-AG) |
| 3-methylglutarylcarnitine (2) |
| 3-(4-hydroxyphenyl)lactate |
| X - 02269 |
| androstenediol (3alpha, 17alpha) monosulfate (3) |
| 1-oleoylglycerol (18:1) |
| X - 11852 |
| sarcosine |
| X - 12830 |
| 1-(1-enyl-palmitoyl)-2-oleoyl-GPE (P-16:0/18:1)* |
| X - 02249 |
| X - 23997 |
| X - 21792 |
| X - 17337 |
| X - 11381 |
| X - 16649 |
| theanine |
| X - 13431 |
| isoeugenol sulfate |
| galactote |
| N-acetylhistidine |
| fumarate |
| gamma-glutamyl-epsilon-lysine |
| X - 17189 |
| N-acetylputrescine |
| methionine sulfoxide |

estrone 3-sulfate
gamma-glutamylglycine
X - 21752
X - 11849
X - 12544
N-acetylalliin
taurocholate
3-ureidopropiote
S-allylcysteine
asparagine
2-hydroxydecanoate
phenol sulfate
myo-inositol
2-arachidonoyl-GPC (20:4)*
X - 12739
methionine sulfone
cortisol
androstenediol (3beta,17beta) monosulfate (2)
X - 21442
tryptophan betaine
X - 18901
threonine
7-methylguanine
erythrote*
palmitoyl dihydrosphingomyelin (d18:0/16:0)*
N-acetylalanine
1-methylnicotimide
nicotimide
1-methylimidazoleacetate
1-stearoyl-2-arachidonoyl-GPC (18:0/20:4)
1-palmityl-2-palmitoyl-GPC (O-16:0/16:0)*
X - 11315
S-methylcysteine
3-methylhistidine
trimethylamine N-oxide
N-trimethyl 5-aminovalerate
sphingomyelin (d18:2/14:0, d18:1/14:1)*

**r the processed meat metabolite score**

| Sub_pathway | Super_pathway | PLATFORM |
|---|---|---|
| Urea cycle; Arginine and Proline Metabolism | Amino Acid | LC/MS Pos Early |
| Plasmalogen | Lipid | LC/MS Pos Late |
| Fatty Acid Metabolism(Acyl Carnitine) | Lipid | LC/MS Pos Late |
| Phospholipid Metabolism | Lipid | LC/MS Pos Early |
| Unknown | Unknown | LC/MS Pos Early |
| Plasmalogen | Lipid | LC/MS Pos Late |
| Phosphatidylethanolamine (PE) | Lipid | LC/MS Pos Late |
| Leucine, Isoleucine and Valine Metabolism | Amino Acid | LC/MS Pos Early |
| Progestin Steroids | Lipid | LC/MS Neg |
| Fructose, Mannose and Galactose Metabolism | Carbohydrate | LC/MS Polar |
| Lysine Metabolism | Amino Acid | LC/MS Pos Early |
| Unknown | Unknown | LC/MS Pos Early |
| Lysoplasmalogen | Lipid | LC/MS Pos Late |
| Benzoate Metabolism | Xenobiotics | LC/MS Neg |
| Histidine Metabolism | Amino Acid | LC/MS Pos Early |
| Unknown | Unknown | LC/MS Pos Early |
| Androgenic Steroids | Lipid | LC/MS Neg |
| Benzoate Metabolism | Xenobiotics | LC/MS Neg |
| Pyrimidine Metabolism, Uracil containing | Nucleotide | LC/MS Pos Early |
| Glycolysis, Gluconeogenesis, and Pyruvate Metabolism | Carbohydrate | LC/MS Neg |
| Leucine, Isoleucine and Valine Metabolism | Amino Acid | LC/MS Pos Early |
| Tyrosine Metabolism | Amino Acid | LC/MS Neg |
| Unknown | Unknown | LC/MS Neg |
| Androgenic Steroids | Lipid | LC/MS Neg |
| Monoacylglycerol | Lipid | LC/MS Neg |
| Unknown | Unknown | LC/MS Neg |
| Glycine, Serine and Threonine Metabolism | Amino Acid | LC/MS Pos Early |
| Unknown | Unknown | LC/MS Neg |
| Plasmalogen | Lipid | LC/MS Pos Late |
| Unknown | Unknown | LC/MS Neg |
| Unknown | Unknown | LC/MS Pos Early |
| Unknown | Unknown | LC/MS Neg |
| Unknown | Unknown | LC/MS Pos Late |
| Unknown | Unknown | LC/MS Neg |
| Unknown | Unknown | LC/MS Neg |
| Food Component/Plant | Xenobiotics | LC/MS Neg |
| Unknown | Unknown | LC/MS Pos Late |
| Food Component/Plant | Xenobiotics | LC/MS Neg |
| Fructose, Mannose and Galactose Metabolism | Carbohydrate | LC/MS Polar |
| Histidine Metabolism | Amino Acid | LC/MS Pos Early |
| TCA Cycle | Energy | LC/MS Polar |
| Gamma-glutamyl Amino Acid | Peptide | LC/MS Pos Early |
| Unknown | Unknown | LC/MS Neg |
| Polyamine Metabolism | Amino Acid | LC/MS Pos Early |
| Methionine, Cysteine, SAM and Taurine Metabolism | Amino Acid | LC/MS Pos Early |

| | | |
|---|---|---|
| Estrogenic Steroids | Lipid | LC/MS Neg |
| Gamma-glutamyl Amino Acid | Peptide | LC/MS Pos Early |
| Unknown | Unknown | LC/MS Neg |
| Unknown | Unknown | LC/MS Neg |
| Unknown | Unknown | LC/MS Neg |
| Food Component/Plant | Xenobiotics | LC/MS Neg |
| Primary Bile Acid Metabolism | Lipid | LC/MS Neg |
| Pyrimidine Metabolism, Uracil containing | Nucleotide | LC/MS Pos Early |
| Food Component/Plant | Xenobiotics | LC/MS Pos Early |
| Alanine and Aspartate Metabolism | Amino Acid | LC/MS Pos Early |
| Fatty Acid, Monohydroxy | Lipid | LC/MS Neg |
| Tyrosine Metabolism | Amino Acid | LC/MS Neg |
| Inositol Metabolism | Lipid | LC/MS Polar |
| Lysophospholipid | Lipid | LC/MS Neg |
| Unknown | Unknown | LC/MS Neg |
| Methionine, Cysteine, SAM and Taurine Metabolism | Amino Acid | LC/MS Pos Early |
| Corticosteroids | Lipid | LC/MS Neg |
| Androgenic Steroids | Lipid | LC/MS Neg |
| Unknown | Unknown | LC/MS Neg |
| Tryptophan Metabolism | Amino Acid | LC/MS Pos Early |
| Unknown | Unknown | LC/MS Neg |
| Glycine, Serine and Threonine Metabolism | Amino Acid | LC/MS Pos Early |
| Purine Metabolism, Guanine containing | Nucleotide | LC/MS Pos Early |
| Aminosugar Metabolism | Carbohydrate | LC/MS Polar |
| Sphingolipid Metabolism | Lipid | LC/MS Pos Late |
| Alanine and Aspartate Metabolism | Amino Acid | LC/MS Neg |
| Nicotite and Nicotimide Metabolism | Cofactors and Vitamins | LC/MS Pos Early |
| Nicotite and Nicotimide Metabolism | Cofactors and Vitamins | LC/MS Pos Early |
| Histidine Metabolism | Amino Acid | LC/MS Pos Early |
| Phosphatidylcholine (PC) | Lipid | LC/MS Pos Late |
| Plasmalogen | Lipid | LC/MS Pos Late |
| Unknown | Unknown | LC/MS Neg |
| Methionine, Cysteine, SAM and Taurine Metabolism | Amino Acid | LC/MS Neg |
| Histidine Metabolism | Amino Acid | LC/MS Neg |
| Phospholipid Metabolism | Lipid | LC/MS Pos Early |
| Lysine Metabolism | Amino Acid | LC/MS Pos Early |
| Sphingolipid Metabolism | Lipid | LC/MS Pos Late |

| Retention_Index | MASS | PubChem | ChemSpider | HMBD_ID | Beta |
|---|---|---|---|---|---|
| 1064 | 132.0655 | 5810 | 5605 | HMDB00725 | 2.712106182 |
| 2183 | 768.5902 | 6443139 | 4947173 | | 2.386297619 |
| 1423 | 426.3578 | 6441392 | | HMDB05065 | 1.060299314 |
| 1961 | 104.107 | 305 | 149278 | HMDB00097 | 0.909178486 |
| 2319 | 148.0968 | | | | 0.902136385 |
| 2511 | 752.5589 | 9547058 | 7826008 | HMDB05779 | 0.858712564 |
| 2522 | 744.5538 | 9546749 | 7825699 | HMDB08994 | 0.824785693 |
| 3085 | 246.17 | 6426851 | | HMDB00688 | 0.780659333 |
| 3868 | 238.0775 | | | | 0.714854755 |
| 2200 | 225.0616 | 18950 | 141983 | HMDB00169 | 0.709813088 |
| 2825 | 189.1598 | 440120 | 140379 | HMDB01325 | 0.667682545 |
| 1189 | 226.1283 | | | | 0.63113102 |
| 1566 | 464.3136 | | | | 0.618170451 |
| 2796 | 187.0071 | 11615528 | 9790277 | | 0.589318351 |
| 2755 | 170.0924 | 92105 | 83153 | HMDB00001 | 0.556717709 |
| 2011 | 240.1017 | | | | 0.522097933 |
| 5080 | 371.1898 | | | | 0.498809193 |
| 2344 | 203.002 | 22473 | 21078 | HMDB60013 | 0.491182115 |
| 1939 | 90.055 | 239 | 234 | HMDB00056 | 0.472851485 |
| 802 | 163.0612 | 64960 | | HMDB02712 | 0.466526578 |
| 2675 | 290.1598 | 128145 | 113619 | HMDB00552 | 0.456034181 |
| 1379 | 181.0506 | 9378 | 9010 | HMDB00755 | 0.442029898 |
| 1525.4 | 255.0876 | | | | 0.435946134 |
| 5180 | 369.1741 | | | | 0.407322815 |
| 6500 | 281.2486 | 5283468 | 4446588 | HMDB11567 | 0.386304852 |
| 3277.6 | 232.9949 | | | | 0.385331942 |
| 1280 | 90.055 | 1088 | 1057 | HMDB00271 | 0.381784472 |
| 3197 | 372.1128 | | | | 0.377105915 |
| 2600 | 702.5432 | | 24769228 | HMDB11342 | 0.372129887 |
| 4012 | 267.1239 | | | | 0.365875875 |
| 1634 | 223.0745 | | | | 0.360853658 |
| 1290 | 197.0827 | | | | 0.360289185 |
| 1136 | 328.2476 | | | | 0.338684853 |
| 1118 | 184.0982 | | | | 0.332568551 |
| 3018 | 353.0346 | | | | 0.298753205 |
| 1180 | 173.0932 | 439378 | 388498 | HMDB34365 | 0.221059256 |
| 1015 | 302.2322 | | | | 0.201234964 |
| 3893 | 243.0333 | | | | 0.19099696 |
| 3085 | 195.051 | 128869 | 114198 | HMDB00565 | -0.140135327 |
| 2065 | 198.0873 | 75619 | 68142 | HMDB32055 | -0.184676344 |
| 3084 | 115.0037 | 444972 | 10197150 | HMDB00134 | -0.189258967 |
| 2717 | 276.1554 | 7015684;7015685 | 5378717 | HMDB03869 | -0.236534217 |
| 2399 | 411.4896 | | | | -0.257993375 |
| 2230 | 131.1179 | 122356 | 109095 | HMDB02064 | -0.2658603 |
| 1272 | 166.0533 | 158980 | 139840 | HMDB02005 | -0.301655882 |

| | | | | | |
|---|---|---|---|---|---|
| 4417.3 | 349.1115 | 3001028 | 16740220 | HMDB01425 | -0.309126278 |
| 1535 | 205.0819 | 165527 | 145071 | HMDB11667 | -0.313947657 |
| 5090 | 621.3307 | | | | -0.317160886 |
| 3215 | 266.0179 | | | | -0.318950077 |
| 3166.9 | 209.0822 | | | | -0.321926059 |
| 1467 | 218.0493 | | | | -0.327829695 |
| 5150 | 514.2844 | 6675 | | HMDB00036 | -0.333568025 |
| 875 | 133.0608 | 111 | 109 | HMDB00026 | -0.338106162 |
| 2690 | 162.0583 | 98280 | 88744 | HMDB34323 | -0.37301125 |
| 1225 | 133.0608 | 6267 | 6031 | HMDB00168 | -0.381107325 |
| 4840 | 187.134 | 21488 | 20195 | | -0.39469837 |
| 2156 | 172.9914 | 74426 | 67018 | HMDB60015 | -0.419226077 |
| 3506.3 | 225.0616 | 892 | 10239179 | HMDB00211 | -0.428902312 |
| 5965 | 618.3413 | | 21403158 | HMDB61699 | -0.430520506 |
| 2406 | 241.1195 | | | | -0.484350261 |
| 1250 | 182.0482 | 69961 | 63154 | | -0.514829225 |
| 4713.2 | 361.202 | 5754 | | HMDB00063 | -0.516035894 |
| 4500 | 369.1741 | | | | -0.53631549 |
| 3823 | 333.2077 | | | | -0.543314794 |
| 2673 | 247.1441 | 442106 | | HMDB61115 | -0.560502359 |
| 3800 | 211.0247 | | | | -0.563198775 |
| 1514 | 120.0655 | 6288 | 6051 | HMDB00167 | -0.580069014 |
| 2175 | 166.0723 | 11361 | 10883 | HMDB00897 | -0.582891771 |
| 2186 | 135.0299 | 2781043 | 2061231 | HMDB00613 | -0.583402709 |
| 2290 | 705.5905 | 9939965 | 8115586 | | -0.593952949 |
| 861.2 | 130.051 | 88064 | 79449 | HMDB00766 | -0.623925693 |
| 1940 | 137.0709 | 10129985 | 8305504 | HMDB00699 | -0.665002998 |
| 1942 | 123.0553 | 936 | 911 | HMDB01406 | -0.703684813 |
| 2064 | 141.0659 | 75810 | 68319 | HMDB02820 | -0.721353202 |
| 2300 | 810.6007 | 16219824 | 17347139 | HMDB08048 | -0.725572799 |
| 2400 | 720.5874 | | | | -0.728891836 |
| 1157 | 128.0715 | | | | -0.874234672 |
| 880 | 134.0281 | 24417 | 22826 | HMDB02108 | -0.896197351 |
| 906.3 | 168.0779 | 64969 | 58494 | HMDB00479 | -0.901212513 |
| 2100 | 76.0757 | 1145 | 1113 | HMDB00925 | -0.914916251 |
| 2186 | 160.1332 | | | | -1.496211186 |
| 1860 | 673.5279 | | | | -1.656381993 |

**Supplementary Table 4. Overview of metabolites included for the p**

| BIOCHEMICAL |
|---|
| 1-(1-enyl-palmitoyl)-2-arachidonoyl-GPE (P-16:0/20:4)* |
| 3-methylhistidine |
| X - 23652 |
| X - 14838 |
| ibuprofen |
| X - 11787 |
| serine |
| 1-(1-enyl-palmitoyl)-2-docosahexaenoyl-GPC (P-16:0/22:6)* |
| X - 13835 |
| bilirubin (E,E)* |
| taurolithocholate 3-sulfate |
| kynurete |
| S-allylcysteine |
| valerate |
| N-acetyltryptophan |
| trans-4-hydroxyproline |
| N-acetyl-beta-alanine |
| 5alpha-pregn-3beta,20beta-diol monosulfate (1) |
| X - 11849 |
| phosphate |
| lanthionine |
| uracil |
| X - 12127 |
| sphingosine |
| X - 21752 |
| N-acetyltaurine |
| X - 12739 |
| X - 21365 |
| pyrraline |
| glycocholete sulfate* |
| 13-HODE + 9-HODE |
| malate |
| X - 24309 |
| N-acetylglutamate |
| phenyllactate (PLA) |
| 1-margaroyl-2-linoleoyl-GPC (17:0/18:2)* |
| phenylacetate |
| N-acetylputrescine |
| 1-eicosenoyl-GPC (20:1)* |
| 3-methoxytyrosine |
| X - 12100 |
| N-acetylneuramite |
| 4-acetamidobutanoate |
| X - 13431 |
| pyroglutamine* |

methionine sulfone
sphingomyelin (d18:2/14:0, d18:1/14:1)*
1-(1-enyl-stearoyl)-2-arachidonoyl-GPC (P-18:0/20:4)
trimethylamine N-oxide

**oultry metabolite score**

| Sub_pathway | Super_pathway | PLATFORM |
|---|---|---|
| Plasmalogen | Lipid | LC/MS Pos Late |
| Histidine Metabolism | Amino Acid | LC/MS Neg |
| Unknown | Unknown | LC/MS Pos Early |
| Unknown | Unknown | LC/MS Pos Early |
| Drug | Xenobiotics | LC/MS Neg |
| Unknown | Unknown | LC/MS Pos Early |
| Glycine, Serine and Threonine Metabolism | Amino Acid | LC/MS Pos Early |
| Plasmalogen | Lipid | LC/MS Pos Late |
| Unknown | Unknown | LC/MS Neg |
| Hemoglobin and Porphyrin Metabolism | Cofactors and Vitamins | LC/MS Pos Late |
| Secondary Bile Acid Metabolism | Lipid | LC/MS Neg |
| Tryptophan Metabolism | Amino Acid | LC/MS Pos Early |
| Food Component/Plant | Xenobiotics | LC/MS Pos Early |
| Short Chain Fatty Acid | Lipid | LC/MS Neg |
| Tryptophan Metabolism | Amino Acid | LC/MS Pos Early |
| Urea cycle; Arginine and Proline Metabolism | Amino Acid | LC/MS Pos Early |
| Pyrimidine Metabolism, Uracil containing | Nucleotide | LC/MS Neg |
| Progestin Steroids | Lipid | LC/MS Neg |
| Unknown | Unknown | LC/MS Neg |
| Oxidative Phosphorylation | Energy | LC/MS Pos Early |
| Chemical | Xenobiotics | LC/MS Pos Early |
| Pyrimidine Metabolism, Uracil containing | Nucleotide | LC/MS Polar |
| Unknown | Unknown | LC/MS Pos Early |
| Sphingolipid Metabolism | Lipid | LC/MS Pos Late |
| Unknown | Unknown | LC/MS Neg |
| Methionine, Cysteine, SAM and Taurine Metabolism | Amino Acid | LC/MS Neg |
| Unknown | Unknown | LC/MS Neg |
| Lysine Metabolism | Amino Acid | LC/MS Pos Early |
| Food Component/Plant | Xenobiotics | LC/MS Neg |
| Secondary Bile Acid Metabolism | Lipid | LC/MS Neg |
| Fatty Acid, Monohydroxy | Lipid | LC/MS Neg |
| TCA Cycle | Energy | LC/MS Neg |
| Unknown | Unknown | LC/MS Neg |
| Glutamate Metabolism | Amino Acid | LC/MS Polar |
| Phenylalanine Metabolism | Amino Acid | LC/MS Neg |
| Phosphatidylcholine (PC) | Lipid | LC/MS Pos Late |
| Phenylalanine Metabolism | Amino Acid | LC/MS Neg |
| Polyamine Metabolism | Amino Acid | LC/MS Pos Early |
| Lysophospholipid | Lipid | LC/MS Pos Late |
| Tyrosine Metabolism | Amino Acid | LC/MS Pos Early |
| Unknown | Unknown | LC/MS Pos Early |
| Aminosugar Metabolism | Carbohydrate | LC/MS Pos Early |
| Polyamine Metabolism | Amino Acid | LC/MS Neg |
| Unknown | Unknown | LC/MS Pos Late |
| Glutamate Metabolism | Amino Acid | LC/MS Pos Early |

| | | |
|---|---|---|
| Methionine, Cysteine, SAM and Taurine Metabolism | Amino Acid | LC/MS Pos Early |
| Sphingolipid Metabolism | Lipid | LC/MS Pos Late |
| Plasmalogen | Lipid | LC/MS Pos Late |
| Phospholipid Metabolism | Lipid | LC/MS Pos Early |

| Retention_Index | MASS | PubChem | ChemSpider | HMBD_ID | Beta |
|---|---|---|---|---|---|
| 2270 | 724.5276 | | 24769238 | HMDB11352 | 2.99219766 |
| 906.3 | 168.0779 | 64969 | 58494 | HMDB00479 | 2.484591916 |
| 1991 | 171.0761 | | | | 2.208646224 |
| 2000 | 141.0658 | | | | 2.001496418 |
| 4925 | 205.1234 | 3672 | 3544 | HMDB01925 | 1.154806129 |
| 2319 | 148.0968 | | | | 0.954141873 |
| 1239 | 106.0499 | 5951 | 5736 | HMDB00187 | 0.83860118 |
| 2041 | 790.5745 | | | | 0.789095059 |
| 1017 | 169.0618 | | | | 0.717051018 |
| 1045 | 585.2708 | 5315454 | 4474753 | | 0.687198377 |
| 5026.7 | 280.6221 | 440071 | 389078 | HMDB02580 | 0.633456478 |
| 2385 | 190.0499 | 3845 | 3712 | HMDB00715 | 0.589003219 |
| 2690 | 162.0583 | 98280 | 88744 | HMDB34323 | 0.55961595 |
| 1718 | 101.0608 | 7991 | 7701 | HMDB00892 | 0.46242741 |
| 2640 | 247.1077 | 700653 | 610602 | HMDB13713 | 0.445244427 |
| 1064 | 132.0655 | 5810 | 5605 | HMDB00725 | 0.442879819 |
| 773 | 130.051 | 76406 | 68881 | | 0.426923627 |
| 5180 | 399.2211 | | | | 0.411862809 |
| 3215 | 266.0179 | | | | 0.353292733 |
| 576 | 98.9842 | 1061 | 1032 | HMDB01429 | 0.339295943 |
| 1730 | 209.0591 | 6994972;98504 | 88959 | | 0.28884462 |
| 1089.7 | 111.02 | 1174 | 1141 | HMDB00300 | 0.204846929 |
| 2160 | 226.0817 | | | | -0.301430749 |
| 1393 | 300.2897 | 5353955 | 4510275 | HMDB00252 | -0.306766043 |
| 5090 | 621.3307 | | | | -0.318337893 |
| 800 | 166.018 | 159864 | 140553 | | -0.338861086 |
| 2406 | 241.1195 | | | | -0.356586457 |
| 2195 | 160.1333 | | | | -0.373315252 |
| 2304 | 253.1194 | | 19980286 | HMDB33143 | -0.438483391 |
| 4750 | 254.6229 | | | | -0.459264524 |
| 5275 | 295.2283 | 43013 | | | -0.48465637 |
| 615.6 | 133.0143 | 525 | 510 | HMDB00156 | -0.499875568 |
| 4430 | 321.148 | | | | -0.54953697 |
| 3106 | 188.0564 | 70914 | 1266066 | HMDB01138 | -0.550950624 |
| 2208 | 165.0557 | 3848 | 3715 | HMDB00779 | -0.580365888 |
| 2242 | 772.5851 | | | | -0.586324184 |
| 2150 | 135.0452 | 999 | 10181341 | HMDB00209 | -0.615055956 |
| 2230 | 131.1179 | 122356 | 109095 | HMDB02064 | -0.620669247 |
| 1630 | 550.3867 | | | | -0.626938841 |
| 2555 | 212.0917 | 1670 | 8948 | HMDB01434 | -0.628345253 |
| 2650 | 221.0919 | | | | -0.657663085 |
| 660 | 310.1133 | 439197 | 10292217 | HMDB00230 | -0.671371775 |
| 893.7 | 144.0666 | 18189 | 17180 | HMDB03681 | -0.769661334 |
| 1015 | 302.2322 | | | | -0.877268869 |
| 1900 | 129.0659 | 134508 | 118562 | | -0.946378781 |

| | | | | | |
|---|---|---|---|---|---|
| 1250 | 182.0482 | 69961 | 63154 | | -0.964208425 |
| 1860 | 673.5279 | | | | -1.094598248 |
| 2350 | 794.6058 | | 24767528 | | -1.342151679 |
| 2100 | 76.0757 | 1145 | 1113 | HMDB00925 | -1.628258621 |

**Supplementary Table 5. The parameters of feature (metabolite) selection using bootstrapping enhanc**

| tstrap num | r2 | RMSE | lambda | alpha |
|---|---|---|---|---|
| 1 | 0.24 | 702.90 | 0.21 | 0.85 |
| 2 | 0.25 | 706.76 | 0.74 | 0.25 |
| 3 | 0.25 | 687.02 | 0.23 | 0.7 |
| 4 | 0.26 | 691.75 | 0.33 | 0.45 |
| 5 | 0.25 | 682.02 | 0.19 | 0.85 |
| 6 | 0.25 | 702.46 | 0.18 | 0.75 |
| 7 | 0.25 | 698.87 | 0.19 | 0.85 |
| 8 | 0.25 | 695.39 | 0.36 | 0.4 |
| 9 | 0.26 | 694.62 | 0.69 | 0.2 |
| 10 | 0.24 | 690.44 | 0.24 | 0.8 |
| 11 | 0.25 | 693.70 | 0.32 | 0.5 |
| 12 | 0.24 | 684.82 | 0.38 | 0.45 |
| 13 | 0.25 | 688.98 | 0.31 | 0.5 |
| 14 | 0.25 | 689.96 | 0.32 | 0.45 |
| 15 | 0.24 | 699.11 | 1.16 | 0.15 |
| 16 | 0.24 | 693.72 | 0.63 | 0.25 |
| 17 | 0.25 | 686.37 | 0.46 | 0.35 |
| 18 | 0.25 | 697.92 | 0.22 | 0.7 |
| 19 | 0.25 | 702.72 | 0.23 | 0.7 |
| 20 | 0.24 | 701.10 | 0.36 | 0.45 |
| 21 | 0.26 | 685.66 | 0.20 | 0.65 |
| 22 | 0.25 | 688.44 | 0.72 | 0.2 |
| 23 | 0.25 | 686.81 | 0.31 | 0.45 |
| 24 | 0.24 | 713.05 | 0.38 | 0.5 |
| 25 | 0.25 | 675.12 | 0.54 | 0.3 |
| 26 | 0.25 | 682.93 | 0.98 | 0.15 |
| 27 | 0.25 | 699.17 | 1.10 | 0.15 |
| 28 | 0.25 | 696.77 | 0.24 | 0.6 |
| 29 | 0.26 | 680.71 | 0.18 | 0.8 |
| 30 | 0.24 | 694.90 | 1.57 | 0.1 |
| 31 | 0.26 | 680.42 | 0.14 | 0.85 |
| 32 | 0.25 | 690.38 | 0.32 | 0.6 |
| 33 | 0.25 | 680.39 | 0.97 | 0.15 |
| 34 | 0.25 | 683.73 | 0.64 | 0.25 |
| 35 | 0.25 | 712.30 | 0.21 | 0.75 |
| 36 | 0.23 | 699.04 | 0.26 | 0.8 |
| 37 | 0.24 | 701.63 | 0.54 | 0.3 |
| 38 | 0.25 | 682.38 | 0.64 | 0.25 |
| 39 | 0.25 | 684.28 | 0.20 | 0.85 |
| 40 | 0.23 | 684.52 | 0.87 | 0.25 |
| 41 | 0.24 | 691.60 | 0.39 | 0.45 |
| 42 | 0.23 | 704.75 | 0.65 | 0.3 |
| 43 | 0.25 | 685.99 | 0.32 | 0.55 |
| 44 | 0.26 | 700.42 | 0.83 | 0.15 |
| 45 | 0.25 | 698.24 | 0.21 | 0.7 |

| | | | | |
|---|---|---|---|---|
| 46 | 0.24 | 682.10 | 0.26 | 0.6 |
| 47 | 0.25 | 692.75 | 0.98 | 0.15 |
| 48 | 0.25 | 688.66 | 0.20 | 0.75 |
| 49 | 0.24 | 695.56 | 0.79 | 0.25 |
| 50 | 0.24 | 696.55 | 0.24 | 0.75 |
| 51 | 0.23 | 686.04 | 0.27 | 0.8 |
| 52 | 0.25 | 701.35 | 1.48 | 0.1 |
| 53 | 0.25 | 699.22 | 0.66 | 0.2 |
| 54 | 0.25 | 683.09 | 0.41 | 0.4 |
| 55 | 0.23 | 689.37 | 0.58 | 0.35 |
| 56 | 0.24 | 697.67 | 0.56 | 0.35 |
| 57 | 0.25 | 691.86 | 0.45 | 0.4 |
| 58 | 0.24 | 701.39 | 0.66 | 0.3 |
| 59 | 0.25 | 687.02 | 0.27 | 0.6 |
| 60 | 0.25 | 699.87 | 0.19 | 0.7 |
| 61 | 0.25 | 699.74 | 0.33 | 0.45 |
| 62 | 0.23 | 702.22 | 0.38 | 0.5 |
| 63 | 0.25 | 703.37 | 0.90 | 0.15 |
| 64 | 0.24 | 700.47 | 0.22 | 0.9 |
| 65 | 0.24 | 694.62 | 0.36 | 0.55 |
| 66 | 0.25 | 711.04 | 0.49 | 0.3 |
| 67 | 0.26 | 679.05 | 1.18 | 0.1 |
| 68 | 0.25 | 685.34 | 0.65 | 0.25 |
| 69 | 0.25 | 673.62 | 0.24 | 0.6 |
| 70 | 0.24 | 683.88 | 1.28 | 0.15 |
| 71 | 0.25 | 679.29 | 0.27 | 0.55 |
| 72 | 0.24 | 696.13 | 0.27 | 0.8 |
| 73 | 0.25 | 705.15 | 0.33 | 0.5 |
| 74 | 0.25 | 677.07 | 0.29 | 0.55 |
| 75 | 0.25 | 683.32 | 0.18 | 0.8 |
| 76 | 0.24 | 695.14 | 0.35 | 0.5 |
| 77 | 0.26 | 694.19 | 0.35 | 0.35 |
| 78 | 0.24 | 693.71 | 0.23 | 0.7 |
| 79 | 0.25 | 698.61 | 0.22 | 0.7 |
| 80 | 0.24 | 692.05 | 0.32 | 0.55 |
| 81 | 0.24 | 678.87 | 0.25 | 0.7 |
| 82 | 0.24 | 690.60 | 0.26 | 0.65 |
| 83 | 0.25 | 689.84 | 0.53 | 0.3 |
| 84 | 0.25 | 686.18 | 0.23 | 0.7 |
| 85 | 0.25 | 708.69 | 0.35 | 0.45 |
| 86 | 0.24 | 686.04 | 0.33 | 0.6 |
| 87 | 0.25 | 683.84 | 0.18 | 0.9 |
| 88 | 0.25 | 680.07 | 0.26 | 0.55 |
| 89 | 0.25 | 681.85 | 0.22 | 0.85 |
| 90 | 0.24 | 694.26 | 0.89 | 0.2 |
| 91 | 0.26 | 684.63 | 0.27 | 0.55 |
| 92 | 0.24 | 674.10 | 0.26 | 0.7 |

| | | | | |
|---|---|---|---|---|
| 93 | 0.25 | 696.72 | 0.25 | 0.7 |
| 94 | 0.26 | 702.35 | 0.15 | 0.9 |
| 95 | 0.26 | 683.84 | 0.30 | 0.5 |
| 96 | 0.24 | 681.25 | 0.72 | 0.25 |
| 97 | 0.25 | 696.24 | 0.30 | 0.55 |
| 98 | 0.24 | 706.95 | 0.37 | 0.5 |
| 99 | 0.24 | 702.93 | 0.21 | 0.85 |
| 100 | 0.25 | 706.16 | 1.09 | 0.15 |

**ced elastic net regression**

**Supplementary Table 6. The rank of metabolites in the red meat metabolite score ordered by se**

| biochemical | super_pathway |
|---|---|
| glycine | Amino Acid |
| cholesterol | Lipid |
| creatinine | Amino Acid |
| alanine | Amino Acid |
| 4-hydroxyphenylpyruvate | Amino Acid |
| citrulline | Amino Acid |
| imidazole lactate | Amino Acid |
| sphingosine | Lipid |
| 1-palmityl-GPC (O-16:0) | Lipid |
| alpha-hydroxyisocaproate | Amino Acid |
| creatine | Amino Acid |
| trans-4-hydroxyproline | Amino Acid |
| 2-linoleoylglycerol (18:2) | Lipid |
| andro steroid monosulfate C19H28O6S (1)* | Lipid |
| homostachydrine* | Xenobiotics |
| 1-arachidonoyl-GPC (20:4n6)* | Lipid |
| N-acetylglutamine | Amino Acid |
| N-acetylphenylalanine | Amino Acid |
| 10-heptadecenoate (17:1n7) | Lipid |
| 1-myristoylglycerol (14:0) | Lipid |
| 4-vinylphenol sulfate | Xenobiotics |
| deoxycarnitine | Lipid |
| 5alpha-androstan-3alpha,17beta-diol monosulfate (2) | Lipid |
| 1-docosapentaenoyl-GPC (22:5n3)* | Lipid |
| 1-pentadecanoyl-GPC (15:0)* | Lipid |
| ergothioneine | Xenobiotics |
| 17-methylstearate | Lipid |
| S-methylcysteine | Amino Acid |
| trimethylamine N-oxide | Lipid |
| 1-stearoyl-2-oleoyl-GPE (18:0/18:1) | Lipid |
| sphingomyelin (d18:1/14:0, d16:1/16:0)* | Lipid |
| verapamil | Xenobiotics |
| N-acetylcarnosine | Peptide |
| 4-acetylphenol sulfate | Xenobiotics |
| docosahexaenoate (DHA; 22:6n3) | Lipid |
| N-palmitoyl-sphingosine (d18:1/16:0) | Lipid |
| methionine sulfone | Amino Acid |
| X - 21286 | X |
| 1-arachidonoyl-GPA (20:4) | Lipid |
| X - 11381 | X |
| X - 09789 | X |
| X - 12212 | X |
| X - 21315 | X |
| X - 12511 | X |
| X - 02249 | X |
| X - 11849 | X |
| X - 12729 | X |
| X - 15492 | X |

| | |
|---|---|
| X - 18779 | X |
| X - 18913 | X |
| X - 21659 | X |
| X - 17676 | X |
| X - 21821 | X |
| sphingomyelin (d18:2/14:0, d18:1/14:1)* | Lipid |
| X - 11483 | X |
| X - 18899 | X |
| 2-oxoarginine* | Amino Acid |
| X - 14838 | X |
| X - 13684 | X |
| X - 12731 | X |
| propyl 4-hydroxybenzoate sulfate | Xenobiotics |
| X - 24293 | X |
| palmitoyl dihydrosphingomyelin (d18:0/16:0)* | Lipid |
| 1-palmitoyl-2-palmitoleoyl-GPC (16:0/16:1)* | Lipid |
| 1-(1-enyl-stearoyl)-2-arachidonoyl-GPE (P-18:0/20:4)* | Lipid |
| 1-margaroyl-2-oleoyl-GPC (17:0/18:1)* | Lipid |
| X - 24309 | X |
| 1-(1-enyl-stearoyl)-2-arachidonoyl-GPC (P-18:0/20:4) | Lipid |
| 1-(1-enyl-palmitoyl)-2-linoleoyl-GPC (P-16:0/18:2)* | Lipid |
| 1-(1-enyl-stearoyl)-2-linoleoyl-GPC (P-18:0/18:2)* | Lipid |
| 1-(1-enyl-palmitoyl)-2-palmitoyl-GPC (P-16:0/16:0)* | Lipid |
| 1-palmityl-2-arachidonoyl-GPC (O-16:0/20:4)* | Lipid |
| asparagine | Amino Acid |
| 3-aminoisobutyrate | Nucleotide |
| cysteine | Amino Acid |
| betaine | Amino Acid |
| 2-hydroxyoctanoate | Lipid |
| propionylglycine | Lipid |
| 1-methylimidazoleacetate | Amino Acid |
| pseudouridine | Nucleotide |
| N-acetylputrescine | Amino Acid |
| 1-stearoyl-2-arachidonoyl-GPC (18:0/20:4) | Lipid |
| pyroglutamine* | Amino Acid |
| X - 12442 | X |
| X - 13658 | X |
| X - 16580 | X |
| 4-vinylguaiacol sulfate | Xenobiotics |
| carotene diol (2) | Xenobiotics |
| X - 23782 | X |
| choline | Lipid |
| 5-hydroxylysine | Amino Acid |
| phenol sulfate | Amino Acid |
| 2-hydroxy-3-methylvalerate | Amino Acid |
| quinine | Xenobiotics |
| gamma-glutamylvaline | Peptide |
| sphingomyelin (d18:1/20:1, d18:2/20:0)* | Lipid |
| 1-stearoyl-2-arachidonoyl-GPI (18:0/20:4) | Lipid |
| 1-(1-enyl-palmitoyl)-2-myristoyl-GPC (P-16:0/14:0)* | Lipid |

| | |
|---|---|
| serotonin | Amino Acid |
| methionine sulfoxide | Amino Acid |
| taurocholate | Lipid |
| 4-hydroxyhippurate | Xenobiotics |
| 1-oleoyl-GPI (18:1)* | Lipid |
| cysteine sulfinic acid | Amino Acid |
| triamterene | Xenobiotics |
| X - 21442 | X |
| vanillic alcohol sulfate | Amino Acid |
| X - 11315 | X |
| glycerate | Carbohydrate |
| N-acetylaspartate (NAA) | Amino Acid |
| 2-palmitoyl-GPC (16:0)* | Lipid |
| 1-palmitoyl-2-linoleoyl-GPC (16:0/18:2) | Lipid |
| X - 23583 | X |
| 1-(1-enyl-palmitoyl)-2-oleoyl-GPC (P-16:0/18:1)* | Lipid |
| 13-HODE + 9-HODE | Lipid |
| X - 18914 | X |
| X - 11852 | X |
| 1-(1-enyl-stearoyl)-2-docosahexaenoyl-GPE (P-18:0/22:6)* | Lipid |
| uridine | Nucleotide |
| pantothenate | Cofactors and Vitamins |
| phenylacetate | Amino Acid |
| stearoylcarnitine (C18) | Lipid |
| X - 17145 | X |
| 3-carboxy-4-methyl-5-propyl-2-furanpropanoate (CMPF) | Lipid |
| O-sulfo-L-tyrosine | Xenobiotics |
| fructose | Carbohydrate |
| X - 23593 | X |
| guanosine | Nucleotide |
| dodecanedioate | Lipid |
| 5-methyluridine (ribothymidine) | Nucleotide |
| ranitidine | Xenobiotics |
| N-acetyl-1-methylhistidine* | Amino Acid |
| 1-palmityl-2-palmitoyl-GPC (O-16:0/16:0)* | Lipid |
| furosemide | Xenobiotics |
| X - 11905 | X |
| X - 14662 | X |
| X - 13729 | X |
| sphingomyelin (d17:1/16:0, d18:1/15:0, d16:1/17:0)* | Lipid |
| 1-(1-enyl-palmitoyl)-2-docosahexaenoyl-GPE (P-16:0/22:6)* | Lipid |
| fumarate | Energy |
| myristoylcarnitine (C14) | Lipid |
| sphingosine 1-phosphate | Lipid |
| ibuprofen | Xenobiotics |
| glycocholenate sulfate* | Lipid |
| 1-dihomo-linolenoyl-GPC (20:3n3 or 6)* | Lipid |
| 5alpha-androstan-3alpha,17beta-diol disulfate | Lipid |
| X - 12462 | X |
| X - 23587 | X |

| | |
|---|---|
| X - 12101 | X |
| orotate | Nucleotide |
| erythritol | Xenobiotics |
| X - 21353 | X |
| X - 12849 | X |
| cystine | Amino Acid |
| heptanoate (7:0) | Lipid |
| tyramine O-sulfate | Amino Acid |
| imidazole propionate | Amino Acid |
| glycerol 3-phosphate | Lipid |
| X - 24243 | X |
| 1-pentadecanoyl-2-linoleoyl-GPC (15:0/18:2)* | Lipid |
| 1-(1-enyl-palmitoyl)-2-arachidonoyl-GPE (P-16:0/20:4)* | Lipid |
| benzoate | Xenobiotics |
| 3-hydroxylaurate | Lipid |
| pregnanediol-3-glucuronide | Lipid |
| 5-methylthioadenosine (MTA) | Amino Acid |
| estrone 3-sulfate | Lipid |
| stearoyl sphingomyelin (d18:1/18:0) | Lipid |
| 3-indoxyl sulfate | Amino Acid |
| 1-stearoyl-2-linoleoyl-GPC (18:0/18:2)* | Lipid |
| maleate | Lipid |
| N-acetylasparagine | Amino Acid |
| X - 12127 | X |
| naproxen | Xenobiotics |
| sphingomyelin (d18:2/16:0, d18:1/16:1)* | Lipid |
| proline | Amino Acid |
| 3-methylhistidine | Amino Acid |
| 2-oleoylglycerol (18:1) | Lipid |
| theanine | Xenobiotics |
| 1-palmitoyl-2-arachidonoyl-GPE (16:0/20:4)* | Lipid |
| erucate (22:1n9) | Lipid |
| alpha-tocopherol | Cofactors and Vitamins |
| N-acetylleucine | Amino Acid |
| X - 11441 | X |
| X - 21319 | X |
| 1-eicosapentaenoyl-GPE (20:5)* | Lipid |
| 1-(1-enyl-stearoyl)-2-linoleoyl-GPE (P-18:0/18:2)* | Lipid |
| adenine | Nucleotide |
| 4-acetamidophenol | Xenobiotics |
| methylsuccinate | Amino Acid |
| 4-ethylphenylsulfate | Xenobiotics |
| X - 12170 | X |
| X - 12026 | X |
| tryptophan betaine | Amino Acid |
| dihydroferulic acid | Xenobiotics |
| S-allylcysteine | Xenobiotics |
| bilirubin (Z,Z) | Cofactors and Vitamins |
| X - 17677 | X |
| X - 17299 | X |

| | |
|---|---|
| gamma-CEHC | Cofactors and Vitamins |
| X - 15728 | X |
| gulonate* | Cofactors and Vitamins |
| X - 23644 | X |
| threonine | Amino Acid |
| adrenate (22:4n6) | Lipid |
| 1-margaroyl-GPC (17:0) | Lipid |
| X - 11438 | X |
| X - 12740 | X |
| 1-dihomo-linolenylglycerol (20:3) | Lipid |
| taurodeoxycholate | Lipid |
| gamma-glutamylhistidine | Peptide |
| DSGEGDFXAEGGGVR* | Peptide |
| bilirubin (E,E)* | Cofactors and Vitamins |
| erythronate* | Carbohydrate |
| 2-methoxyresorcinol sulfate | Xenobiotics |
| X - 12472 | X |
| ornithine | Amino Acid |
| 3-hydroxydecanoate | Lipid |
| oxypurinol | Xenobiotics |
| 3-hydroxycotinine glucuronide | Xenobiotics |
| X - 02269 | X |
| X - 16570 | X |
| X - 23196 | X |
| kynurenine | Amino Acid |
| 3-phenylpropionate (hydrocinnamate) | Xenobiotics |
| hexanoylcarnitine (C6) | Lipid |
| lidocaine | Xenobiotics |
| X - 16947 | X |
| X - 21849 | X |
| X - 12686 | X |
| hypotaurine | Amino Acid |
| 1-palmitoyl-2-docosahexaenoyl-GPE (16:0/22:6)* | Lipid |
| dimethylarginine (SDMA + ADMA) | Amino Acid |
| prolylglycine | Peptide |
| X - 12798 | X |
| pyrraline | Xenobiotics |
| caprate (10:0) | Lipid |
| thymol sulfate | Xenobiotics |
| acetylcarnitine (C2) | Lipid |
| cis-4-decenoylcarnitine (C10:1) | Lipid |
| 3-methyl-2-oxobutyrate | Amino Acid |
| gluconate | Xenobiotics |
| maltose | Carbohydrate |
| oleoyl ethanolamide | Lipid |
| X - 12306 | X |
| X - 23680 | X |
| 1-palmitoyl-2-arachidonoyl-GPC (16:0/20:4n6) | Lipid |
| 1,3-dimethylurate | Xenobiotics |
| 2-arachidonoyl-GPC (20:4)* | Lipid |

| | |
|---|---|
| hydantoin-5-propionic acid | Amino Acid |
| isovalerate | Amino Acid |
| linoleate (18:2n6) | Lipid |
| saccharin | Xenobiotics |
| homocitrulline | Amino Acid |
| caprylate (8:0) | Lipid |
| ferulic acid 4-sulfate | Xenobiotics |
| X - 18345 | X |
| cystathionine | Amino Acid |
| 1-palmitoyl-2-oleoyl-GPE (16:0/18:1) | Lipid |
| pyridoxate | Cofactors and Vitamins |
| 2-aminobutyrate | Amino Acid |
| X - 17185 | X |
| X - 12844 | X |
| X - 12206 | X |
| X - 17367 | X |
| X - 23590 | X |
| N6,N6,N6-trimethyllysine | Amino Acid |
| 4-hydroxychlorothalonil | Xenobiotics |
| chenodeoxycholate | Lipid |
| palmitoylcarnitine (C16) | Lipid |
| X - 16649 | X |
| N-acetylcitrulline | Amino Acid |
| retinol (Vitamin A) | Cofactors and Vitamins |
| N-acetylglycine | Amino Acid |
| 2-hydroxypalmitate | Lipid |
| hexadecanedioate | Lipid |
| warfarin | Xenobiotics |
| X - 15674 | X |
| isoursodeoxycholate | Lipid |
| myo-inositol | Lipid |
| X - 18249 | X |
| X - 16071 | X |
| urate | Nucleotide |
| indolelactate | Amino Acid |
| X - 21729 | X |
| X - 11564 | X |
| 5,6-dihydrothymine | Nucleotide |
| dimethylglycine | Amino Acid |
| glycocholate | Lipid |
| N-acetylneuraminate | Carbohydrate |
| N-acetylthreonine | Amino Acid |
| glycohyocholate | Lipid |
| X - 21668 | X |
| umbelliferone sulfate | Xenobiotics |
| X - 12730 | X |
| nonadecanoate (19:0) | Lipid |
| hippurate | Xenobiotics |
| tartronate (hydroxymalonate) | Xenobiotics |
| 1-arachidonylglycerol (20:4) | Lipid |

| | |
|---|---|
| lanthionine | Xenobiotics |
| beta-citrylglutamate | Amino Acid |
| 3-hydroxy-3-methylglutarate | Lipid |
| carnitine | Lipid |
| ethylmalonate | Amino Acid |
| X - 11795 | X |
| caffeine | Xenobiotics |
| sphinganine | Lipid |
| salicyluric glucuronide* | Xenobiotics |
| 7-methylguanine | Nucleotide |
| 3-hydroxyquinine | Xenobiotics |
| 5alpha-androstan-3beta,17beta-diol disulfate | Lipid |
| atenolol | Xenobiotics |
| glutamate | Amino Acid |
| gamma-glutamylglutamine | Peptide |
| glycochenodeoxycholate | Lipid |
| 7-methylxanthine | Xenobiotics |
| acisoga | Amino Acid |
| pipecolate | Amino Acid |
| 4-hydroxyphenylacetate | Amino Acid |
| alpha-hydroxyisovalerate | Amino Acid |
| X - 23639 | X |
| palmitic amide | Lipid |
| glutarylcarnitine (C5-DC) | Amino Acid |
| N-trimethyl 5-aminovalerate | Amino Acid |
| X - 11491 | X |
| X - 19455 | X |
| 1,3,7-trimethylurate | Xenobiotics |
| 3-methyl catechol sulfate (1) | Xenobiotics |
| X - 18901 | X |
| kynurenate | Amino Acid |
| valine | Amino Acid |
| glycerol | Lipid |
| pro-hydroxy-pro | Amino Acid |
| X - 12407 | X |
| arabitol/xylitol | Carbohydrate |
| stearate (18:0) | Lipid |
| X - 11540 | X |
| indoleacetylglutamine | Amino Acid |
| X - 12063 | X |
| theophylline | Xenobiotics |
| orotidine | Nucleotide |
| X - 21339 | X |
| X - 11858 | X |
| isocitrate | Energy |
| stachydrine | Xenobiotics |
| sphingomyelin (d18:1/18:1, d18:2/18:0) | Lipid |
| X - 19141 | X |
| glucose | Carbohydrate |
| 3-methoxycatechol sulfate (1) | Xenobiotics |

| | |
|---|---|
| gamma-tocopherol/beta-tocopherol | Cofactors and Vitamins |
| alpha-ketoglutarate | Energy |
| suberate (octanedioate) | Lipid |
| gamma-glutamylleucine | Peptide |
| theobromine | Xenobiotics |
| threonate | Cofactors and Vitamins |
| myristoleate (14:1n5) | Lipid |
| quinolinate | Cofactors and Vitamins |
| 5-dodecenoate (12:1n7) | Lipid |
| choline phosphate | Lipid |
| androstenediol (3beta,17beta) disulfate (1) | Lipid |
| omeprazole | Xenobiotics |
| X - 11452 | X |
| p-cresol-glucuronide* | Amino Acid |
| 1-(1-enyl-palmitoyl)-2-arachidonoyl-GPC (P-16:0/20:4)* | Lipid |
| ursodeoxycholate | Lipid |
| xanthine | Nucleotide |
| X - 17327 | X |
| galactonate | Carbohydrate |
| androsterone sulfate | Lipid |
| 4-methylcatechol sulfate | Xenobiotics |
| X - 11444 | X |
| isobutyrylcarnitine (C4) | Amino Acid |
| 5alpha-pregnan-3beta,20beta-diol monosulfate (1) | Lipid |
| X - 11378 | X |
| X - 11429 | X |
| X - 21752 | X |
| N-acetyltaurine | Amino Acid |
| glucuronate | Carbohydrate |
| 1-linolenoyl-GPC (18:3)* | Lipid |
| 3-(4-hydroxyphenyl)lactate | Amino Acid |
| N-palmitoylglycine | Lipid |
| 1-myristoyl-GPC (14:0) | Lipid |
| deoxycholate | Lipid |
| dihomo-linolenate (20:3n3 or n6) | Lipid |
| o-cresol sulfate | Xenobiotics |
| X - 12411 | X |
| X - 23314 | X |
| 1-methylnicotinamide | Cofactors and Vitamins |
| N-acetyl-beta-alanine | Nucleotide |
| cimetidine | Xenobiotics |
| X - 19183 | X |
| 1-palmitoyl-2-eicosapentaenoyl-GPC (16:0/20:5)* | Lipid |
| 1-(1-enyl-palmitoyl)-2-palmitoleoyl-GPC (P-16:0/16:1)* | Lipid |
| sarcosine | Amino Acid |
| 1-palmitoleoyl-GPC (16:1)* | Lipid |
| 1-methylxanthine | Xenobiotics |
| 1,7-dimethylurate | Xenobiotics |
| X - 21341 | X |
| X - 12013 | X |

| | |
|---|---|
| X - 11522 | X |
| X - 16087 | X |
| tartarate | Xenobiotics |
| oxalate (ethanedioate) | Cofactors and Vitamins |
| oleoylcarnitine (C18:1) | Lipid |
| 2-piperidinone | Xenobiotics |
| X - 11838 | X |
| valerate | Lipid |
| X - 23997 | X |
| arachidonate (20:4n6) | Lipid |
| 1-palmitoylglycerol (16:0) | Lipid |
| X - 17325 | X |
| X - 14314 | X |
| NA | NA |
| androstenediol (3beta,17beta) monosulfate (1) | Lipid |
| 3-hydroxyhippurate | Xenobiotics |
| X - 07765 | X |
| X - 14939 | X |
| X - 21607 | X |
| X - 23588 | X |
| gamma-glutamyltyrosine | Peptide |
| gamma-glutamylisoleucine* | Peptide |
| C-glycosyltryptophan | Amino Acid |
| nicotinamide | Cofactors and Vitamins |
| cortisone | Lipid |
| 2-aminooctanoate | Lipid |
| guanidinoacetate | Amino Acid |
| X - 21258 | X |
| N-methylpipecolate | Xenobiotics |
| X - 12688 | X |
| hydroquinone sulfate | Xenobiotics |
| isobutyrylglycine | Amino Acid |
| eicosanodioate | Lipid |
| carboxyibuprofen | Xenobiotics |
| 21-hydroxypregnenolone disulfate | Lipid |
| X - 22379 | X |
| 4-guanidinobutanoate | Amino Acid |
| 2-arachidonoyl-GPE (20:4)* | Lipid |
| 3-(3-hydroxyphenyl)propionate | Xenobiotics |
| X - 16083 | X |
| sphingomyelin (d18:1/22:1, d18:2/22:0, d16:1/24:1)* | Lipid |
| malate | Energy |
| hypoxanthine | Nucleotide |
| 2-hydroxyhippurate (salicylurate) | Xenobiotics |
| 1,2-dipalmitoyl-GPC (16:0/16:0) | Lipid |
| 4-methyl-2-oxopentanoate | Amino Acid |
| 1-docosahexaenoyl-GPC (22:6)* | Lipid |
| 1-arachidoyl-GPC (20:0) | Lipid |
| 1-(1-enyl-stearoyl)-2-oleoyl-GPE (P-18:0/18:1) | Lipid |
| cotinine | Xenobiotics |

| | |
|---|---|
| N1-methyladenosine | Nucleotide |
| dihomo-linoleate (20:2n6) | Lipid |
| 5alpha-pregnan-3beta,20alpha-diol disulfate | Lipid |
| N1-Methyl-2-pyridone-5-carboxamide | Cofactors and Vitamins |
| X - 11843 | X |
| X - 12544 | X |
| dopamine 3-O-sulfate | Amino Acid |
| glutamine | Amino Acid |
| octanoylcarnitine (C8) | Lipid |
| 1-linolenoylglycerol (18:3) | Lipid |
| tiglylcarnitine (C5:1-DC) | Amino Acid |
| glycerophosphoethanolamine | Lipid |
| succinimide | Xenobiotics |
| X - 15503 | X |
| 2-palmitoleoylglycerol (16:1)* | Lipid |
| phenylpyruvate | Amino Acid |
| sucrose | Carbohydrate |
| thyroxine | Amino Acid |
| ribitol | Carbohydrate |
| glycodeoxycholate | Lipid |
| taurolithocholate 3-sulfate | Lipid |
| phosphate | Energy |
| 1-docosapentaenoyl-GPC (22:5n6)* | Lipid |
| desmethylnaproxen sulfate | Xenobiotics |
| X - 17346 | X |
| 1-palmitoleoylglycerol (16:1)* | Lipid |
| sphingomyelin (d18:2/24:1, d18:1/24:2)* | Lipid |
| histidine | Amino Acid |
| urea | Amino Acid |
| 2-aminoadipate | Amino Acid |
| azelate (nonanedioate) | Lipid |
| 1-palmitoyl-GPA (16:0) | Lipid |
| ethyl glucuronide | Xenobiotics |
| X - 16124 | X |
| X - 15469 | X |
| X - 21803 | X |
| X - 21807 | X |
| X - 12812 | X |
| X - 12221 | X |
| mannose | Carbohydrate |
| X - 23297 | X |
| 1-oleoylglycerol (18:1) | Lipid |
| glycolithocholate | Lipid |
| N-acetylhistidine | Amino Acid |
| N6-carbamoylthreonyladenosine | Nucleotide |
| catechol sulfate | Xenobiotics |
| 4-hydroxycoumarin | Xenobiotics |
| 1-dihomo-linolenoyl-GPE (20:3n3 or 6)* | Lipid |
| X - 12739 | X |
| X - 17269 | X |

| | |
|---|---|
| X - 17337 | X |
| 4-hydroxyphenylacetylglutamine | Peptide |
| 5-oxoproline | Amino Acid |
| phenolphthalein beta-D-glucuronide | Xenobiotics |
| 1-palmitoyl-GPE (16:0) | Lipid |
| succinylcarnitine (C4-DC) | Energy |
| 2-hydroxyibuprofen | Xenobiotics |
| X - 12847 | X |
| X - 17690 | X |
| 1-(1-enyl-stearoyl)-2-oleoyl-GPC (P-18:0/18:1) | Lipid |
| allantoin | Nucleotide |
| 4-acetamidobutanoate | Amino Acid |
| malonate | Lipid |
| cys-gly, oxidized | Amino Acid |
| X - 11787 | X |
| 1-dihomo-linoleoyl-GPC (20:2)* | Lipid |
| O-methylcatechol sulfate | Xenobiotics |
| methyl glucopyranoside (alpha + beta) | Xenobiotics |
| X - 11442 | X |
| X - 21470 | X |
| X - 12846 | X |
| X - 17469 | X |
| X - 12822 | X |
| X - 12410 | X |
| vanillylmandelate (VMA) | Amino Acid |
| beta-hydroxyisovalerate | Amino Acid |
| gentisate | Amino Acid |
| taurocholenate sulfate | Lipid |
| 2,3-dihydroxyisovalerate | Xenobiotics |
| hydrochlorothiazide | Xenobiotics |
| X - 21364 | X |
| X - 17189 | X |
| X - 16944 | X |
| glutarate (pentanedioate) | Lipid |
| N-acetyltyrosine | Amino Acid |
| gamma-glutamyl-epsilon-lysine | Peptide |
| isovalerylglycine | Amino Acid |
| 1-eicosenoyl-GPC (20:1)* | Lipid |
| gamma-glutamylmethionine | Peptide |
| X - 21343 | X |
| X - 17166 | X |
| sulfate* | Xenobiotics |
| X - 21796 | X |
| eugenol sulfate | Xenobiotics |
| 1-margaroyl-2-linoleoyl-GPC (17:0/18:2)* | Lipid |
| 3-hydroxyisobutyrate | Amino Acid |
| methyl indole-3-acetate | Xenobiotics |
| cortisol | Lipid |
| biliverdin | Cofactors and Vitamins |
| 2-hydroxystearate | Lipid |

| | |
|---|---|
| sebacate (decanedioate) | Lipid |
| butyrylcarnitine (C4) | Lipid |
| 1-arachidonoyl-GPI (20:4)* | Lipid |
| 5-acetylamino-6-formylamino-3-methyluracil | Xenobiotics |
| androstenediol (3beta,17beta) monosulfate (2) | Lipid |
| X - 21411 | X |
| X - 12830 | X |
| isoeugenol sulfate | Xenobiotics |
| X - 23739 | X |
| beta-alanine | Nucleotide |
| 3-methoxytyrosine | Amino Acid |
| 1,5-anhydroglucitol (1,5-AG) | Carbohydrate |
| docosapentaenoate (n3 DPA; 22:5n3) | Lipid |
| N-acetyltryptophan | Amino Acid |
| hexanoylglycine | Lipid |
| p-cresol sulfate | Xenobiotics |
| N-acetylserine | Amino Acid |
| chiro-inositol | Lipid |
| 1-eicosapentaenoyl-GPC (20:5)* | Lipid |
| 1-(1-enyl-oleoyl)-GPE (P-18:1)* | Lipid |
| mannitol/sorbitol | Carbohydrate |
| X - 11850 | X |
| X - 12816 | X |
| etiocholanolone glucuronide | Lipid |
| X - 13737 | X |
| X - 12714 | X |
| gamma-glutamyl-alpha-lysine | Peptide |
| N-acetylvaline | Amino Acid |
| taurine | Amino Acid |
| cholate | Lipid |
| 3-methylxanthine | Xenobiotics |
| 1-palmitoyl-GPI (16:0) | Lipid |
| gamma-glutamylglutamate | Peptide |
| 7-alpha-hydroxy-3-oxo-4-cholestenoate (7-Hoca) | Lipid |
| 16a-hydroxy DHEA 3-sulfate | Lipid |
| 2-aminophenol sulfate | Xenobiotics |
| hexanoylglutamine | Lipid |
| X - 16946 | X |
| X - 11372 | X |
| X - 12701 | X |
| tryptophan | Amino Acid |
| uracil | Nucleotide |
| isoleucine | Amino Acid |
| succinate | Energy |
| citrate | Energy |
| 5-hydroxyhexanoate | Lipid |
| caproate (6:0) | Lipid |
| X - 21736 | X |
| X - 22515 | X |
| X - 17357 | X |

| | |
|---|---|
| methyl-4-hydroxybenzoate sulfate | Xenobiotics |
| X - 23369 | X |
| 1-(1-enyl-stearoyl)-2-docosahexaenoyl-GPC (P-18:0/22:6)* | Lipid |
| aspartate | Amino Acid |
| N-acetylalanine | Amino Acid |
| N-acetylmethionine | Amino Acid |
| 3-ureidopropionate | Nucleotide |
| N-acetylglutamate | Amino Acid |
| indoleacetate | Amino Acid |
| 1-methylhistidine | Amino Acid |
| glycolithocholate sulfate* | Lipid |
| N2,N2-dimethylguanosine | Nucleotide |
| 4-allylphenol sulfate | Xenobiotics |
| N-methylproline | Amino Acid |
| pregnenolone sulfate | Lipid |
| 6-oxopiperidine-2-carboxylate | Amino Acid |
| 3-hydroxybutyrylcarnitine (1) | Lipid |
| X - 21441 | X |
| X - 11299 | X |
| X - 12230 | X |
| X - 14658 | X |
| X - 16935 | X |
| X - 21792 | X |
| X - 12543 | X |
| X - 23729 | X |
| tyrosine | Amino Acid |
| lysine | Amino Acid |
| phenyllactate (PLA) | Amino Acid |
| 1-palmitoyl-GPC (16:0) | Lipid |
| N6-acetyllysine | Amino Acid |
| 5alpha-androstan-3alpha,17beta-diol monosulfate (1) | Lipid |
| 5alpha-pregnan-3beta,20alpha-diol monosulfate (2) | Lipid |
| 1-(1-enyl-palmitoyl)-GPE (P-16:0)* | Lipid |
| X - 11530 | X |
| X - 12007 | X |
| X - 21626 | X |
| linoleoylcholine* | Lipid |
| argininate* | Amino Acid |
| X - 13431 | X |
| arabonate/xylonate | Carbohydrate |
| dopamine 4-sulfate | Amino Acid |
| 2-acetamidophenol sulfate | Xenobiotics |
| 1-stearoyl-2-linoleoyl-GPE (18:0/18:2)* | Lipid |
| homoarginine | Amino Acid |
| phenylacetylglutamine | Peptide |
| cysteinylglycine | Amino Acid |
| 4-acetaminophen sulfate | Xenobiotics |
| 1-(1-enyl-stearoyl)-GPE (P-18:0)* | Lipid |
| N-delta-acetylornithine | Amino Acid |
| 2-aminoheptanoate | Lipid |

| | |
|---|---|
| 1-docosahexaenoyl-GPE (22:6)* | Lipid |
| N-acetylalliin | Xenobiotics |
| 2-palmitoyl-GPE (16:0)* | Lipid |
| desmethylnaproxen | Xenobiotics |
| X - 13866 | X |
| X - 01911 | X |
| 3-methylglutarylcarnitine (2) | Amino Acid |
| X - 14056 | X |
| 1,2,3-benzenetriol sulfate (1) | Xenobiotics |
| 1-(1-enyl-palmitoyl)-2-oleoyl-GPE (P-16:0/18:1)* | Lipid |
| phenylalanine | Amino Acid |
| salicylate | Xenobiotics |
| 3-hydroxy-2-ethylpropionate | Amino Acid |
| gamma-glutamylphenylalanine | Peptide |
| decanoylcarnitine (C10) | Lipid |
| 3,7-dimethylurate | Xenobiotics |
| isovalerylcarnitine (C5) | Amino Acid |
| 2-linoleoyl-GPE (18:2)* | Lipid |
| 5alpha-androstan-3beta,17alpha-diol disulfate | Lipid |
| glycoursodeoxycholate | Lipid |
| 2-stearoyl-GPE (18:0)* | Lipid |
| 2-hydroxydecanoate | Lipid |
| X - 15497 | X |
| X - 11470 | X |
| X - 11538 | X |
| X - 11880 | X |
| X - 12216 | X |
| X - 12126 | X |
| X - 12680 | X |
| X - 13844 | X |
| X - 17685 | X |
| 2-oleoyl-GPC (18:1)* | Lipid |
| N-acetylkynurenine (2) | Amino Acid |
| pyruvate | Carbohydrate |
| 1-stearoyl-2-linoleoyl-GPI (18:0/18:2) | Lipid |
| 1-(1-enyl-palmitoyl)-2-docosahexaenoyl-GPC (P-16:0/22:6)* | Lipid |
| arachidate (20:0) | Lipid |
| serine | Amino Acid |
| N-formylmethionine | Amino Acid |
| 4-acetamidophenylglucuronide | Xenobiotics |
| glycerophosphorylcholine (GPC) | Lipid |
| 1-linoleoylglycerol (18:2) | Lipid |
| 2-hydroxyacetaminophen sulfate* | Xenobiotics |
| 3-(cystein-S-yl)acetaminophen* | Xenobiotics |
| 1-docosahexaenoylglycerol (22:6) | Lipid |
| cinnamoylglycine | Xenobiotics |
| 3-(3-hydroxyphenyl)propionate sulfate | Xenobiotics |
| X - 11440 | X |
| X - 12231 | X |
| X - 12329 | X |

| | |
|---|---|
| X - 12100 | X |
| X - 12738 | X |
| X - 23637 | X |
| X - 23649 | X |
| 1-palmitoyl-2-arachidonoyl-GPI (16:0/20:4)* | Lipid |
| arginine | Amino Acid |
| 3-methyl-2-oxovalerate | Amino Acid |
| paraxanthine | Xenobiotics |
| taurochenodeoxycholate | Lipid |
| 1-stearoyl-GPI (18:0) | Lipid |
| N-(2-furoyl)glycine | Xenobiotics |
| propionylcarnitine (C3) | Lipid |
| gamma-glutamylthreonine | Peptide |
| stearidonate (18:4n3) | Lipid |
| 1-methylurate | Xenobiotics |
| 1-arachidonoyl-GPE (20:4n6)* | Lipid |
| 1-oleoyl-GPE (18:1) | Lipid |
| 1-linoleoyl-GPI (18:2)* | Lipid |
| androstenediol (3beta,17beta) disulfate (2) | Lipid |
| cotinine N-oxide | Xenobiotics |
| X - 15486 | X |
| X - 11478 | X |
| X - 12261 | X |
| X - 17351 | X |
| sphingomyelin (d18:1/20:0, d16:1/22:0)* | Lipid |

**lected times in the bootstrapping process**

| sub_pathway | selected times |
|---|---|
| Glycine, Serine and Threonine Metabolism | 100 |
| Sterol | 100 |
| Creatine Metabolism | 100 |
| Alanine and Aspartate Metabolism | 100 |
| Tyrosine Metabolism | 100 |
| Urea cycle; Arginine and Proline Metabolism | 100 |
| Histidine Metabolism | 100 |
| Sphingolipid Metabolism | 100 |
| Lysoplasmalogen | 100 |
| Leucine, Isoleucine and Valine Metabolism | 100 |
| Creatine Metabolism | 100 |
| Urea cycle; Arginine and Proline Metabolism | 100 |
| Monoacylglycerol | 100 |
| Androgenic Steroids | 100 |
| Food Component/Plant | 100 |
| Lysophospholipid | 100 |
| Glutamate Metabolism | 100 |
| Phenylalanine Metabolism | 100 |
| Long Chain Fatty Acid | 100 |
| Monoacylglycerol | 100 |
| Benzoate Metabolism | 100 |
| Carnitine Metabolism | 100 |
| Androgenic Steroids | 100 |
| Lysophospholipid | 100 |
| Lysophospholipid | 100 |
| Food Component/Plant | 100 |
| Fatty Acid, Branched | 100 |
| Methionine, Cysteine, SAM and Taurine Metabolism | 100 |
| Phospholipid Metabolism | 100 |
| Phosphatidylethanolamine (PE) | 100 |
| Sphingolipid Metabolism | 100 |
| Drug | 100 |
| Dipeptide Derivative | 100 |
| Drug | 100 |
| Polyunsaturated Fatty Acid (n3 and n6) | 100 |
| Ceramides | 100 |
| Methionine, Cysteine, SAM and Taurine Metabolism | 100 |
| X | 100 |
| Lysophospholipid | 100 |
| X | 100 |
| X | 100 |
| X | 100 |
| X | 100 |
| X | 100 |
| X | 100 |
| X | 100 |
| X | 100 |
| X | 100 |
| X | 100 |

| | |
|---|---|
| X | 100 |
| X | 100 |
| X | 100 |
| X | 100 |
| X | 100 |
| Sphingolipid Metabolism | 100 |
| X | 100 |
| X | 100 |
| Urea cycle; Arginine and Proline Metabolism | 100 |
| X | 100 |
| X | 100 |
| X | 100 |
| Benzoate Metabolism | 100 |
| X | 100 |
| Sphingolipid Metabolism | 100 |
| Phosphatidylcholine (PC) | 100 |
| Plasmalogen | 100 |
| Phosphatidylcholine (PC) | 100 |
| X | 100 |
| Plasmalogen | 100 |
| Plasmalogen | 100 |
| Plasmalogen | 100 |
| Plasmalogen | 100 |
| Plasmalogen | 100 |
| Alanine and Aspartate Metabolism | 99 |
| Pyrimidine Metabolism, Thymine containing | 99 |
| Methionine, Cysteine, SAM and Taurine Metabolism | 99 |
| Glycine, Serine and Threonine Metabolism | 99 |
| Fatty Acid, Monohydroxy | 99 |
| Fatty Acid Metabolism (also BCAA Metabolism) | 99 |
| Histidine Metabolism | 99 |
| Pyrimidine Metabolism, Uracil containing | 99 |
| Polyamine Metabolism | 99 |
| Phosphatidylcholine (PC) | 99 |
| Glutamate Metabolism | 99 |
| X | 99 |
| X | 99 |
| X | 99 |
| Food Component/Plant | 99 |
| Food Component/Plant | 99 |
| X | 99 |
| Phospholipid Metabolism | 98 |
| Lysine Metabolism | 98 |
| Tyrosine Metabolism | 98 |
| Leucine, Isoleucine and Valine Metabolism | 98 |
| Drug | 98 |
| Gamma-glutamyl Amino Acid | 98 |
| Sphingolipid Metabolism | 98 |
| Phosphatidylinositol (PI) | 98 |
| Plasmalogen | 98 |

| | |
|---|---|
| Tryptophan Metabolism | 97 |
| Methionine, Cysteine, SAM and Taurine Metabolism | 97 |
| Primary Bile Acid Metabolism | 97 |
| Benzoate Metabolism | 97 |
| Lysophospholipid | 97 |
| Methionine, Cysteine, SAM and Taurine Metabolism | 97 |
| Drug | 97 |
| X | 97 |
| Tyrosine Metabolism | 97 |
| X | 97 |
| Glycolysis, Gluconeogenesis, and Pyruvate Metabolism | 96 |
| Alanine and Aspartate Metabolism | 96 |
| Lysophospholipid | 96 |
| Phosphatidylcholine (PC) | 96 |
| X | 96 |
| Plasmalogen | 96 |
| Fatty Acid, Monohydroxy | 95 |
| X | 95 |
| X | 95 |
| Plasmalogen | 95 |
| Pyrimidine Metabolism, Uracil containing | 94 |
| Pantothenate and CoA Metabolism | 94 |
| Phenylalanine Metabolism | 94 |
| Fatty Acid Metabolism(Acyl Carnitine) | 94 |
| X | 94 |
| Fatty Acid, Dicarboxylate | 93 |
| Chemical | 93 |
| Fructose, Mannose and Galactose Metabolism | 93 |
| X | 93 |
| Purine Metabolism, Guanine containing | 92 |
| Fatty Acid, Dicarboxylate | 92 |
| Pyrimidine Metabolism, Uracil containing | 92 |
| Drug | 92 |
| Histidine Metabolism | 92 |
| Plasmalogen | 92 |
| Drug | 91 |
| X | 91 |
| X | 91 |
| X | 91 |
| Sphingolipid Metabolism | 91 |
| Plasmalogen | 91 |
| TCA Cycle | 90 |
| Fatty Acid Metabolism(Acyl Carnitine) | 90 |
| Sphingolipid Metabolism | 90 |
| Drug | 89 |
| Secondary Bile Acid Metabolism | 89 |
| Lysophospholipid | 89 |
| Androgenic Steroids | 89 |
| X | 89 |
| X | 89 |

| | |
|---|---|
| X | 88 |
| Pyrimidine Metabolism, Orotate containing | 87 |
| Food Component/Plant | 87 |
| X | 87 |
| X | 87 |
| Methionine, Cysteine, SAM and Taurine Metabolism | 86 |
| Medium Chain Fatty Acid | 86 |
| Tyrosine Metabolism | 86 |
| Histidine Metabolism | 85 |
| Glycerolipid Metabolism | 85 |
| X | 85 |
| Phosphatidylcholine (PC) | 85 |
| Plasmalogen | 85 |
| Benzoate Metabolism | 84 |
| Fatty Acid, Monohydroxy | 84 |
| Progestin Steroids | 84 |
| Polyamine Metabolism | 83 |
| Estrogenic Steroids | 83 |
| Sphingolipid Metabolism | 83 |
| Tryptophan Metabolism | 83 |
| Phosphatidylcholine (PC) | 83 |
| Fatty Acid, Dicarboxylate | 82 |
| Alanine and Aspartate Metabolism | 82 |
| X | 82 |
| Drug | 81 |
| Sphingolipid Metabolism | 81 |
| Urea cycle; Arginine and Proline Metabolism | 80 |
| Histidine Metabolism | 80 |
| Monoacylglycerol | 80 |
| Food Component/Plant | 80 |
| Phosphatidylethanolamine (PE) | 80 |
| Long Chain Fatty Acid | 79 |
| Tocopherol Metabolism | 79 |
| Leucine, Isoleucine and Valine Metabolism | 79 |
| X | 79 |
| X | 79 |
| Lysophospholipid | 79 |
| Plasmalogen | 79 |
| Purine Metabolism, Adenine containing | 78 |
| Drug | 78 |
| Leucine, Isoleucine and Valine Metabolism | 78 |
| Benzoate Metabolism | 78 |
| X | 78 |
| X | 78 |
| Tryptophan Metabolism | 77 |
| Food Component/Plant | 77 |
| Food Component/Plant | 77 |
| Hemoglobin and Porphyrin Metabolism | 77 |
| X | 77 |
| X | 76 |

| | |
|---|---|
| Tocopherol Metabolism | 76 |
| X | 76 |
| Ascorbate and Aldarate Metabolism | 76 |
| X | 76 |
| Glycine, Serine and Threonine Metabolism | 75 |
| Polyunsaturated Fatty Acid (n3 and n6) | 75 |
| Lysophospholipid | 75 |
| X | 75 |
| X | 75 |
| Monoacylglycerol | 75 |
| Secondary Bile Acid Metabolism | 73 |
| Gamma-glutamyl Amino Acid | 73 |
| Fibrinogen Cleavage Peptide | 73 |
| Hemoglobin and Porphyrin Metabolism | 73 |
| Aminosugar Metabolism | 73 |
| Chemical | 73 |
| X | 73 |
| Urea cycle; Arginine and Proline Metabolism | 71 |
| Fatty Acid, Monohydroxy | 71 |
| Drug | 71 |
| Tobacco Metabolite | 71 |
| X | 71 |
| X | 71 |
| X | 71 |
| Tryptophan Metabolism | 70 |
| Benzoate Metabolism | 70 |
| Fatty Acid Metabolism(Acyl Carnitine) | 70 |
| Drug | 70 |
| X | 70 |
| X | 70 |
| X | 70 |
| Methionine, Cysteine, SAM and Taurine Metabolism | 69 |
| Phosphatidylethanolamine (PE) | 69 |
| Urea cycle; Arginine and Proline Metabolism | 68 |
| Dipeptide | 68 |
| X | 68 |
| Food Component/Plant | 68 |
| Medium Chain Fatty Acid | 67 |
| Food Component/Plant | 67 |
| Fatty Acid Metabolism(Acyl Carnitine) | 66 |
| Fatty Acid Metabolism(Acyl Carnitine) | 66 |
| Leucine, Isoleucine and Valine Metabolism | 66 |
| Food Component/Plant | 65 |
| Glycogen Metabolism | 65 |
| Endocannabinoid | 65 |
| X | 65 |
| X | 65 |
| Phosphatidylcholine (PC) | 65 |
| Xanthine Metabolism | 64 |
| Lysophospholipid | 64 |

| | |
|---|---|
| Histidine Metabolism | 64 |
| Leucine, Isoleucine and Valine Metabolism | 64 |
| Polyunsaturated Fatty Acid (n3 and n6) | 63 |
| Food Component/Plant | 63 |
| Urea cycle; Arginine and Proline Metabolism | 63 |
| Medium Chain Fatty Acid | 63 |
| Food Component/Plant | 63 |
| X | 63 |
| Methionine, Cysteine, SAM and Taurine Metabolism | 62 |
| Phosphatidylethanolamine (PE) | 62 |
| Vitamin B6 Metabolism | 62 |
| Glutathione Metabolism | 62 |
| X | 62 |
| X | 62 |
| X | 61 |
| X | 61 |
| X | 61 |
| Lysine Metabolism | 60 |
| Chemical | 60 |
| Primary Bile Acid Metabolism | 59 |
| Fatty Acid Metabolism(Acyl Carnitine) | 59 |
| X | 59 |
| Urea cycle; Arginine and Proline Metabolism | 59 |
| Vitamin A Metabolism | 58 |
| Glycine, Serine and Threonine Metabolism | 58 |
| Fatty Acid, Monohydroxy | 58 |
| Fatty Acid, Dicarboxylate | 58 |
| Drug | 58 |
| X | 58 |
| Secondary Bile Acid Metabolism | 58 |
| Inositol Metabolism | 57 |
| X | 57 |
| X | 57 |
| Purine Metabolism, (Hypo)Xanthine/Inosine containing | 56 |
| Tryptophan Metabolism | 56 |
| X | 56 |
| X | 55 |
| Pyrimidine Metabolism, Thymine containing | 54 |
| Glycine, Serine and Threonine Metabolism | 54 |
| Primary Bile Acid Metabolism | 54 |
| Aminosugar Metabolism | 54 |
| Glycine, Serine and Threonine Metabolism | 54 |
| Secondary Bile Acid Metabolism | 54 |
| X | 53 |
| Food Component/Plant | 53 |
| X | 52 |
| Long Chain Fatty Acid | 51 |
| Benzoate Metabolism | 51 |
| Bacterial/Fungal | 51 |
| Monoacylglycerol | 51 |

| | |
|---|---|
| Chemical | 51 |
| Glutamate Metabolism | 51 |
| Mevalonate Metabolism | 50 |
| Carnitine Metabolism | 50 |
| Leucine, Isoleucine and Valine Metabolism | 50 |
| X | 50 |
| Xanthine Metabolism | 49 |
| Sphingolipid Metabolism | 49 |
| Drug | 49 |
| Purine Metabolism, Guanine containing | 49 |
| Drug | 49 |
| Androgenic Steroids | 48 |
| Drug | 48 |
| Glutamate Metabolism | 47 |
| Gamma-glutamyl Amino Acid | 47 |
| Primary Bile Acid Metabolism | 47 |
| Xanthine Metabolism | 47 |
| Polyamine Metabolism | 47 |
| Lysine Metabolism | 46 |
| Phenylalanine Metabolism | 45 |
| Leucine, Isoleucine and Valine Metabolism | 45 |
| X | 45 |
| Fatty Acid, Amide | 44 |
| Lysine Metabolism | 44 |
| Lysine Metabolism | 44 |
| X | 44 |
| X | 44 |
| Xanthine Metabolism | 43 |
| Benzoate Metabolism | 43 |
| X | 43 |
| Tryptophan Metabolism | 42 |
| Leucine, Isoleucine and Valine Metabolism | 42 |
| Glycerolipid Metabolism | 42 |
| Urea cycle; Arginine and Proline Metabolism | 42 |
| X | 42 |
| Pentose Metabolism | 42 |
| Long Chain Fatty Acid | 41 |
| X | 41 |
| Tryptophan Metabolism | 41 |
| X | 41 |
| Xanthine Metabolism | 40 |
| Pyrimidine Metabolism, Orotate containing | 40 |
| X | 40 |
| X | 40 |
| TCA Cycle | 39 |
| Food Component/Plant | 39 |
| Sphingolipid Metabolism | 39 |
| X | 39 |
| Glycolysis, Gluconeogenesis, and Pyruvate Metabolism | 39 |
| Benzoate Metabolism | 39 |

| | |
|---|---|
| Tocopherol Metabolism | 39 |
| TCA Cycle | 38 |
| Fatty Acid, Dicarboxylate | 38 |
| Gamma-glutamyl Amino Acid | 38 |
| Xanthine Metabolism | 38 |
| Ascorbate and Aldarate Metabolism | 38 |
| Long Chain Fatty Acid | 38 |
| Nicotinate and Nicotinamide Metabolism | 37 |
| Medium Chain Fatty Acid | 37 |
| Phospholipid Metabolism | 37 |
| Androgenic Steroids | 37 |
| Drug | 37 |
| X | 37 |
| Tyrosine Metabolism | 37 |
| Plasmalogen | 37 |
| Secondary Bile Acid Metabolism | 36 |
| Purine Metabolism, (Hypo)Xanthine/Inosine containing | 36 |
| X | 36 |
| Fructose, Mannose and Galactose Metabolism | 35 |
| Androgenic Steroids | 35 |
| Benzoate Metabolism | 35 |
| X | 35 |
| Leucine, Isoleucine and Valine Metabolism | 34 |
| Progestin Steroids | 34 |
| X | 34 |
| X | 34 |
| X | 34 |
| Methionine, Cysteine, SAM and Taurine Metabolism | 34 |
| Aminosugar Metabolism | 33 |
| Lysophospholipid | 33 |
| Tyrosine Metabolism | 32 |
| Fatty Acid Metabolism(Acyl Glycine) | 32 |
| Lysophospholipid | 32 |
| Secondary Bile Acid Metabolism | 31 |
| Polyunsaturated Fatty Acid (n3 and n6) | 31 |
| Benzoate Metabolism | 31 |
| X | 31 |
| X | 31 |
| Nicotinate and Nicotinamide Metabolism | 30 |
| Pyrimidine Metabolism, Uracil containing | 30 |
| Drug | 30 |
| X | 30 |
| Phosphatidylcholine (PC) | 30 |
| Plasmalogen | 30 |
| Glycine, Serine and Threonine Metabolism | 29 |
| Lysophospholipid | 29 |
| Xanthine Metabolism | 29 |
| Xanthine Metabolism | 29 |
| X | 29 |
| X | 29 |

| | |
|---|---|
| X | 29 |
| X | 29 |
| Food Component/Plant | 28 |
| Ascorbate and Aldarate Metabolism | 28 |
| Fatty Acid Metabolism(Acyl Carnitine) | 28 |
| Food Component/Plant | 28 |
| X | 28 |
| Short Chain Fatty Acid | 27 |
| X | 27 |
| Polyunsaturated Fatty Acid (n3 and n6) | 26 |
| Monoacylglycerol | 26 |
| X | 26 |
| X | 26 |
| NA | 26 |
| Androgenic Steroids | 25 |
| Benzoate Metabolism | 25 |
| X | 25 |
| X | 25 |
| X | 25 |
| X | 25 |
| Gamma-glutamyl Amino Acid | 24 |
| Gamma-glutamyl Amino Acid | 24 |
| Tryptophan Metabolism | 24 |
| Nicotinate and Nicotinamide Metabolism | 23 |
| Corticosteroids | 23 |
| Fatty Acid, Amino | 23 |
| Creatine Metabolism | 23 |
| X | 23 |
| Bacterial/Fungal | 23 |
| X | 23 |
| Drug | 22 |
| Leucine, Isoleucine and Valine Metabolism | 22 |
| Fatty Acid, Dicarboxylate | 22 |
| Drug | 22 |
| Pregnenolone Steroids | 22 |
| X | 22 |
| Guanidino and Acetamido Metabolism | 21 |
| Lysophospholipid | 21 |
| Benzoate Metabolism | 21 |
| X | 21 |
| Sphingolipid Metabolism | 21 |
| TCA Cycle | 20 |
| Purine Metabolism, (Hypo)Xanthine/Inosine containing | 20 |
| Benzoate Metabolism | 20 |
| Phosphatidylcholine (PC) | 20 |
| Leucine, Isoleucine and Valine Metabolism | 20 |
| Lysophospholipid | 20 |
| Lysophospholipid | 20 |
| Plasmalogen | 20 |
| Tobacco Metabolite | 19 |

| | |
|---|---|
| Purine Metabolism, Adenine containing | 19 |
| Polyunsaturated Fatty Acid (n3 and n6) | 19 |
| Progestin Steroids | 19 |
| Nicotinate and Nicotinamide Metabolism | 19 |
| X | 19 |
| X | 19 |
| Tyrosine Metabolism | 19 |
| Glutamate Metabolism | 18 |
| Fatty Acid Metabolism(Acyl Carnitine) | 18 |
| Monoacylglycerol | 18 |
| Leucine, Isoleucine and Valine Metabolism | 18 |
| Phospholipid Metabolism | 18 |
| Chemical | 18 |
| X | 18 |
| Monoacylglycerol | 18 |
| Phenylalanine Metabolism | 17 |
| Disaccharides and Oligosaccharides | 17 |
| Tyrosine Metabolism | 17 |
| Pentose Metabolism | 17 |
| Secondary Bile Acid Metabolism | 17 |
| Secondary Bile Acid Metabolism | 17 |
| Oxidative Phosphorylation | 17 |
| Lysophospholipid | 17 |
| Drug | 17 |
| X | 17 |
| Monoacylglycerol | 17 |
| Sphingolipid Metabolism | 17 |
| Histidine Metabolism | 16 |
| Urea cycle; Arginine and Proline Metabolism | 16 |
| Lysine Metabolism | 16 |
| Fatty Acid, Dicarboxylate | 16 |
| Lysophospholipid | 16 |
| Chemical | 16 |
| X | 16 |
| X | 16 |
| X | 16 |
| X | 16 |
| X | 16 |
| X | 16 |
| Fructose, Mannose and Galactose Metabolism | 16 |
| X | 16 |
| Monoacylglycerol | 15 |
| Secondary Bile Acid Metabolism | 15 |
| Histidine Metabolism | 15 |
| Purine Metabolism, Adenine containing | 15 |
| Benzoate Metabolism | 15 |
| Drug | 15 |
| Lysophospholipid | 15 |
| X | 15 |
| X | 15 |

| | |
|---|---|
| X | 15 |
| Acetylated Peptides | 15 |
| Glutathione Metabolism | 14 |
| Chemical | 14 |
| Lysophospholipid | 14 |
| TCA Cycle | 14 |
| Drug | 14 |
| X | 14 |
| X | 14 |
| Plasmalogen | 14 |
| Purine Metabolism, (Hypo)Xanthine/Inosine containing | 13 |
| Polyamine Metabolism | 13 |
| Fatty Acid Synthesis | 13 |
| Glutathione Metabolism | 13 |
| X | 13 |
| Lysophospholipid | 13 |
| Benzoate Metabolism | 13 |
| Food Component/Plant | 13 |
| X | 13 |
| X | 13 |
| X | 13 |
| X | 13 |
| X | 13 |
| X | 13 |
| Tyrosine Metabolism | 12 |
| Leucine, Isoleucine and Valine Metabolism | 12 |
| Tyrosine Metabolism | 12 |
| Secondary Bile Acid Metabolism | 12 |
| Food Component/Plant | 12 |
| Drug | 12 |
| X | 12 |
| X | 12 |
| X | 12 |
| Fatty Acid, Dicarboxylate | 11 |
| Tyrosine Metabolism | 11 |
| Gamma-glutamyl Amino Acid | 11 |
| Leucine, Isoleucine and Valine Metabolism | 11 |
| Lysophospholipid | 11 |
| Gamma-glutamyl Amino Acid | 11 |
| X | 11 |
| X | 11 |
| Chemical | 11 |
| X | 11 |
| Food Component/Plant | 11 |
| Phosphatidylcholine (PC) | 11 |
| Leucine, Isoleucine and Valine Metabolism | 10 |
| Food Component/Plant | 10 |
| Corticosteroids | 10 |
| Hemoglobin and Porphyrin Metabolism | 10 |
| Fatty Acid, Monohydroxy | 10 |

| | |
|---|---|
| Fatty Acid, Dicarboxylate | 10 |
| Fatty Acid Metabolism (also BCAA Metabolism) | 10 |
| Lysophospholipid | 10 |
| Xanthine Metabolism | 10 |
| Androgenic Steroids | 10 |
| X | 10 |
| X | 10 |
| Food Component/Plant | 10 |
| X | 10 |
| Pyrimidine Metabolism, Uracil containing | 9 |
| Tyrosine Metabolism | 9 |
| Glycolysis, Gluconeogenesis, and Pyruvate Metabolism | 9 |
| Polyunsaturated Fatty Acid (n3 and n6) | 9 |
| Tryptophan Metabolism | 9 |
| Fatty Acid Metabolism(Acyl Glycine) | 9 |
| Benzoate Metabolism | 9 |
| Glycine, Serine and Threonine Metabolism | 9 |
| Inositol Metabolism | 9 |
| Lysophospholipid | 9 |
| Lysoplasmalogen | 9 |
| Fructose, Mannose and Galactose Metabolism | 9 |
| X | 9 |
| X | 9 |
| Androgenic Steroids | 9 |
| X | 9 |
| X | 9 |
| Gamma-glutamyl Amino Acid | 9 |
| Leucine, Isoleucine and Valine Metabolism | 8 |
| Methionine, Cysteine, SAM and Taurine Metabolism | 8 |
| Primary Bile Acid Metabolism | 8 |
| Xanthine Metabolism | 8 |
| Lysophospholipid | 8 |
| Gamma-glutamyl Amino Acid | 8 |
| Sterol | 8 |
| Androgenic Steroids | 8 |
| Chemical | 8 |
| Fatty Acid Metabolism (Acyl Glutamine) | 8 |
| X | 8 |
| X | 8 |
| X | 8 |
| Tryptophan Metabolism | 7 |
| Pyrimidine Metabolism, Uracil containing | 7 |
| Leucine, Isoleucine and Valine Metabolism | 7 |
| TCA Cycle | 7 |
| TCA Cycle | 7 |
| Fatty Acid, Monohydroxy | 7 |
| Medium Chain Fatty Acid | 7 |
| X | 7 |
| X | 7 |
| X | 7 |

| | |
|---|---|
| Benzoate Metabolism | 7 |
| X | 7 |
| Plasmalogen | 7 |
| Alanine and Aspartate Metabolism | 6 |
| Alanine and Aspartate Metabolism | 6 |
| Methionine, Cysteine, SAM and Taurine Metabolism | 6 |
| Pyrimidine Metabolism, Uracil containing | 6 |
| Glutamate Metabolism | 6 |
| Tryptophan Metabolism | 6 |
| Histidine Metabolism | 6 |
| Secondary Bile Acid Metabolism | 6 |
| Purine Metabolism, Guanine containing | 6 |
| Food Component/Plant | 6 |
| Urea cycle; Arginine and Proline Metabolism | 6 |
| Pregnenolone Steroids | 6 |
| Lysine Metabolism | 6 |
| Fatty Acid Metabolism(Acyl Carnitine) | 6 |
| X | 6 |
| X | 6 |
| X | 6 |
| X | 6 |
| X | 6 |
| X | 6 |
| X | 6 |
| X | 6 |
| Tyrosine Metabolism | 5 |
| Lysine Metabolism | 5 |
| Phenylalanine Metabolism | 5 |
| Lysophospholipid | 5 |
| Lysine Metabolism | 5 |
| Androgenic Steroids | 5 |
| Progestin Steroids | 5 |
| Lysoplasmalogen | 5 |
| X | 5 |
| X | 5 |
| X | 5 |
| Fatty Acid Metabolism (Acyl Choline) | 5 |
| Urea cycle; Arginine and Proline Metabolism | 5 |
| X | 5 |
| Pentose Metabolism | 5 |
| Tyrosine Metabolism | 5 |
| Drug | 5 |
| Phosphatidylethanolamine (PE) | 5 |
| Urea cycle; Arginine and Proline Metabolism | 4 |
| Acetylated Peptides | 4 |
| Glutathione Metabolism | 4 |
| Drug | 4 |
| Lysoplasmalogen | 4 |
| Urea cycle; Arginine and Proline Metabolism | 4 |
| Fatty Acid, Amino | 4 |

| | |
|---|---|
| Lysophospholipid | 4 |
| Food Component/Plant | 4 |
| Lysophospholipid | 4 |
| Drug | 4 |
| X | 4 |
| X | 4 |
| Leucine, Isoleucine and Valine Metabolism | 4 |
| X | 4 |
| Chemical | 4 |
| Plasmalogen | 4 |
| Phenylalanine Metabolism | 3 |
| Drug | 3 |
| Leucine, Isoleucine and Valine Metabolism | 3 |
| Gamma-glutamyl Amino Acid | 3 |
| Fatty Acid Metabolism(Acyl Carnitine) | 3 |
| Xanthine Metabolism | 3 |
| Leucine, Isoleucine and Valine Metabolism | 3 |
| Lysophospholipid | 3 |
| Androgenic Steroids | 3 |
| Secondary Bile Acid Metabolism | 3 |
| Lysophospholipid | 3 |
| Fatty Acid, Monohydroxy | 3 |
| X | 3 |
| X | 3 |
| X | 3 |
| X | 3 |
| X | 3 |
| X | 3 |
| X | 3 |
| X | 3 |
| X | 3 |
| Lysophospholipid | 3 |
| Tryptophan Metabolism | 3 |
| Glycolysis, Gluconeogenesis, and Pyruvate Metabolism | 3 |
| Phosphatidylinositol (PI) | 3 |
| Plasmalogen | 3 |
| Long Chain Fatty Acid | 2 |
| Glycine, Serine and Threonine Metabolism | 2 |
| Methionine, Cysteine, SAM and Taurine Metabolism | 2 |
| Drug | 2 |
| Phospholipid Metabolism | 2 |
| Monoacylglycerol | 2 |
| Drug | 2 |
| Drug | 2 |
| Monoacylglycerol | 2 |
| Food Component/Plant | 2 |
| Benzoate Metabolism | 2 |
| X | 2 |
| X | 2 |
| X | 2 |

| | |
|---|---|
| X | 2 |
| X | 2 |
| X | 2 |
| X | 2 |
| Phosphatidylinositol (PI) | 2 |
| Urea cycle; Arginine and Proline Metabolism | 1 |
| Leucine, Isoleucine and Valine Metabolism | 1 |
| Xanthine Metabolism | 1 |
| Primary Bile Acid Metabolism | 1 |
| Lysophospholipid | 1 |
| Food Component/Plant | 1 |
| Fatty Acid Metabolism (also BCAA Metabolism) | 1 |
| Gamma-glutamyl Amino Acid | 1 |
| Polyunsaturated Fatty Acid (n3 and n6) | 1 |
| Xanthine Metabolism | 1 |
| Lysophospholipid | 1 |
| Lysophospholipid | 1 |
| Lysophospholipid | 1 |
| Androgenic Steroids | 1 |
| Tobacco Metabolite | 1 |
| X | 1 |
| X | 1 |
| X | 1 |
| X | 1 |
| Sphingolipid Metabolism | 1 |

**Supplementary Table 7. The number of metabolites in each metabolite score and its ex**

| Threshold | 95% | | |
|---|---|---|---|
| | No. of metabolites in the score | $R^2$_exploratory_set | $R^2$_validation_set |
| red meat | 114 | 0.23 | 0.17 |
| proc meat | 62 | 0.17 | 0.16 |
| poultry | 41 | 0.16 | 0.13 |

**plained variance of meat consumption using different thresholds in the exploratory and vali**

| 90% | | | 80% |
|---|---|---|---|
| No. of metabolites in the score | $R^2$_exploratory_set | $R^2$_validation_set | No. of metabolites in the score |
| 139 | 0.24 | 0.17 | 174 |
| 82 | 0.18 | 0.15 | 104 |
| 49 | 0.16 | 0.13 | 70 |

**idation sets**

| | | 50% | |
|---|---|---|---|
| $R^2$_exploratory_set | $R^2$_validation_set | No. of metabolites in the score | $R^2$_exploratory_set |
| 0.24 | 0.17 | 300 | 0.25 |
| 0.18 | 0.15 | 175 | 0.18 |
| 0.17 | 0.12 | 126 | 0.18 |

| |
|---|
| $R^2$_validation_set |
| 0.18 |
| 0.14 |
| 0.12 |

**Supplementary Table 8. Correlation matrix of top-ranked metabolites in the red meat metabc**

| | 1-palmityl-GPC | creatine |
|---|---|---|
| 1-palmityl-GPC (O-16:0) | 1 | 0.07 |
| creatine | 0.07 | 1 |
| trans-4-hydroxyproline | 0.07 | 0.21 |
| stearoylcarnitine | 0.05 | -0.08 |
| deoxycarnitine | 0.13 | -0.22 |
| trimethylamine N-oxide | 0 | 0.14 |
| 1-(1-enyl-stearoyl)-2-arachidonoyl-GPE (P-18:0/20:4)* | 0.27 | 0.21 |
| 1-(1-enyl-stearoyl)-2-arachidonoyl-GPC (P-18:0/20:4) | 0.3 | 0.08 |
| 1-(1-enyl-palmitoyl)-2-linoleoyl-GPC (P-16:0/18:2)* | 0.29 | 0.09 |
| 1-(1-enyl-stearoyl)-2-linoleoyl-GPC (P-18:0/18:2)* | 0.25 | 0.13 |
| 1-palmityl-2-arachidonoyl-GPC (O-16:0/20:4)* | 0.38 | 0.07 |

**olite score and identified in the trial**

| trans-4-hydroxy | stearoylcarnitin | deoxycarniti | trimethyla | 1-(1-enyl-st | 1-(1-enyl-st | 1-(1-enyl-pa | 1-(1-enyl-st |
|---|---|---|---|---|---|---|---|
| 0.07 | 0.05 | 0.13 | 0 | 0.27 | 0.3 | 0.29 | 0.25 |
| 0.21 | -0.08 | -0.22 | 0.14 | 0.21 | 0.08 | 0.09 | 0.13 |
| 1 | 0.1 | 0.21 | 0.14 | 0.25 | 0.13 | 0.1 | 0.09 |
| 0.1 | 1 | 0.27 | 0.04 | 0.25 | 0.17 | 0.12 | 0.17 |
| 0.21 | 0.27 | 1 | 0.15 | 0.12 | 0.12 | 0.08 | 0.08 |
| 0.14 | 0.04 | 0.15 | 1 | 0.08 | 0.06 | -0.02 | 0.03 |
| 0.25 | 0.25 | 0.12 | 0.08 | 1 | 0.68 | 0.41 | 0.38 |
| 0.13 | 0.17 | 0.12 | 0.06 | 0.68 | 1 | 0.51 | 0.49 |
| 0.1 | 0.12 | 0.08 | -0.02 | 0.41 | 0.51 | 1 | 0.74 |
| 0.09 | 0.17 | 0.08 | 0.03 | 0.38 | 0.49 | 0.74 | 1 |
| 0.18 | 0.19 | 0.13 | 0.01 | 0.65 | 0.63 | 0.21 | 0.14 |

| 1-palmityl-2-arachidonoyl-GPC (O-16:0/20:4)* |
| --- |
| 0.38 |
| 0.07 |
| 0.18 |
| 0.19 |
| 0.13 |
| 0.01 |
| 0.65 |
| 0.63 |
| 0.21 |
| 0.14 |
| 1 |

**Supplementary Table 9. The missing rates of metabolites in the red meat metabolite score in the exp**

| metabolites | BIOCHEMICAL | coef. In the score |
|---|---|---|
| s_M52475 | 1-(1-enyl-stearoyl)-2-arachidonoyl-GPE (P-18:0/20:4)* | 3.042 |
| s_M52613 | 1-(1-enyl-stearoyl)-2-arachidonoyl-GPC (P-18:0/20:4) | 2.765 |
| s_M52499 | 1-margaroyl-2-oleoyl-GPC (17:0/18:1)* | 2.750 |
| s_M32306 | trans-4-hydroxyproline | 2.672 |
| s_M42990 | verapamil | 2.528 |
| s_M46347 | X - 11381 | 2.296 |
| s_M40406 | trimethylamine N-oxide | 2.175 |
| s_M52682 | 1-(1-enyl-palmitoyl)-2-linoleoyl-GPC (P-16:0/18:2)* | 1.982 |
| s_M20458 | 1-palmityl-GPC (O-16:0) | 1.701 |
| s_M52470 | 1-palmitoyl-2-palmitoleoyl-GPC (16:0/16:1)* | 1.611 |
| s_M00513 | creatinine | 1.543 |
| s_M38296 | 17-methylstearate | 1.454 |
| s_M27718 | creatine | 1.430 |
| s_M37459 | ergothioneine | 1.376 |
| s_M33971 | 10-heptadecenoate (17:1n7) | 1.375 |
| s_M52433 | sphingomyelin (d18:1/15:0, d16:1/17:0)* | 1.278 |
| s_M52718 | 1-palmityl-2-arachidonoyl-GPC (O-16:0/20:4)* | 1.134 |
| s_M52712 | 1-(1-enyl-stearoyl)-2-linoleoyl-GPC (P-18:0/18:2)* | 1.055 |
| s_M44878 | methionine sulfone | 1.047 |
| s_M37231 | 1-docosapentaenoyl-GPC (22:5n3)* | 0.980 |
| s_M36747 | deoxycarnitine | 0.918 |
| s_M31787 | 3-carboxy-4-methyl-5-propyl-2-furanpropanoate (CMPF) | 0.911 |
| s_M00063 | cholesterol | 0.898 |
| s_M46500 | X - 02249 | 0.897 |
| s_M33950 | N-acetylphenylalanine | 0.879 |
| s_M15716 | imidazole lactate | 0.844 |
| s_M22036 | 2-hydroxyoctanoate | 0.838 |
| s_M43488 | N-acetylcarnosine | 0.796 |
| s_M42448 | 1-stearoyl-2-oleoyl-GPE (18:0/18:1) | 0.773 |
| s_M15506 | choline | 0.760 |
| s_M38595 | ranitidine | 0.735 |
| s_M46359 | X - 21315 | 0.708 |
| s_M47938 | X - 12731 | 0.699 |
| s_M46695 | X - 18913 | 0.691 |
| s_M35136 | 5-methyluridine (ribothymidine) | 0.676 |
| s_M36602 | 1-oleoyl-GPI (18:1)* | 0.665 |
| s_M22132 | alpha-hydroxyisocaproate | 0.657 |
| s_M36098 | 4-vinylphenol sulfate | 0.598 |
| s_M37185 | 5alpha-androstan-3alpha,17beta-diol monosulfate (2) | 0.577 |
| s_M44877 | N-palmitoyl-sphingosine (d18:1/16:0) | 0.571 |
| s_M52504 | X - 24309 | 0.560 |
| s_M43829 | gamma-glutamylvaline | 0.518 |
| s_M32827 | andro steroid monosulfate (1)* | 0.514 |
| s_M33943 | N-acetylglutamine | 0.503 |

| | | |
|---|---|---|
| s_M32388 | dodecanedioate | 0.502 |
| s_M39592 | S-methylcysteine | 0.490 |
| s_M47650 | X - 11483 | 0.480 |
| s_M52297 | X - 24293 | 0.476 |
| s_M35625 | 1-myristoylglycerol (14:0) | 0.475 |
| s_M46728 | X - 21659 | 0.452 |
| s_M36746 | 2-hydroxy-3-methylvalerate | 0.435 |
| s_M47804 | X - 16580 | 0.426 |
| s_M46487 | X - 21442 | 0.402 |
| s_M48195 | fructose | 0.397 |
| s_M17747 | sphingosine | 0.375 |
| s_M37496 | N-acetylputrescine | 0.367 |
| s_M34409 | stearoylcarnitine | 0.347 |
| s_M37443 | cysteine sulfinic acid | 0.316 |
| s_M46662 | X - 15492 | 0.313 |
| s_M46687 | X - 18779 | 0.310 |
| s_M46656 | X - 14662 | 0.288 |
| s_M48442 | 4-vinylguaiacol sulfate | 0.274 |
| s_M49459 | X - 23583 | 0.234 |
| s_M02342 | serotonin | 0.227 |
| s_M43255 | N-acetyl-1-methylhistidine* | 0.221 |
| s_M46689 | X - 17145 | -0.141 |
| s_M15958 | phenylacetate | -0.182 |
| s_M46521 | X - 11852 | -0.200 |
| s_M01573 | guanosine | -0.223 |
| s_M01508 | pantothenate | -0.237 |
| s_M31932 | propionylglycine | -0.242 |
| s_M46998 | X - 21821 | -0.249 |
| s_M35527 | 4-hydroxyhippurate | -0.308 |
| s_M46623 | X - 12729 | -0.335 |
| s_M47664 | X - 13658 | -0.343 |
| s_M49592 | X - 11315 | -0.349 |
| s_M48733 | vanillic alcohol sulfate | -0.383 |
| s_M47971 | X - 13729 | -0.389 |
| s_M49681 | X - 23782 | -0.404 |
| s_M49469 | X - 23593 | -0.407 |
| s_M18374 | methionine sulfoxide | -0.427 |
| s_M49647 | X - 23749 | -0.435 |
| s_M46358 | X - 12212 | -0.441 |
| s_M37752 | 13-HODE + 9-HODE | -0.447 |
| s_M22185 | N-acetylaspartate (NAA) | -0.447 |
| s_M18497 | taurocholate | -0.455 |
| s_M33009 | homostachydrine* | -0.467 |
| s_M01868 | cysteine | -0.470 |
| s_M15685 | 5-hydroxylysine | -0.474 |
| s_M57332 | X - 24061 | -0.507 |
| s_M46607 | X - 11849 | -0.510 |
| s_M46295 | X - 21286 | -0.516 |
| s_M32506 | 2-linoleoylglycerol (18:2) | -0.529 |
| s_M32553 | phenol sulfate | -0.535 |

| | | |
|---|---|---|
| s_M01572 | glycerate | -0.537 |
| s_M46325 | 1-arachidonoyl-GPA (20:4) | -0.539 |
| s_M52715 | 1-(1-enyl-palmitoyl)-2-myristoyl-GPC (P-16:0/14:0)* | -0.554 |
| s_M46367 | X - 11905 | -0.556 |
| s_M48460 | propyl 4-hydroxybenzoate sulfate | -0.559 |
| s_M46368 | X - 18914 | -0.566 |
| s_M46970 | X - 17676 | -0.578 |
| s_M43009 | furosemide | -0.604 |
| s_M47670 | X - 18899 | -0.613 |
| s_M39767 | quinine | -0.625 |
| s_M52672 | 1-(1-enyl-palmitoyl)-2-docosahexaenoyl-GPE (P-16:0/22:6)* | -0.628 |
| s_M47709 | X - 12339 | -0.633 |
| s_M00606 | uridine | -0.635 |
| s_M01126 | alanine | -0.635 |
| s_M45413 | O-sulfo-L-tyrosine | -0.652 |
| s_M32350 | 1-methylimidazoleacetate | -0.654 |
| s_M00512 | asparagine | -0.665 |
| s_M44620 | 4-acetylphenol sulfate | -0.668 |
| s_M35253 | 2-palmitoyl-GPC (16:0)* | -0.683 |
| s_M01566 | 3-aminoisobutyrate | -0.732 |
| s_M01669 | 4-hydroxyphenylpyruvate | -0.735 |
| s_M52716 | 1-(1-enyl-palmitoyl)-2-palmitoyl-GPC (P-16:0/16:0)* | -0.756 |
| s_M03141 | betaine | -0.757 |
| s_M52449 | 1-stearoyl-2-arachidonoyl-GPI (18:0/20:4) | -0.759 |
| s_M52476 | 1-(1-enyl-stearoyl)-2-docosahexaenoyl-GPE (P-18:0/22:6)* | -0.880 |
| s_M48491 | sphingomyelin (d18:1/20:1, d18:2/20:0)* | -0.965 |
| s_M33442 | pseudouridine | -1.006 |
| s_M52478 | 1-(1-enyl-palmitoyl)-2-oleoyl-GPC (P-16:0/18:1)* | -1.046 |
| s_M02132 | citrulline | -1.092 |
| s_M46225 | pyroglutamine* | -1.125 |
| s_M47790 | X - 13684 | -1.130 |
| s_M46355 | X - 09789 | -1.155 |
| s_M47787 | X - 14838 | -1.163 |
| s_M33228 | 1-arachidonoyl-GPC (20:4n6)* | -1.192 |
| s_M52434 | palmitoyl dihydrosphingomyelin (d18:0/16:0)* | -1.245 |
| s_M46458 | X - 12511 | -1.323 |
| s_M44675 | docosahexaenoate (DHA; 22:6n3) | -1.345 |
| s_M42450 | 1-stearoyl-2-arachidonoyl-GPC (18:0/20:4) | -1.383 |
| s_M00058 | glycine | -1.387 |
| s_M47154 | sphingomyelin (d18:2/14:0, d18:1/14:1)* | -1.460 |
| s_M37418 | 1-pentadecanoyl-GPC (15:0)* | -1.473 |
| s_M42446 | 1-palmitoyl-2-linoleoyl-GPC (16:0/18:2) | -1.591 |
| s_M46618 | X - 12442 | -1.701 |
| s_M42593 | triamterene | -1.879 |
| s_M42463 | sphingomyelin (d18:1/14:0, d16:1/16:0)* | -2.751 |

loratory set (combination of batch 2 and batch 3) and subgroups of red meat consumers and non-cons

| miss_rate_batch2 | miss_rate_batch3 | miss_rate_batch23 | miss_rate_batch2_redmeat_consumer | miss_rate_batch2_redmeat_nonconsumer | miss_rate_batch3_redmeat_consumer | miss_rate_batch3_redmeat_nonconsumer |
|---|---|---|---|---|---|---|
| 0.000 | 0.000 | 0.000 | 0.000 | 0.000 | 0.000 | 0.000 |
| 0.002 | 0.001 | 0.002 | 0.001 | 0.002 | 0.001 | 0.001 |
| 0.002 | 0.000 | 0.001 | 0.002 | 0.000 | 0.000 | 0.000 |
| 0.000 | 0.000 | 0.000 | 0.000 | 0.000 | 0.000 | 0.000 |
| 0.996 | 0.997 | 0.996 | 0.996 | 0.999 | 0.997 | 0.999 |
| 0.000 | 0.000 | 0.000 | 0.000 | 0.000 | 0.000 | 0.000 |
| 0.000 | 0.000 | 0.000 | 0.000 | 0.000 | 0.000 | 0.000 |
| 0.001 | 0.000 | 0.001 | 0.001 | 0.001 | 0.000 | 0.000 |
| 0.004 | 0.000 | 0.002 | 0.004 | 0.002 | 0.000 | 0.000 |
| 0.000 | 0.000 | 0.000 | 0.000 | 0.000 | 0.000 | 0.000 |
| 0.000 | 0.000 | 0.000 | 0.000 | 0.000 | 0.000 | 0.000 |
| 0.001 | 0.001 | 0.001 | 0.001 | 0.004 | 0.000 | 0.001 |
| 0.000 | 0.000 | 0.000 | 0.000 | 0.000 | 0.000 | 0.000 |
| 0.063 | 0.009 | 0.036 | 0.062 | 0.068 | 0.008 | 0.008 |
| 0.000 | 0.000 | 0.000 | 0.000 | 0.000 | 0.000 | 0.000 |
| 0.000 | 0.000 | 0.000 | 0.000 | 0.000 | 0.000 | 0.000 |
| 0.000 | 0.001 | 0.000 | 0.000 | 0.001 | 0.001 | 0.000 |
| 0.024 | 0.001 | 0.013 | 0.023 | 0.031 | 0.001 | 0.000 |
| 0.001 | 0.000 | 0.000 | 0.000 | 0.000 | 0.000 | 0.000 |
| 0.011 | 0.000 | 0.005 | 0.010 | 0.010 | 0.000 | 0.000 |
| 0.000 | 0.000 | 0.000 | 0.000 | 0.000 | 0.000 | 0.000 |
| 0.000 | 0.000 | 0.000 | 0.000 | 0.000 | 0.000 | 0.000 |
| 0.002 | 0.000 | 0.001 | 0.002 | 0.002 | 0.000 | 0.000 |
| 0.001 | 0.000 | 0.000 | 0.000 | 0.001 | 0.000 | 0.000 |
| 0.005 | 0.011 | 0.008 | 0.004 | 0.006 | 0.012 | 0.005 |
| 0.001 | 0.000 | 0.000 | 0.000 | 0.000 | 0.000 | 0.000 |
| 0.007 | 0.016 | 0.012 | 0.006 | 0.011 | 0.015 | 0.015 |
| 0.060 | 0.080 | 0.070 | 0.056 | 0.076 | 0.075 | 0.117 |
| 0.008 | 0.000 | 0.004 | 0.006 | 0.015 | 0.000 | 0.000 |
| 0.000 | 0.000 | 0.000 | 0.000 | 0.000 | 0.000 | 0.000 |
| 0.983 | 0.978 | 0.980 | 0.983 | 0.983 | 0.977 | 0.981 |
| 0.505 | 0.613 | 0.559 | 0.498 | 0.544 | 0.606 | 0.646 |
| 0.317 | 0.318 | 0.317 | 0.310 | 0.386 | 0.313 | 0.366 |
| 0.012 | 0.012 | 0.012 | 0.010 | 0.023 | 0.011 | 0.012 |
| 0.003 | 0.000 | 0.002 | 0.002 | 0.005 | 0.000 | 0.000 |
| 0.001 | 0.002 | 0.002 | 0.001 | 0.000 | 0.002 | 0.003 |
| 0.011 | 0.000 | 0.005 | 0.010 | 0.011 | 0.000 | 0.000 |
| 0.001 | 0.000 | 0.001 | 0.000 | 0.001 | 0.000 | 0.000 |
| 0.575 | 0.571 | 0.573 | 0.567 | 0.655 | 0.565 | 0.636 |
| 0.001 | 0.000 | 0.001 | 0.000 | 0.002 | 0.000 | 0.000 |
| 0.006 | 0.003 | 0.004 | 0.006 | 0.006 | 0.002 | 0.007 |
| 0.013 | 0.001 | 0.007 | 0.010 | 0.024 | 0.000 | 0.001 |
| 0.088 | 0.199 | 0.144 | 0.085 | 0.088 | 0.196 | 0.218 |
| 0.052 | 0.090 | 0.071 | 0.050 | 0.058 | 0.089 | 0.091 |

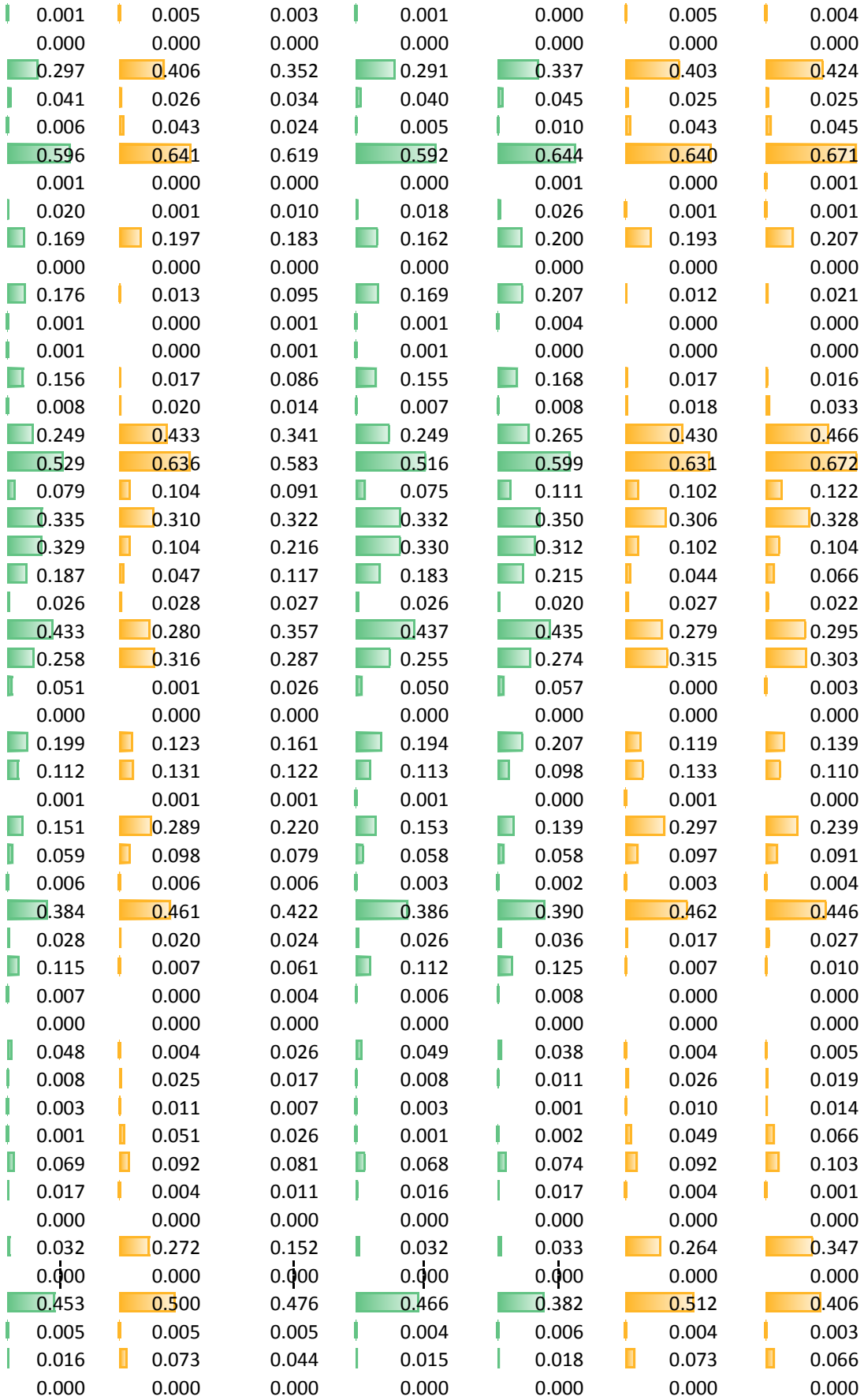
0.001 0.005 0.003 0.001 0.000 0.005 0.004
0.000 0.000 0.000 0.000 0.000 0.000 0.000
0.297 0.406 0.352 0.291 0.337 0.403 0.424
0.041 0.026 0.034 0.040 0.045 0.025 0.025
0.006 0.043 0.024 0.005 0.010 0.043 0.045
0.596 0.641 0.619 0.592 0.644 0.640 0.671
0.001 0.000 0.000 0.000 0.001 0.000 0.001
0.020 0.001 0.010 0.018 0.026 0.001 0.001
0.169 0.197 0.183 0.162 0.200 0.193 0.207
0.000 0.000 0.000 0.000 0.000 0.000 0.000
0.176 0.013 0.095 0.169 0.207 0.012 0.021
0.001 0.000 0.001 0.001 0.004 0.000 0.000
0.001 0.000 0.001 0.001 0.000 0.000 0.000
0.156 0.017 0.086 0.155 0.168 0.017 0.016
0.008 0.020 0.014 0.007 0.008 0.018 0.033
0.249 0.433 0.341 0.249 0.265 0.430 0.466
0.529 0.636 0.583 0.516 0.599 0.631 0.672
0.079 0.104 0.091 0.075 0.111 0.102 0.122
0.335 0.310 0.322 0.332 0.350 0.306 0.328
0.329 0.104 0.216 0.330 0.312 0.102 0.104
0.187 0.047 0.117 0.183 0.215 0.044 0.066
0.026 0.028 0.027 0.026 0.020 0.027 0.022
0.433 0.280 0.357 0.437 0.435 0.279 0.295
0.258 0.316 0.287 0.255 0.274 0.315 0.303
0.051 0.001 0.026 0.050 0.057 0.000 0.003
0.000 0.000 0.000 0.000 0.000 0.000 0.000
0.199 0.123 0.161 0.194 0.207 0.119 0.139
0.112 0.131 0.122 0.113 0.098 0.133 0.110
0.001 0.001 0.001 0.001 0.000 0.001 0.000
0.151 0.289 0.220 0.153 0.139 0.297 0.239
0.059 0.098 0.079 0.058 0.058 0.097 0.091
0.006 0.006 0.006 0.003 0.002 0.003 0.004
0.384 0.461 0.422 0.386 0.390 0.462 0.446
0.028 0.020 0.024 0.026 0.036 0.017 0.027
0.115 0.007 0.061 0.112 0.125 0.007 0.010
0.007 0.000 0.004 0.006 0.008 0.000 0.000
0.000 0.000 0.000 0.000 0.000 0.000 0.000
0.048 0.004 0.026 0.049 0.038 0.004 0.005
0.008 0.025 0.017 0.008 0.011 0.026 0.019
0.003 0.011 0.007 0.003 0.001 0.010 0.014
0.001 0.051 0.026 0.001 0.002 0.049 0.066
0.069 0.092 0.081 0.068 0.074 0.092 0.103
0.017 0.004 0.011 0.016 0.017 0.004 0.001
0.000 0.000 0.000 0.000 0.000 0.000 0.000
0.032 0.272 0.152 0.032 0.033 0.264 0.347
0.000 0.000 0.000 0.000 0.000 0.000 0.000
0.453 0.500 0.476 0.466 0.382 0.512 0.406
0.005 0.005 0.005 0.004 0.006 0.004 0.003
0.016 0.073 0.044 0.015 0.018 0.073 0.066
0.000 0.000 0.000 0.000 0.000 0.000 0.000

| | | | | | | |
|---|---|---|---|---|---|---|
| 0.001 | 0.000 | 0.000 | 0.000 | 0.000 | 0.000 | 0.000 |
| 0.030 | 0.040 | 0.035 | 0.031 | 0.021 | 0.042 | 0.034 |
| 0.179 | 0.041 | 0.110 | 0.173 | 0.183 | 0.036 | 0.056 |
| 0.011 | 0.017 | 0.014 | 0.011 | 0.008 | 0.015 | 0.025 |
| 0.457 | 0.562 | 0.509 | 0.466 | 0.374 | 0.574 | 0.471 |
| 0.002 | 0.001 | 0.002 | 0.001 | 0.001 | 0.001 | 0.000 |
| 0.049 | 0.079 | 0.064 | 0.049 | 0.050 | 0.076 | 0.089 |
| 0.973 | 0.980 | 0.976 | 0.974 | 0.985 | 0.981 | 0.988 |
| 0.003 | 0.002 | 0.002 | 0.002 | 0.001 | 0.002 | 0.005 |
| 0.888 | 0.846 | 0.867 | 0.886 | 0.900 | 0.845 | 0.856 |
| 0.001 | 0.000 | 0.000 | 0.000 | 0.000 | 0.000 | 0.000 |
| 0.081 | 0.000 | 0.041 | 0.082 | 0.070 | 0.065 | 0.074 |
| 0.001 | 0.000 | 0.001 | 0.001 | 0.000 | 0.000 | 0.000 |
| 0.000 | 0.000 | 0.000 | 0.000 | 0.000 | 0.000 | 0.000 |
| 0.000 | 0.000 | 0.000 | 0.000 | 0.000 | 0.000 | 0.000 |
| 0.001 | 0.001 | 0.001 | 0.000 | 0.000 | 0.001 | 0.000 |
| 0.000 | 0.000 | 0.000 | 0.000 | 0.000 | 0.000 | 0.000 |
| 0.029 | 0.097 | 0.063 | 0.028 | 0.037 | 0.099 | 0.088 |
| 0.000 | 0.000 | 0.000 | 0.000 | 0.000 | 0.000 | 0.000 |
| 0.001 | 0.000 | 0.000 | 0.000 | 0.000 | 0.000 | 0.000 |
| 0.001 | 0.000 | 0.000 | 0.001 | 0.000 | 0.000 | 0.000 |
| 0.013 | 0.000 | 0.006 | 0.011 | 0.013 | 0.000 | 0.000 |
| 0.000 | 0.000 | 0.000 | 0.000 | 0.000 | 0.000 | 0.000 |
| 0.000 | 0.000 | 0.000 | 0.000 | 0.000 | 0.000 | 0.000 |
| 0.002 | 0.000 | 0.001 | 0.002 | 0.001 | 0.000 | 0.000 |
| 0.000 | 0.000 | 0.000 | 0.000 | 0.000 | 0.000 | 0.000 |
| 0.000 | 0.000 | 0.000 | 0.000 | 0.000 | 0.000 | 0.000 |
| 0.007 | 0.000 | 0.003 | 0.007 | 0.005 | 0.000 | 0.000 |
| 0.000 | 0.000 | 0.000 | 0.000 | 0.000 | 0.000 | 0.000 |
| 0.001 | 0.000 | 0.000 | 0.000 | 0.000 | 0.000 | 0.000 |
| 0.002 | 0.001 | 0.002 | 0.002 | 0.001 | 0.001 | 0.000 |
| 0.000 | 0.000 | 0.000 | 0.000 | 0.000 | 0.000 | 0.000 |
| 0.008 | 0.001 | 0.004 | 0.006 | 0.018 | 0.001 | 0.000 |
| 0.000 | 0.000 | 0.000 | 0.000 | 0.000 | 0.000 | 0.000 |
| 0.000 | 0.000 | 0.000 | 0.000 | 0.000 | 0.000 | 0.000 |
| 0.010 | 0.004 | 0.007 | 0.010 | 0.012 | 0.004 | 0.005 |
| 0.000 | 0.000 | 0.000 | 0.000 | 0.000 | 0.000 | 0.000 |
| 0.000 | 0.000 | 0.000 | 0.000 | 0.000 | 0.000 | 0.000 |
| 0.000 | 0.000 | 0.000 | 0.000 | 0.000 | 0.000 | 0.000 |
| 0.001 | 0.000 | 0.000 | 0.000 | 0.000 | 0.000 | 0.000 |
| 0.001 | 0.000 | 0.000 | 0.000 | 0.000 | 0.000 | 0.000 |
| 0.000 | 0.000 | 0.000 | 0.000 | 0.000 | 0.000 | 0.000 |
| 0.001 | 0.005 | 0.003 | 0.000 | 0.000 | 0.005 | 0.005 |
| 0.996 | 0.995 | 0.995 | 0.996 | 0.998 | 0.995 | 0.996 |
| 0.000 | 0.000 | 0.000 | 0.000 | 0.000 | 0.000 | 0.000 |
